# Connect Four: Tetraarylated Dihydropentalenes and Triarylated Monocyclic Pentafulvenes from Cyclopentadienes and Enones

**DOI:** 10.1021/acs.joc.2c01507

**Published:** 2022-10-05

**Authors:** Niko A. Jenek, Marek Balschun, Stuart M. Boyt, Ulrich Hintermair

**Affiliations:** † Department of Chemistry, University of Bath, Claverton Down, Bath BA2 7AY, U.K.; ‡ Centre for Sustainable and Circular Technologies, University of Bath, Claverton Down, Bath BA2 7AY, U.K.

## Abstract

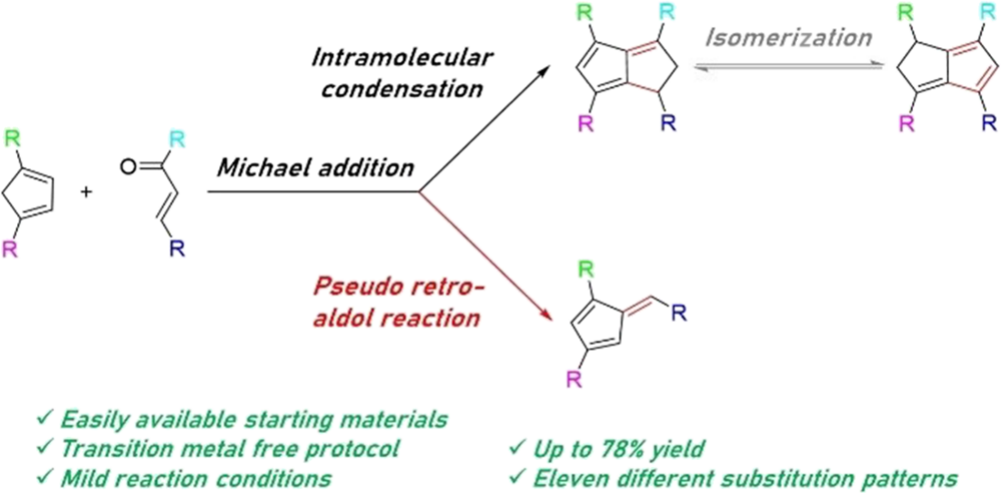

In search of novel pentalenide ligands for use in organometallic chemistry and homogeneous catalysis, we report the scope of a straightforward base-promoted Michael annulation of cyclopentadienes with α,β-unsaturated ketones that allows the introduction of symmetrical as well as unsymmetrical aryl and alkyl substitution patterns including electron-donating as well as electron-withdrawing substituents. More than 16 examples of various isomers of 1,3,4,6-tetraarylated dihydropentalenes have been synthesized in isolated yields of up to 78%, representing a substantial expansion of the range of dihydropentalene scaffolds known to date. Double bond isomerization between the two pentacyclic rings in 1,2-dihydropentalenes with electronically different substituents occurred depending on the polarization of the molecule. The melting points of the air-stable dihydropentalenes decrease, and their solubilities in organic solvents improve with increasing substitution and decreasing symmetry of the molecule. A competitive pseudo-retro-aldol pathway produces 1,3,6-triarylated monocyclic pentafulvenes as side products in yields of 9–68%, which can be cleanly isolated (8 new examples) and used for other synthetic purposes, including separate cyclization to other dihydropentalenes.

## 1 Introduction

**Scheme 1:**
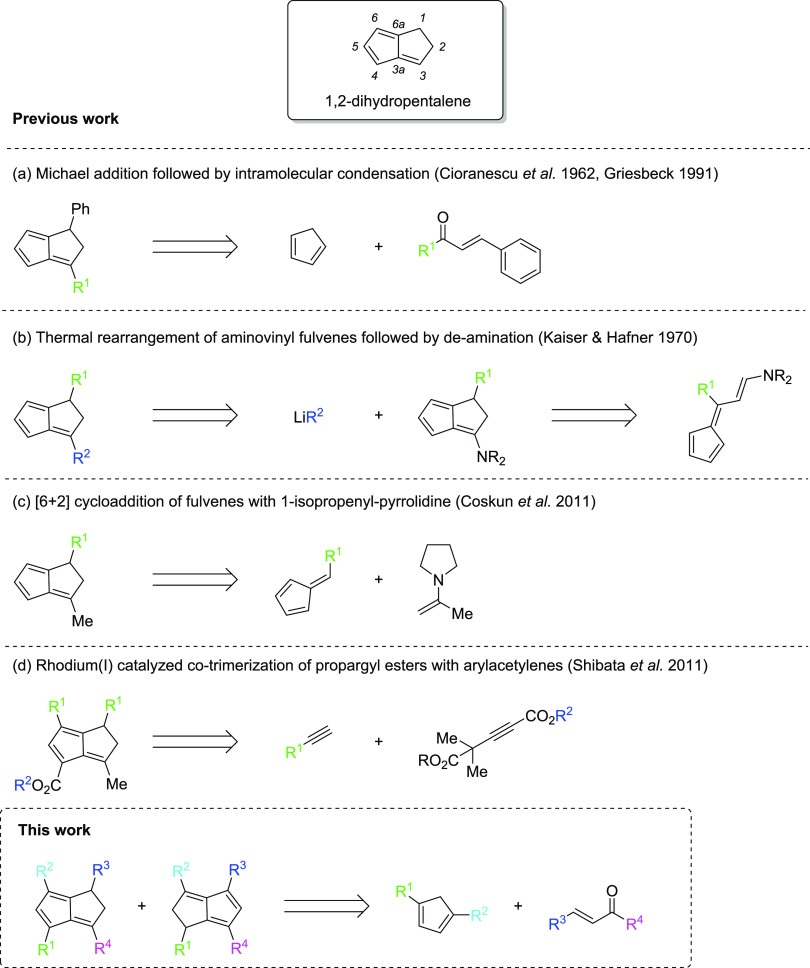
1,2-Dihydropentalene Nomenclature and Syntheses

Due to their unsaturated polyquinane framework, 1,2-dihydropentalenes (**PnH_2_**) have been widely utilized in organic photochemistry,^1^ cycloaddition reactions,^2^ and natural product syntheses.^3^**PnH_2_** may serve as precursors to pentalenes, which have been of interest for many decades due to their 8 π anti-aromatic character.^4−6^**PnH_2_** have also found use in organometallic chemistry, where they serve as precursors for dianionic pentalenide ligands.^7^ These bicyclic 10 π aromatic compounds have generated interest due to their unique coordination properties, including the formation of electronically coupled bimetallic complexes.^4,8^ Over the last six decades, few approaches for the synthesis of dihydropentalenes have been demonstrated.^7^ Several reports have described the synthesis of the parent, unsubstituted dihydropentalene C_8_H_8_ via radical rearrangements from precursors like isodicyclopentadiene, cyclooctatetraene, or cycloheptatriene induced by photochemical or pyrolytic techniques.^4^ However, due to the high specificity of these skeletal rearrangement reactions, there is little scope for the targeted introduction of substituents to access selectively functionalized dihydropentalenes. The first example of a substituted dihydropentalene came from Cioranescu et al. in 1962 who utilized an annulation reaction based on a Michael addition between cyclopentadiene (**CpH**; C_5_H_6_) and an α,β-unsaturated ketone followed by intramolecular condensation (Scheme 1a).^9^ This approach was later adapted by Griesbeck as well as by Gotoh et al., with the latter demonstrating α,β-unsaturated aldehydes as suitable substrates to give mono-substituted dihydropentalenes with high selectivity (Scheme 1a).^10,11^ However, all of their work employed unsubstituted **CpH** as the nucleophile precursor. Another approach was demonstrated by Hafner and Kaiser via a thermal intramolecular condensation of aminovinylfulvenes (Scheme 1b).^12^ This method was subsequently used intramolecularly by Wu and Houk to create fused tricyclopentanoids containing the dihydropentalene moiety.^13^ More recently, Coskun et al. used monocyclic pentafulvenes (**Fv**) for the synthesis of 3-methyl-substituted **PnH_2_** (Scheme 1c) through a similar cyclization reaction,^14^ including one notable example involving 1,4-diphenyl-cyclopentadiene (**Ph_2_CpH**) to furnish a rare example of a 1,3,4,6-substituted **PnH_2_**. A different approach giving access to tetrasubstituted **PnH_2_** with some variability in the 1 and 6 positions was demonstrated by Shibata et al. using a Rh-catalyzed cyclization of propargyl esters with arylacetylenes.^15^ Possible substitution patterns remained restricted to a methyl group in 3 position and carboxyl groups in 4 position, with only arylated groups in 1 and 6 positions (Scheme 1d).

We recently reported the straightforward synthesis of 1,3,4,6-tetraphenyl-1,2-dihydropentalene (**Ph_4_PnH_2_**) based on a modification of the strategies developed by Cioranescu et al. and Griesbeck. Intrigued by the simplicity of this reaction and the good yields obtained under optimized conditions, we chose to investigate the scope and limitations of possible substitution patterns that may be accessed by this approach further. Here, we report the results of our study that demonstrate control over all four substitution sites in 1,3,4,6-tetrafunctionalized dihydropentalenes without the need for precious metal catalysts or special pyrolysis equipment (Scheme 1).

## 2 Results and Discussion

### 2.1 Scope of 1,4-diarylated Cyclopentadiene Synthesis

**Scheme 2:**
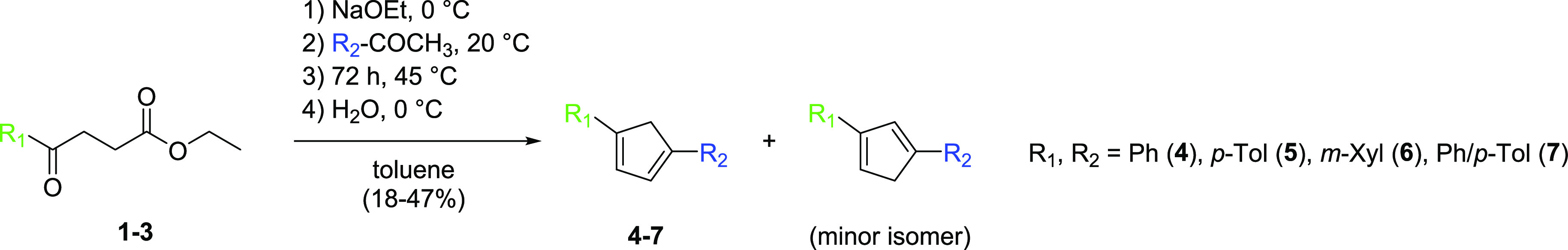
Synthesis of 1,4-Diarylated Cyclopentadienes

Since in our previous work **1,4-Ph_2_CpH** displayed good reactivity toward 1,3-diphenylprop-2-en-1-one with pyrrolidine in refluxing MeOH/toluene,^16^ we decided to synthesize other 1,4-diarylated cyclopentadienes with different substitution patterns to test their utility in furnishing tetraarylated dihydropentalenes with modified substituents in one of the two five-membered rings. Using a modification of the method reported by Drake and Adams, we synthesized **Ph_2_CpH** (**4**),^17^ 1,4-di-*p*-tolylcyclopenta-1,3-diene (***p*-Tol_2_CpH**; **5**),^18^ the novel 1,4-bis(3,5-dimethylphenyl)cyclopenta-1,3-diene (***m*-Xyl_2_CpH**; **6**), and the unsymmetrically substituted 1-phenyl-4-*p*-tolyl-cyclopenta-1,3-diene (**(Ph)(*p*-Tol)CpH**; **7**)^17^ in 18–47% yield from their corresponding carboxylic acids **1–3** (Scheme 2). While cyclopentadienes **4, 5**, and **7** were obtained predominantly as the 1,4-isomer (1,4/1,3 ratio >10:1), **6** displayed a lower 1,4/1,3 isomer ratio of 10:3. All cyclopentadienes were air-stable, golden-yellow crystalline solids with no sign of degradation after 1 year of storage under atmospheric conditions. In solution, however, progressive decomposition was observed to occur over the course of several days to weeks under air.

We also investigated the synthesis of 1,4-dimesitylcyclopenta-1,3-diene (**Mes_2_CpH**) using the same protocol. While we were able to synthesize the precursor ethyl-4-mesityl-4-oxobutanoate in yields of 60%, no **Mes_2_CpH** product could be isolated from its attempted cyclization with 2,4,6-trimethylacetophenone (as shown for **4–7** in Scheme 2). Even when reducing the steric demand to *ortho*-tolyl, the attempted cyclization of ethyl-4-oxo-4-(*o*-tolyl)butanoate with 1-(*o*-tolyl)ethan-1-one did not lead to the desired cyclopentadiene (whereas the reaction with *para*-tolyl did proceed well), showing aromatic substituents in *ortho* positions to be a hindrance in this cyclizing condensation reaction. Alternative routes using palladium catalysis have been used successfully to introduce up to four mesityl substituents into **CpH**,^19^ so the limitations encountered with the route shown in Scheme 2 were likely kinetic in nature. More remote functionalization in the *meta* position, such as the methyl groups in *m*-xylyl **6** worked with a moderate yield of 18%.

### 2.2 Synthesis of Symmetrically 1,3,4,6-Tetraarylated Dihydropentalenes—Competition with 1,3,6-Triarylated Monocyclic Pentafulvene Formation

**Scheme 3:**
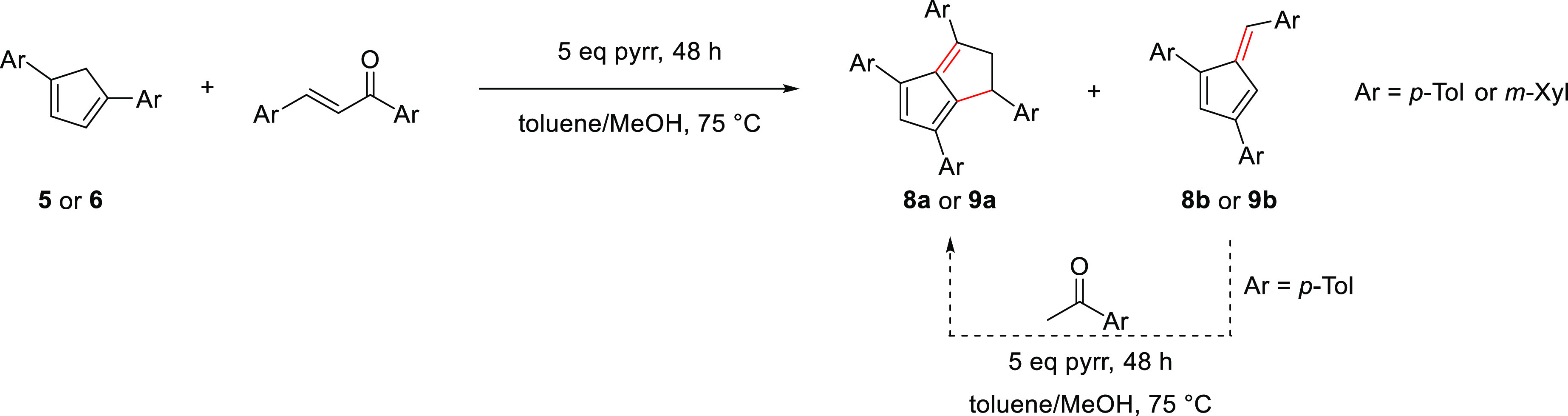
Synthesis of Symmetrically 1,3,4,6-Tetraarylated Dihydropentalenes

Pyrrolidine has been shown to be superior to other bases in facilitating **PnH_2_** formation from **Ph_2_CpH** and chalcone.^12,16^ As discussed in more detail by Hayashi et al.,^14,20^ this effect is likely due to a double activation of both substrates, with pyrrolidine increasing the nucleophilicity of the cyclopentadiene by deprotonation while increasing the electrophilicity of the enone by in situ iminium formation. Applying our previously optimized conditions for the synthesis of 1,3,4,6-**Ph**_4_**PnH_2_** (1 equiv R_2_CpH, 1.1 equiv enone, 1.1 equiv pyrrolidine, toluene/MeOH at 70 °C for 40 h)^16^ to the reaction of ***p*-Tol_2_CpH** with 1,3-di-*p*-tolylprop-2-en-1-one initially showed no noticeable reaction progress after 40 h. Applying slightly more forcing conditions of 75 °C with 5 equiv of pyrrolidine did produce a characteristic color change within 2 h and showed complete conversion after 46 h (Scheme 3). The higher demand for pyrrolidine could be a result of the slightly higher p*K*_a_ of ***p*-Tol_2_CpH** compared to **Ph_2_CpH**.

As observed with **Ph_4_PnH_2_**,^16^ no nucleophilic attack from the C5 position of the cyclopentadiene on the enone seemed to take place, presumably due to steric hindrance by the substituents in the flanking 1,4 positions. Purification of the crude reaction mixture via column chromatography on silica using 5:1 cyclohexane/toluene as the eluent mixture yielded two products. The first fraction was identified as 1,3,6-tri-*p*-toluylpentafulvene (***p*-Tol_3_Fv, 8b**), obtained as a dark orange-red solid in 28% yield. The formation of a pentafulvene from the reaction of a **CpH** with an enone likely reflects a retro-Aldol cleavage after the initial Michael addition that competes with the cyclizing condensation to furnish the **PnH_2_** (see Section 2.3).^21^ The second fraction contained the new 1,3,4,6-tetra-*p*-tolyl-1,2-dihydropentalene (***p*-Tol_4_PnH_2_, 8a**) as a cherry-red solid in 36% yield. Like **Ph_4_PnH_2_**,^16^ the former was obtained exclusively as the 1,2-isomer, displaying a similar ^1^H NMR coupling pattern of the proton at C1 (4.56 ppm) and the two protons at C2 (4.10 and 3.42 ppm) with similar ^13^C NMR shifts of the C1 (42.3 ppm) and C2 (55.4 ppm; for details, see Figure S12). Although a heavier molecule, the air-stable **8a** possessed a melting point of 84 °C lower than **Ph_4_PnH_2_** (***p*-Tol_4_PnH_2_** 96–97 °C; **Ph_4_PnH_2_** 180–181 °C), indicating a decreased lattice energy due to less favorable packing in the solid state. This trend was also confirmed by an observed increase in solubility of **8a** relative to **Ph_4_PnH_2_** and comparing the melting point of ***p*-Tol_4_PnH_2_** with its corresponding fulvene **8b**, which possesses a lower molecular weight but melts at 105 °C. Since **Ph_3_Fv** successfully underwent cyclization with acetophenone via [6 + 2] addition to give **Ph_4_PnH_2_** in our previous work,^16^ we investigated the analogous reaction between **8b** and *para*-methylacetophenone in MeOH/toluene to see if the isolated pentafulvene side product may be converted into a dihydropentalene separately. While ***p*-Tol_3_Fv** was nearly fully consumed after 44 h, ***p*-Tol_4_PnH_2_** was only obtained in 5% yield. Since pentafulvene formation represented a competitive side reaction, we briefly investigated the possibility of increasing dihydropentalene selectivity. Using one equivalent of NaO*t*Bu in dry THF to activate the cyclopentadiene and conducting the cyclizing double condensation with the enone at room temperature yielded **8a** in 33% yield and **8b** in 7%. This result shows that using a stoichiometric amount of a strong, ionic base at low temperatures may lower the amount of fulvene formation (4.7:1 DHP/Fv ratio *versus* 1.3:1 using excess pyrrolidine in refluxing MeOH/toluene) but may not completely suppress it. As the pyrrolidine route generally produced higher yields, we chose to apply this protocol for all other substrates discussed in the following, but note that pentafulvene selectivity may be decreased using alternative reaction conditions if desired (see Section S1).

**Scheme 4:**
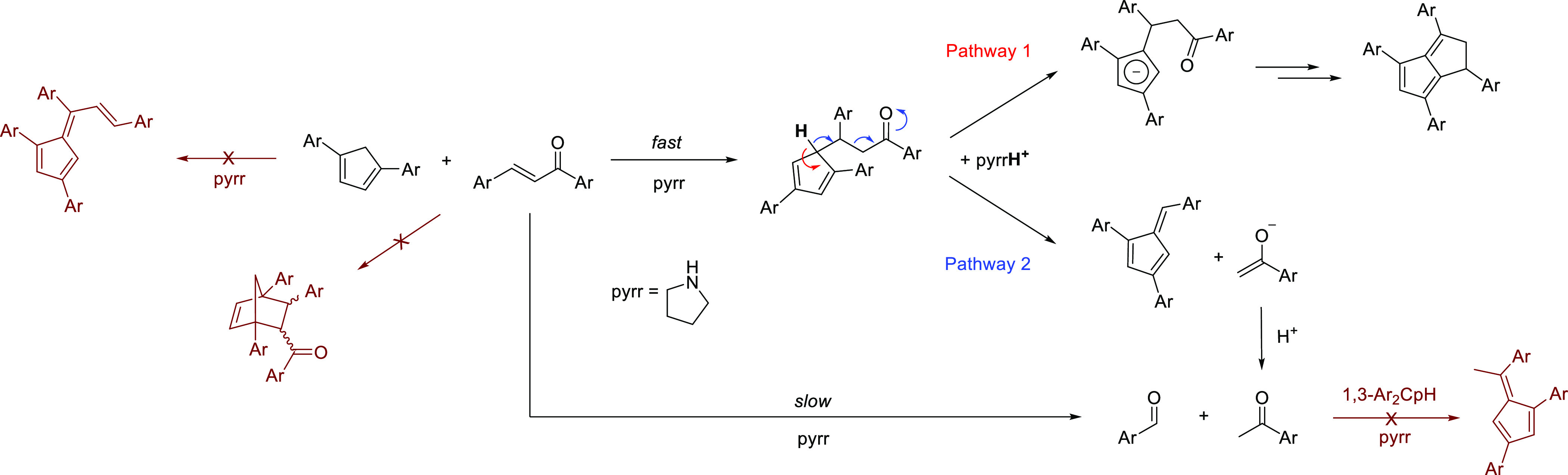
Pentafulvene and Dihydropentalene Formation Pathways from Cyclopentadienes and Enones (Dark Red = Not Observed)

As 6-substituted monocyclic pentafulvenes are typically formed from the reaction between **CpH** and aldehydes,^22−25^ we investigated the retro-aldol cleavage of the enones employed by heating 1,3-di-*o*-tolylprop-2-en-1-one (see Section 2.4 for the corresponding **PnH_2_** synthesis attempt) to 75 °C with an excess of pyrrolidine in 1:1 MeOH/toluene. ^1^H NMR data indicated the formation of an aldehyde by the appearance of a singlet at 10.28 ppm formed in a 25:1 ratio of enone/aldehyde after 44 h, which is slow compared to the initial Michael addition of a **Cp(H)** as indicated by a significant color change within 2 h under these conditions. Furthermore, if the enone would undergo a free retro-aldol cleavage during **Pn_2_H** synthesis, there would also be an equal amount of an acetophenone derivate that could react with free **R_2_Cp(H),** forming a 1,3,6,6-tetrasubstituted pentafulvene which has never been observed in any of our reactions. We therefore suggest that the pentafulvenes obtained formed through a competing pathway after the initial 1,4 addition of the **Cp(H)** to the enone (Scheme 4).

After the conjugate addition, the 1,2,4-trisubstituted **CpH** formed will be deprotonated by the base in the acidic 1 position. The negative charge may then either be conjugated into the five-membered ring to enable a second, cyclizing nucleophilic attack of the **Cp^–^** on the ketone to furnish a dihydropentalene after elimination of water (pathway 1, Scheme 4) or lead to an exocyclic double bond as part of a retro-aldol C–C cleavage to give a pentafulvene (pathway 2, Scheme 4). The observation of increased amounts of pentafulvene formation with sterically demanding aryl substituents (e.g., *o*-tolyl; see above) is consistent with this proposal, as steric strain between the substituents in the 1,2,4-trisubstituted **CpH** intermediate would favor pathway 2 over pathway 1 (Scheme 4). To our best knowledge, there is only one previous example of such a pseudo retro-aldol reaction following a Michael addition between enones and cyclopentadienes in the literature: the condensation of **CpH** with mesityl oxide in methanol and pyrrolidine as the base producing 6,6-dimethylpentafulvene.^21^ In addition, we were also not able to detect any evidence of 1,2-addition or Diels-Alder products as the main competitors of the desired 1,4-addition (dark red; Scheme 4).^14,21^ Note that pathway 1 could have a contribution from transient iminium ion formation from the activation of the enone by pyrrolidine.^14^ However, as dihydropentalene formation may also be induced by, for example, alkoxide bases,^9,16,26^ the anionic **Cp^–^** route is likely the main reaction pathway.

Reacting ***m*-Xyl_2_CpH** with 1,3-bis(3,5-dimethylphenyl)prop-2-en-1-one (synthesized via an adaption of Volz and Hassler’s 1,3-bis(3,5-di-*tert*-butylphenyl)prop-2-en-1-one)^27^ using the more forcing conditions of the successful ***p*-Tol_4_PnH_2_** synthesis (Scheme 3) showed full conversion of both reagents by ^1^H NMR spectroscopy after 46.5 h. Chromatographic purification of the crude on silica using 10:1 cyclohexane/toluene produced separate fractions of the corresponding 1,3,6-tris(3,5-dimethyl-phenyl)-pentafulvene (***m*-Xyl_3_Fv, 9b**) as a dark orange-red solid in 25% yield, followed by the new 1,3,4,6-tetrakis(3,5-dimethylphenyl)-1,2-dihydropentalene (***m*-Xyl_4_PnH_2,_ 9a**) in 35% yield as a cherry-red solid. As with **Ph_4_PnH_2_** and ***p*-Tol_4_PnH_2_**, the ***m*-Xyl_4_PnH_2_** product formed exclusively as the 1,2-isomer, displaying the characteristic coupling pattern of the proton at C1 (4.41 ppm) and the two protons at C2 (4.04 and 3.48 ppm) in the ^1^H NMR spectrum and chemical shifts of C1 (42.9 ppm) and C2 (55.0 ppm; for details, see Figures S16 and S17) in the ^13^C NMR spectrum. Similar to ***p*-Tol_4_PnH_2_**, the air-stable ***m*-Xyl_4_PnH_2_** displayed a >80 °C lower melting point (95–96 °C) and a higher solubility compared to **Ph_4_PnH_2_**. Since the homobimetallic alkali metal salts of the pentalenide derived from **Ph_4_PnH_2_** are known to be of very low solubility,^16^ this melting point depression caused by the introduction of methyl groups in ***p*-Tol_4_PnH_2_** and ***m*-Xyl_4_PnH_2_** compared to **Ph_4_PnH_2_** could prove useful in enhancing the solubility, and thus synthetic utility, of their corresponding pentalenide salts in the future. We also briefly investigated the UV–visible absorption properties of **Ph_4_PnH_2_**, ***p*-Tol_4_PnH_2_** (**8a**), and ***m*-Xyl_4_PnH_2_** (**9a**) as well as the corresponding pentafulvenes **Ph_3_Fv**, ***p*-Tol_3_Fv** (**8b**), and ***m*-Xyl_3_Fv** (**9b**) at 10^–5^ mol/L in dichloromethane solution (Figures S59–61). Consistent with the visual appearance of the pure solids, all dihydropentalenes showed higher molar extinction coefficients than their corresponding pentafulvenes, presumably due to the higher rigidity of the bicyclic framework compared to the monocyclic pentafulvene. Comparing substituent effects, introduction of four tolyl groups in place of phenyl substituents increased the molar absorptivity in the dihydropentalenes (ε = 70,700 M^–1^ cm^–1^ at λ_max_ = 287 nm for **8a**), whereas xylyl substituents lowered molar absorptivity (ε = 26,000 M^–1^ cm^–1^ at λ_max_ = 291 nm for **9a**). The same trend prevailed in the trisubstituted pentafulvenes which had absorption maxima at slightly higher energy than their corresponding dihydropentalenes (**Ph_3_Fv** = 266 nm, **8b** = 274 nm, **9b** = 268 nm). None of the pentafulvenes investigated showed any of the broad absorptions in the visible range at 400–550 nm reported for the very strongly absorbing hydropentalenide **Na[Ph_4_PnH]**.^16^

### 2.3 Synthesis of Unsymmetrically 1,3,4,6-Tetraarylated Dihydropentalenes—Double Bond Isomerization

**Scheme 5:**
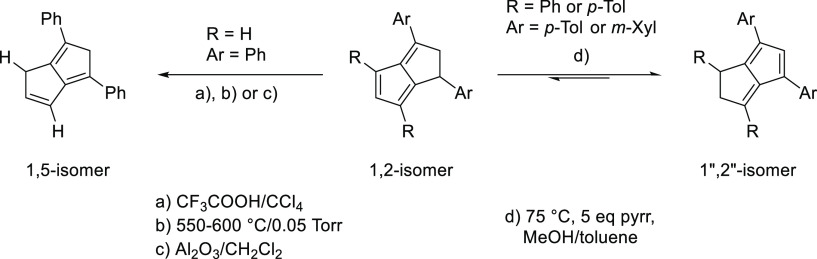
Double Bond Isomerization in Arylated Dihydropentalenes

In anticipation of accessing bicyclic systems with different electronic properties in each five-membered ring, we investigated the possibility of synthesizing dihydropentalenes of the overall formula **1,3,4,6-R_1_R_1_R_2_R_2_PnH_2_**. Reacting **Ph_2_CpH** with 1,3-di-*p*-tolylprop-2-en-1-one as the electrophile under the conditions shown in Scheme 3 yielded the corresponding 1,3-diphenyl-6-*p*-tolylpentafulvene (**10b**) in 29% yield along with the new dihydropentalene **(*p*Tol)_2_(Ph)_2_PnH_2_** (**10a**) in 66% yield. Similarly, the analogous reaction of **Ph_2_CpH** with 1,3-bis(3,5-dimethylphenyl)prop-2-en-1-one produced 1,3-diphenyl-6-(3,5-dimethylphenyl)pentafulvene (**11b**) in 10% yield with 48% isolated yield of the unsymmetrically substituted **(*m*Xyl)_2_(Ph)_2_PnH_2_** (**11a**). Even the reaction of ***p*Tol_2_CpH** with 1,3-bis(3,5-dimethylphenyl)prop-2-en-1-one delivered a similar outcome, producing the pentafulvene 1,3-di-*p*-tolyl-6-(3,5-dimethylphenyl)pentafulvene (**12b**) in 25% alongside the new **(*m*Xyl)_2_(*p*Tol)_2_PnH_2_** (**12a**) in 51% isolated yield. While mass spectrometry confirmed the presence of one dihydropentalene in all cases, NMR data indicated the presence of two isomeric 1,2-dihydropentalenes in **10aa/11aa/12aa** and **10ab/11ab/12ab** as confirmed by 2D correlation techniques and DOSY analysis (Scheme 5 and Figures S20–S22, S24–S26, and S29–S31). ^1^H and ^13^C HMBC spectra showed **1,3-Ar_2_-4,6-R_2_-PnH_2_** to be the major isomer in all three cases (Table 1).

**Table 1:** Post-Synthetic Isomer Ratios in Unsymmetrically Tetraarylated Dihydropentalenes

**R**	**Ar**	**1,3-Ar_2_–4,6-R_2_-PnH_2_**/**1,3-R_2_–4,6-Ar_2_-PnH_2_ ratio**
Phenyl	*p*-Tolyl	10:3
Phenyl	*m*-Xylyl	10:6
*p*-Tolyl	*m*-Xylyl	10:4

Previous reports have shown the isomerization of 1,3-disubstituted **PnH_2_** as well as unsubstituted **PnH_2_** into their corresponding 1,5 double bond isomers and discussed the thermodynamics of rearrangements between the 1,4, 1,5 and 1,6 double bond isomers to identify the 1,2 and 1,5 isomers as thermodynamically favored.^10,28−30^ In the case of unsymmetrical 1,3,4,6-tetrasubstitution, there exist two fulvenic 1,2 double bond isomers that interconvert under basic reaction conditions at 75 °C, redistributing the two saturated sp^3^ carbons into the pentacyclic ring of higher electron density. No further change in the isomer ratio observed post-reaction occurred over several days in CDCl_3_ at room temperature, but gradually heating the **12aa/12ab** post-synthetic isomer mixture of 10:4 in MeOH/toluene with excess pyrrolidine to 105 °C over 2 h induced a change in the **12aa/12ab** isomer distribution to 1:1. Further heating over multiple hours did not change the ratio, indicating the 1,2-dihydropentalene isomer distributions obtained from the synthesis (Table 1) to be kinetically limited. In the context of using 1,2-dihydropentalene as precursors to pentalenides, the distribution of isomers is irrelevant as long as both are effectively deprotonated to the same coplanar, dianionic 10 π aromatic system.

### 2.4 Designing Tetra-Substituted Dihydropentalenes—Scope and Limitations

**Scheme 6:**
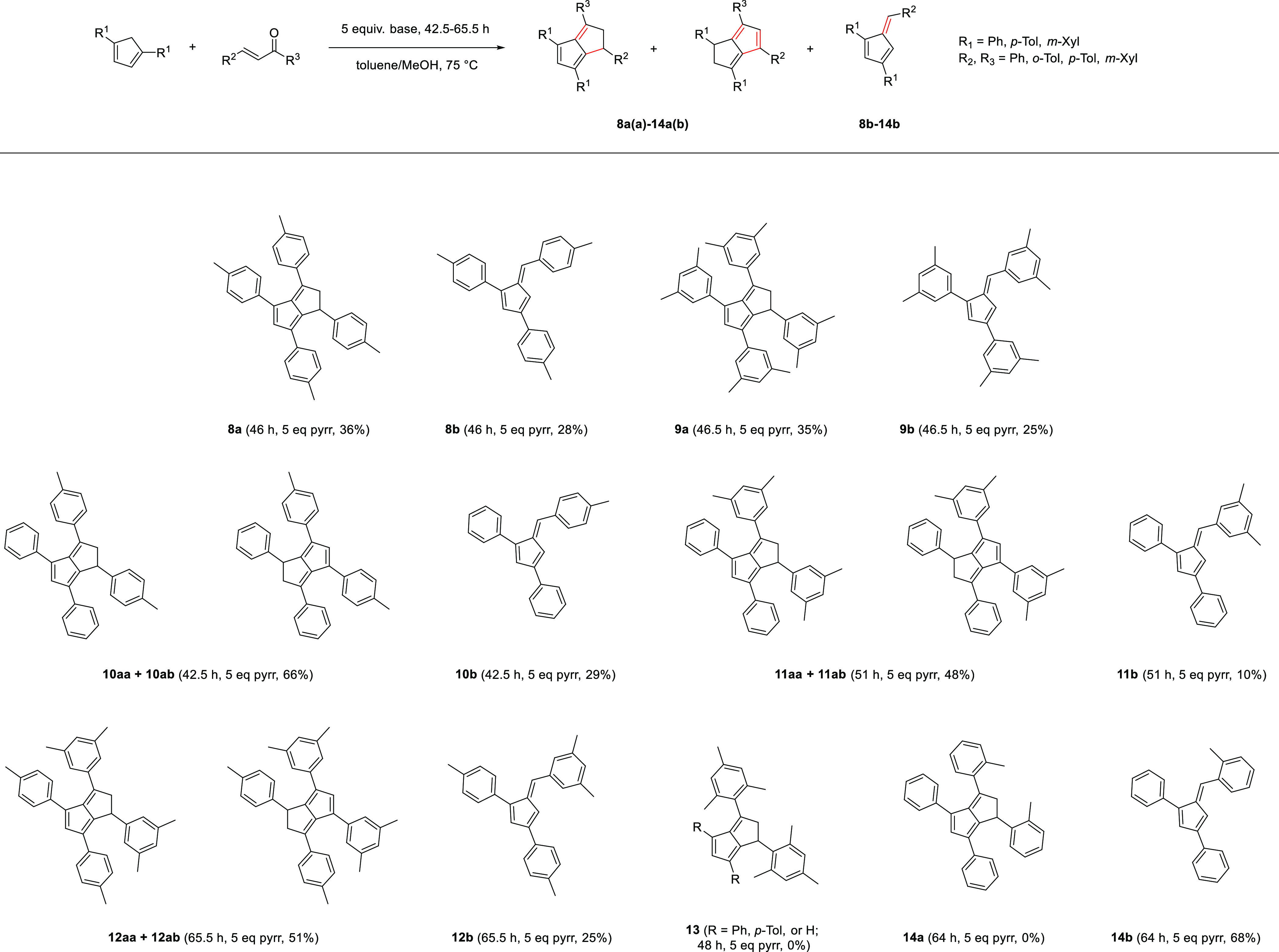
Scope and Limitation of Phenyl-, Tolyl-, Xylyl-, and Mesityl-Substituted Dihydropentalene and Pentafulvene Synthesis from Cyclopentadienes and Enones

**Scheme 7:**
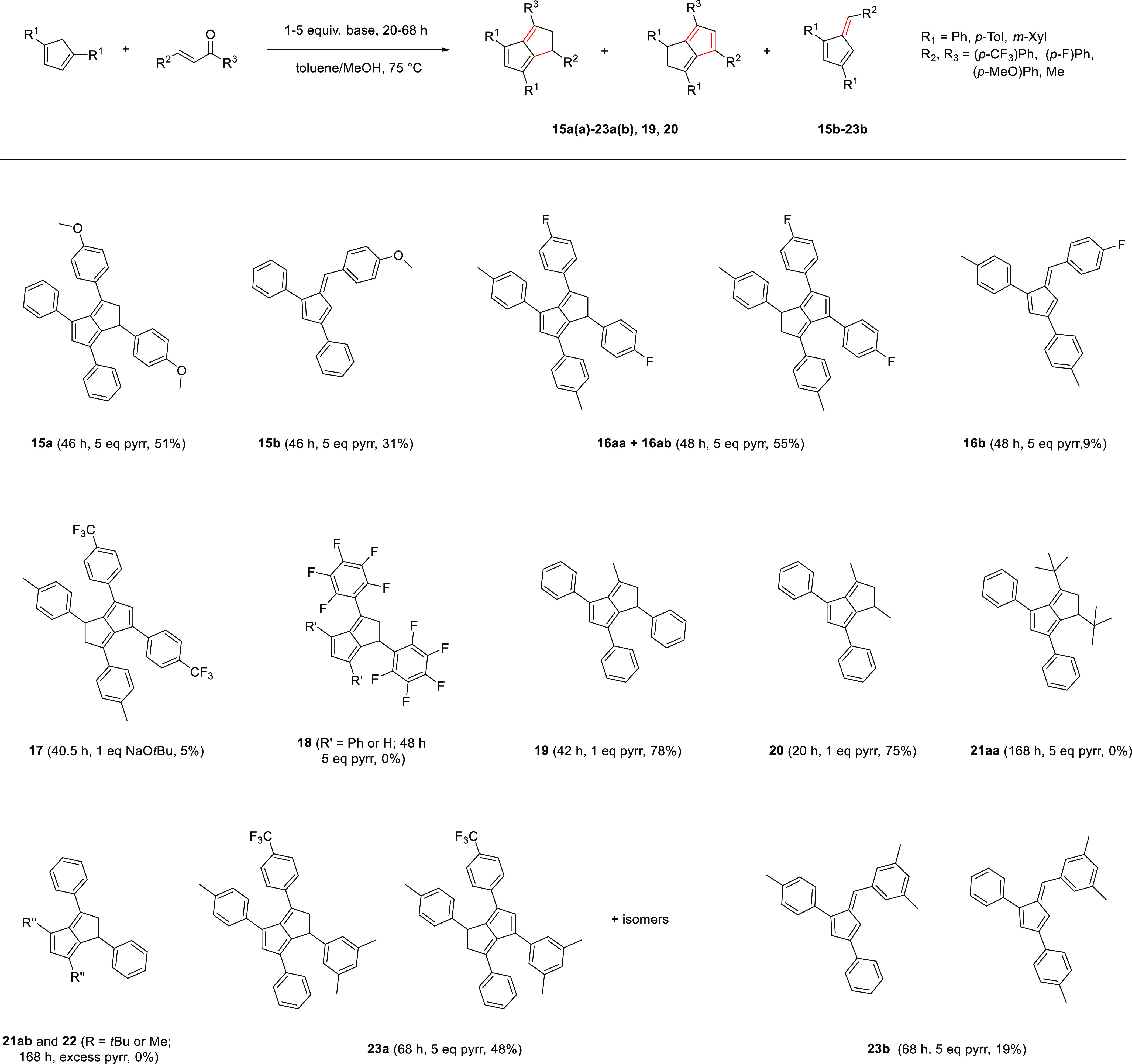
Scope and Limitations of Dihydropentalene As Well As Pentafulvene Synthesis With Oxygenated, Fluorinated, and Methyl Substituents from Cyclopentadienes and Enones

After the successful installation of *p*-tolyl and *m*-xylyl substituents, we evaluated the possibility of introducing a wider variety of substituents into **PnH_2_** via the cyclizing condensation reaction between substituted **CpH**s and enones (Schemes 6 and 7).

Trying to introduce bulky mesityl substituents into a dihydropentalene via the enone component, **Ph_2_CpH** and ***p*-Tol_2_CpH** were reacted with 1,3-dimesitylprop-2-en-1-one. However, as with the attempted synthesis of **Mes_2_CpH** (see Section 2.1), no reaction occurred after 48 h. Using a stoichiometric amount of KO*t*Bu in THF instead of pyrrolidine in MeOH/toluene did also not lead to any conversion after several days at 75 °C. Even when attempting to react unsubstituted **CpH** with 1,3-dimesitylpropenone under the same conditions, the bulky enone was left untouched as confirmed by NMR spectroscopy (**13**). Reducing the steric demand of the enone to 1,3-di-*o*-tolylprop-2-en-1-one (a regio-isomer of the successful 1,3-di-*p*-tolyl enone; see **8a**, **10a**, and **12a**) did show a color change after a few hours in solution with **Ph_2_CpH** and 5 equiv of pyrrolidine. However, no **(*o*Tol)_2_Ph_2_PnH_2_** could be isolated from this reaction, but 68% of 1,3-diphenyl-6-*o*-tolylpentafulvene (**14b**) was obtained instead (Scheme 6). This result showed steric hindrance in the *ortho* position of aryl substituents to be a limitation in this cyclizing condensation reaction (either on the nucleophile or the electrophile), with one *o*-methyl group on each aromatic substituent favoring pentafulvene formation and two *o*-methyl groups shutting down reactivity under the conditions applied (see pentafulvene formation discussion in Section 2.2).

Since enones containing methylated aryl groups successfully reacted with **1,3-R_2_CpH** to furnish dihydropentalenes, we investigated the introduction of aryl substituents containing more electron-donating groups. When **Ph_2_CpH** was reacted with 1,3-bis(4-methoxy-phenyl)prop-2-en-1-one, the corresponding 1,3-diphenyl-6-(4-methoxy-phenyl)pentafulvene (**15b**) was obtained as a dark red solid in 31% yield alongside 1,3-bis(4-methoxyphenyl)-4,6-diphenyl-1,2-dihydropentalene (**15a**) as cherry-red solid in 51% yield. The observation that **15a** was formed as a single isomer (as confirmed by HMBC) suggested that an electron-rich aryl group impedes the isomerization into the corresponding 1”,2” double bond isomer under reaction conditions (Scheme 7). The isolation of a single isomer of an unsymmetrically substituted dihydropentalene allowed UV–vis spectroscopic comparison with some symmetrically tetrasubstituted congeners (Figure S62). The substitution of two phenyl groups with two 4-methoxyphenyl substituents caused a 35% lowering in molar absorptivity at 286 nm but largely unchanged intensity around 350 nm, where broadening and a slight redshift were noticeable in **15a** compared to **Ph_4_PnH_2_**. This effect was even more pronounced in the corresponding pentafulvenes where the introduction of 4-methoxyphenyl gave a much weaker chromophore (**15b** displaying less than half the molar extinction coefficient of **Ph_3_Fv**; see Figures S59 and 62).

We then tested the scope of the synthetic protocol with electron-withdrawing groups on the aryl substituents of the enone using a slightly more electron-rich cyclopentadiene to furnish push–pull bicyclic systems (Scheme 7). When ***p*-Tol_2_CpH** was reacted with 1,3-bis(4-fluorophenyl)prop-2-en-1-one, we isolated a small amount of the corresponding 1,3-ditolyl-6-(4-fluoro-phenyl)pentafulvene (**16b**) in 9% yield and a mixture of 1,3-bis(4-fluorophenyl)-4,6-di-p-tolyl-1,2-dihydro-pentalene (**16aa**) as well 4,6-bis(4-fluorophenyl)-1,3-di-p-tolyl-1,2-dihydropentalene (**16ab**) in 55% combined yield, with a **16aa**/**16ab** isomer ratio of approximately 1:1 (see Figures S39–S42). The relatively low amount of pentafulvene formation suggested a decreased propensity for retro-aldol cleavage (pathway 2, Scheme 4) caused by electron-withdrawing substituents such as a fluorine atom in the *para*-position on the arylated enone. Leaving **16aa**/**16ab** in CDCl_3_ for 21 days at room temperature resulted in slow decomposition as well as a minor change in the isomer ratio from 10:9 to 10:14, meaning the introduction of one fluorine atom in the *para-*position led to a reduction of the kinetic isomerization barrier. Investigating this effect further through the use of a more strongly electron-withdrawing CF_3_ group in 1,3-bis(4-(trifluoromethyl)phenyl)prop-2-en-1-one, we found its reaction with ***p*-Tol_2_CpH** to be challenging. While the formation of a single dihydropentalene isomer 1,3-di-*p*-tolyl-4,6-bis(4-(trifluoromethyl)phenyl)-1,2-dihydropentalene **17** in the crude reaction mixture was firmly established by NMR spectroscopy, it could only be isolated in 31% yield at 88% purity. All attempts to remove unreacted **5** remaining in the sample led to very low yields at only marginally improved purity. Higher temperatures, extended reaction times, or using an excess of enone added in portions over time produced much lower amounts of **17**, implying that significant side reactions occurred with this reactive enone under these conditions. Applying gentler reaction conditions, **5** was preactivated by deprotonation with NaO*t*Bu in THF, to which a dilute solution of the enone was slowly added dropwise at room temperature (causing an immediate color change), stirred for 19.5 h at room temperature, and then heated to 75 °C for 21 h (see Section 1). Analyzing the crude after a biphasic work up showed no remaining **5**, and liquid chromatography followed by recrystallization gave pure **17** as a cherry-red air-stable solid in 5% yield. No pentafulvene formation was observed in this reaction, confirming again the ionic NaO*t*Bu route to reduce pentafulvene formation and that electron-withdrawing substituents on the enone activate it for dihydropentalene formation over retro-aldol cleavage—both factors leading to the requirement for milder reaction conditions in these cases.

Curious about the limits of enone activation through electron-withdrawing substituents, we employed 1,3-bis(perfluorophenyl)prop-2-en-1-one as the electrophile with **Ph_2_CpH** as well as **CpH**. Using either excess pyrrolidine in refluxing MeOH/toluene or NaOtBu in THF at room temperature, no formation of the desired dihydropentalenes **18** occurred, with NMR analyses only showing degradation of the enone to unidentifiable mixtures. Thus, in addition to steric limits encountered with *ortho*-aryl substituents on either reagent encountered in **13** and **14a**, the electron-withdrawing nature of the enone substituents seems to influence the degree of double bond isomerization in the dihydropentalene product, govern the amount of pentafulvene formation during the reaction, and, if too activated, lead to based-induced decomposition rather than reactivity toward the **CpH** Michael donor (Scheme 7).

We also investigated the possibility of introducing non-aryl substituents (Scheme 7), by reacting **Ph_2_CpH** with 4-phenylbut-3-en-2-one, an enone which has previously been reported to display the desired reactivity with **CpH** to furnish **3-Me-1-PhPnH_2_**.^10^ With one equivalent of pyrroline in a 1:1 mixture of methanol and toluene at 70 °C, this reaction yielded **3-Me-1,4,6-Ph_3_PnH_2_** (**19**) after 42 h in 78% yield. Interestingly, it formed exclusively as one 1,2-isomer that did not rearrange into the 1,5-isomer as reported for **3-Me-1-PhPnH_2_**,^10^ even after prolonged heating to 155 °C or treating with silica or acidic alumina. Moving to two methyl substituents, we explored the use of pent-3-en-2-one with **Ph_2_CpH**, an enone that is known to undergo a 1,2-addition with **CpH** and pyrrolidine in methanol instead of the desired 1,4-addition.^26^ Surprisingly, the pyrrolidine-facilitated reaction of **Ph_2_CpH** with pent-3-en-2-one gave **1,3-Me_2_-4,6-Ph_2_PnH_2_** (**20**) in 75% yield even when using commercial pent-3-en-2-one of 85% purity. Pure samples of **20** were obtained by stripping through silica followed by recrystallization from ethanol. **20** had a melting point of 122–123 °C, 29 °C lower than **19** presumably due to further reduced π stacking. Trying to introduce more sterically demanding and electron-donating *tert*-butyl groups via the electrophile, we attempted the reaction of 2,2,6,6-tetramethylhept-4-en-3-one and **Ph_2_CpH** with pyrrolidine in MeOH/toluene. No reaction was observed even after refluxing for 1 week, with NMR investigations showing both reagents to remain unchanged. The inertness of this enone was further confirmed by using **CpH** as the pro-nucleophile, which also failed to react under these conditions, suggesting a combination of steric and electronic factors on the enone to inhibit the 1,4-attack. Trying to access a potential ***t*Bu_2_Ph_2_PnH_2_** (**21aa and 21ab**) via a different route, we inverted the substitution pattern of the pro-nucleophile and the electrophile. A freshly prepared di-*tert*-butylcyclopentadiene 1,4/1,3 isomer mixture (***t*Bu_2_CpH**)^31^ was reacted with chalcone using 5 equiv pyrrolidine in MeOH/toluene and also using neat pyrrolidine as the solvent. After 7 days at 75 °C, only decomposed chalcone was detected in these reactions. Haberland et al. recently demonstrated that a large excess of NaOMe is able to activate ***t*Bu_2_CpH** toward pentafulvene formation.^32^ We replicated those conditions in the condensation of ***t*Bu_2_CpH** with chalcone, as well as in the condensation of a freshly prepared dimethylcyclopentadiene mixture (**Me_2_CpH**)^33^ with chalcone, with the aim of synthesizing **Ph_2_Me_2_PnH_2_** (**22**), the 1”,2”-isomer of **20**. Both cases showed reactivity but led to a complex product mixture in each case, with recrystallization or liquid chromatography of the corresponding crude mixtures being unsuccessful. Since we previously demonstrated a successful [6 + 2] condensation between **Ph_3_Fv** and acetophenone with pyrrolidine to give **Ph_4_PnH_2_**,^16^ we attempted the [6 + 2] condensation between 1,3-di^*t*^butyl-6-phenylpentafulvene (**6-Ph-*t*Bu_2_Fv**)^32^ and acetophenone in the presence of pyrrolidine. However, even after 7 days at 75 °C in MeOH/toluene or MeCN, the pentafulvene showed no reaction. We thus conclude that other approaches are needed to access pure **21aa**, **21ab,** and **22**, and complementary methods to access these types of molecules will be reported in due course.

Having explored the limits of the cyclizing condensation method, we investigated the possibility of synthesizing a dihydropentalene with four different substituents **R_1_R_2_R_3_R_4_PnH_2_** (Scheme 7). After treating a **CpH** with a phenyl group in 1-position and tolyl group in 4-position (**7**) with pyrrolidine in the presence of 3-(3,5-dimethylphenyl)-1-(4-(trifluoromethyl)phenyl)prop-2-en-1-one at 75 °C for 68 h, two major fractions were obtained from silica-based liquid chromatography. The first dark orange-red fraction contained a regio-isomeric mixture of pentafulvenes (1-(phenyl)-3-(*p*-tolyl)-6-(3,5-dimethyl-phenyl)fulvene and 1-(*p*-tolyl)-3-(phenyl)-6-(3,5-dimethylphenyl)fulvene; summarized as **23b**) in a combined yield of 19%. The second dark violet-red fraction contained a mixture of at least seven different isomers of **(Ph)(*p*-Tol)(*m*-Xyl)(*p*-CF_3_Ph)PnH_2_** (summarized as **23a**) according to NMR spectroscopic analysis (confirmed by ^19^F NMR and DOSY as well as mass spectrometry) in a combined yield of 25%. Thus, although not all of these isomers could be fully assigned or isolated in pure form, this result demonstrates that it is possible to introduce four different substituents into a dihydropentalene framework using our method. Collective double deprotonation of this mixture would yield two pentalenide species as reported for isomeric mixtures of the unsubstituted dihydropentalenes.^4,34^

## 3 Conclusions

We have shown that the base-promoted and transition metal-free synthesis of 1,3,4,6-tetraarylated 1,2-dihydropentalenes via Michael addition of a cyclopentadiene to an enone followed by an intramolecular condensation tolerates a range of substitution patterns on both the nucleophile and the electrophile. A variety of novel tetraarylated dihydropentalenes may be accessed via either soft activation with a weak base at high temperature (pyrrolidine in refluxing MeOH/toluene) or hard activation at low temperature (NaO*t*Bu at room temperature in THF) in moderate to good yields from easily available and inexpensive starting materials. The latter method typically gives lower product yields but at higher dihydropentalene selectivity with lower amounts of pentafulvene formation. All dihydropentalenes were isolated as air-stable solids with lower melting points and higher solubility compared to the symmetrically substituted tetraphenyl-dihydropentalene.^16^ The products consisted exclusively of the fulvenic 1,2 isomers with no sign of 1,4 or 1,5 double bond isomer formation. With different substituents on each ring, there existed two different 1,2 regio-isomers that interconverted when heated in the presence of base if the polarization of the product favored conjugation opposite to that obtained from the initial cyclization, generally favoring two double bonds in the five-membered ring with the more electron-withdrawing substituents. Methylated aromatic substituents only isomerized above 60 °C, whereas electron-withdrawing aromatic substituents gave slow isomerization at room temperature, with 4-trifluoromethylphenyl substitution leading to complete inversion of the double bond position. Retro-aldol cleavage of the Michael intermediate may produce a triarylated pentafulvene side product when using arylated enones with electron-donating substituents and high reaction temperatures. These valuable compounds typically synthesized from air-sensitive aldehydes^24,25^ can be cleanly isolated from the reaction mixture and used for separate cyclization to dihydropentalenes or other applications in organic and organometallic chemistry.^35^ The more electron-poor the Michael acceptor, the lower the amount of pentafulvene formation, with *p*-trifluoromethylbenzene substitution on the enone producing exclusively dihydropentalene but pentafluorobenzene-substituted enone being too reactive to give either. Steric limits in the initial 1,4 addition and **PnH_2_** formation were encountered with aryl substituents in the *ortho* position and bulky ^*tert*^butyl groups. To demonstrate versatility, we have synthesized the first dihydropentalene with four different substituents as mixture of regio-isomers. The novel dihydropentalenes reported may find use as starting materials for polyquinanes,^1,3^ precursors for the synthesis of ansa-metallocenes for olefin polymerizations^36^ and organometallic pentalenide chemistry with a variety of p-, d-, and f-block metals.^7^

## 4 Experimental Section

### 4.1 General

Commercially available materials were obtained from Fluorochem, Sigma Aldrich, Alfa Aesar, Fisher, or Acros. All manipulations were carried out under dry argon using standard Schlenk techniques or using an MBraun Unilab Plus glovebox, unless specified otherwise. Pre-coated TLC sheets from Machery-Nagel (ALUGRAM SIL G/UV_254_, silica gel 60 with fluorescent indicator UV_254_) were used for thin layer chromatography. Preparative thin layer chromatography was performed on silica plates (ColePalmer Uniplate, silica gel GF, UV254, 20 × 20 cm, 2000 μm), and silica gel (Sigma-Aldrich, 60 Å, 200–400 mesh particle size) was used for preparative liquid flash chromatography.

### 4.2 Analysis

NMR spectroscopy was conducted using a 400 or 500 MHz instrument at 25 °C. Chemical shifts (δ) are reported in ppm relative to the residual proton chemical shifts of the deuterated solvent used (^1^H and ^13^C{^1^H}) or relative to external standards (BF_3_·Et_2_O for ^19^F{^1^H}). Mass spectrometry (Agilent 6545 QTOF or Bruker MaXis HD ESI-QTOF) was carried out at the Material and Chemical Characterization Facility at the University of Bath. Melting points were determined using an Electrothermal IA9300 Digital Melting Point Apparatus. UV–vis spectroscopy was conducted using an Avantes AvaLight-DH-S-BAL light source with an AvaSpec-2048 L photospectrometer using 400 μm fiber-optic cables.

### 4.3 Solvents

Methanol was dried by distillation from magnesium. Toluene was dried by distillation from sodium. THF was dried by distillation from potassium. CDCl_3_ and other solvents were used without purification.

### 4.4 Reagents

Precursors for substituted cyclopentadienes were synthesized according to literature.^18,37^ Non-commercial but previously reported α-β unsaturated ketones were synthesized according to literature.^38−42^ Pyrrolidine was purified by distillation under argon.

### 4.5 Synthesis of Starting Materials

#### 4.5.1 Ester Synthesis

To a solution of carboxylic acid (56 mmol, 1 equiv) in dry ethanol (50 mL, 856 mmol, 15.3 equiv), concentrated H_2_SO_4_ (2.5 mL) was added and refluxed for 15–23 h in an oil bath. After cooling to room temperature, ethanol was removed under reduced pressure and the residue was dissolved in diethyl ether (80 mL). The organic solution was washed with aqueous NaHCO_3_ (3 × 70 mL), water (70 mL), and dried over MgSO_4_. After evaporation of solvent under reduced pressure, the esters **1–3** were obtained in sufficient purity for use in subsequent transformations.

##### 4.5.1.1 Ethyl-4-oxo-4-phenylbutanoate (**1**)

Orange oil (10.09 g, 48.9 mmol, 87%). Reaction time 15 h. ^1^H NMR (400 MHz, CDCl_3_): δ = 7.98 (d,^3^*J*_HH_ = 7.67 Hz, 2H), 7.56 (d,^3^*J*_HH_ = 7.31 Hz, 1H), 7.45 (t,^3^*J*_HH_ = 7.46 Hz, 2H), 4.16 (q,^3^*J*_HH_ = 7.07 Hz, 2H,), 3.30 (t,^3^*J*_HH_ = 6.48 Hz, 2H), 2.75 (t,^3^*J*_HH_ = 6.48 Hz, 2H), 1.26 (t,^3^*J*_HH_ = 7.12 Hz, 3H)—as previously reported.^43^

##### 4.5.1.2 Ethyl-4-oxo-4-(p-tolyl)butanoate (**2**)

Colorless crystalline solid (8.41 g, 38.2 mmol, 92% based on 41.6 mmol acid). Reaction time 15 h. ^1^H NMR (500 MHz, CDCl_3_): = 7.81 (d,^3^*J*_HH_ = 8.17 Hz, 2H), 7.19 (d,^3^*J*_HH_ = 8.17 Hz, 2H), 4.16 (q, ^3^*J*_HH_ = 7.14 Hz, 2H), 3.22 (t,^3^*J*_HH_ = 6.70 Hz, 2H), 2.67 (t,^3^*J*_HH_ = 6.70 Hz, 2H), 2.34 (s, 3H), 1.19 (t,^3^*J*_HH_ = 7.14 Hz, 3H)—as previously reported.^44^

##### 4.5.1.3 Ethyl-4-(3,5-dimethylphenyl)-4-oxobutanoate (**3**)

Colorless crystalline solid (516 mg, 2.21 mmol, 96% based on 2.37 mmol acid). Reaction time 23 h. Melting point 74 °C. ^1^H NMR (500 MHz, CDCl_3_): δ = 7.59 (s, 2H), 7.20 (s, 1H), 4.15 (q,^3^*J*_HH_ = 7.11 Hz, 2H), 3.28 (t,^3^*J*_HH_ = 6.69 Hz, 2H), 2.74 (t,^3^*J*_HH_ = 6.69 Hz, 2H), 2.36 (s, 6H) 1.26 (t,^3^*J*_HH_ = 7.11 Hz, 3H). ^13^C{^1^H} NMR (125 MHz, CDCl_3_): δ = 198.7, 173.1, 138.3, 136.8, 134.9, 126.0, 60.7, 33.6, 28.5, 21.4, 14.3. HRMS (ESI) *m/z*: [M + Na]^+^ Calculated for C_14_H_18_O_3_Na 257.1148; Found 257.1152.

#### 4.5.2 Arylated Cyclopentadienes

Using a modification of the method reported by Drake and Adams^17^ as well as by Clennan and Mehrsheikh-Mohammadi,^18^ the ethyl ester (38 mmol, 1 equiv) in dry toluene (20 mL) was added dropwise to sodium ethoxide (80 mmol, 2.1 equiv) in dry toluene (80 mL) at 0 °C. After warming to room temperature, the corresponding acetophenone (38 mmol, 1 equiv) was added to the red solution and it was stirred at 45 °C for 72 h in an oil bath. After cooling the solution to 0 °C, water (200 mL) was slowly added and stirred at room temperature for 1 h. Diethyl ether (200 mL) was added, and the aqueous fraction was collected and heated to 70 °C for 2 h. The resulting yellow solid was filtered, washed with water (2 × 50 mL), and recrystallized from ethanol to give **4**–**7**.

##### 4.5.2.1 1,4-Diphenylcyclopenta-1,3-diene (**4**)

Golden yellow crystalline solid (3.55 g, 16.3 mmol, 47% from 35 mmol ester). ^1^H NMR (500 MHz, CDCl_3_): δ = 7.59–7.55 (m, 4H), 7.38–7.32 (m, 4H), 7.29–7.24 (m, 2H), 6.95 (s, 2H), 3.80 (s, 2H)—as previously reported.^45^

##### 4.5.2.2 1,4-Di-p-tolylcyclopenta-1,3-diene (**5**)

Golden yellow crystalline solid (2.34 g, 9.50 mmol, 25% from 38 mmol ester). ^1^H NMR (400 MHz, CDCl_3_): δ = 7.49 (d,^3^*J*_HH_ = 7.6 Hz, 4H), 7.19 (d,^3^*J*_HH_ = 7.6 Hz, 4H), 7.13 (t,^3^*J*_HH_ = 7.3 Hz, 2H), 6.91 (s, 2H), 3.78 (s, 2H), 2.39 (s, 6H)—as previously reported.^18^

##### 4.5.2.3 1,4-Bis(3,5-dimethylphenyl)cyclopenta-1,3-diene (Major Isomer) and 1,3-Bis(3,5-dimethylphenyl)-cyclopenta-1,3-diene (Isomer Ratio 10:3 Major:Minor) (**6**)

Yellow specular solid (111 mg, 0.41 mmol, 18% from 2.21 mmol ester). Melting point 151 °C. ^1^H NMR (500 MHz, CDCl_3_): δ = 7.26 (s, 2H, minor isomer), 7.25 (s, 1H, minor isomer), 7.22 (s, 2H, minor isomer), 7.20 (s, 4H, major isomer), 6.95 (s, 1H, minor isomer), 6.91 (s, 2H, major isomer), 6.89 (s, 1H, minor isomer), 6.87 (s, 2H, major isomer), 6.60 (s, 2H, minor isomer), 3.76 (s, 2H, major isomer), 3.56 (s, 2H, minor isomer), 2.37 (s, 6H, minor isomer), 2.34 (s, 6H, minor isomer), 2.34 (s, 12H, major isomer). ^13^C{^1^H} NMR (125 MHz, CDCl_3_): δ = 147.8, 146.8, 145.8, 138.1, 136.2, 136.1, 135.7, 129.2, 128.8, 128.6, 128.1, 127.0, 125.8, 123.9, 123.1, 122.9, 41.9, 41.1, 21.5. HRMS (ESI) *m/z*: [M + H]^+^ Calculated for C_21_H_23_ 275.1794; Found 275.1792.

##### 4.5.2.4 1-Phenyl-4-*p*-tolylcyclopenta-1,3-diene (**7**)

Yellow crystalline solid (0.52 g, 2.24 mmol, 30% from 7.5 mmol ester). ^1^H NMR (400 MHz, CDCl_3_): δ = 7.56 (d,^3^*J*_HH_ = 7.7 Hz, 2H), 7.47 (d,^3^*J*_HH_ = 8.1 Hz, 2H), 7.34 (t,^3^*J*_HH_ = 7.7 Hz, 2H), 7.20 (t,^3^*J*_HH_ = 7.7 Hz, 1H), 7.16 (d,^3^*J*_HH_ = 8.1 Hz, 2H), 6.94 (s, 1H), 6.89 (s, 1H), 3.77 (s, 2H), 2.36 (s, 3H). ^13^C{^1^H} NMR (125 MHz, CDCl_3_): δ = 145.9, 145.2, 136.7, 136.2, 133.4, 129.5, 128.8, 128.4, 127.4, 126.8, 124.9, 41.0, 21.3. HRMS (ESI) *m/z*: [M + H]^+^ Calculated for C_18_H_17_ 233.1325; Found 233.1324.

#### 4.5.3 Alpha-Beta-Unsaturated Ketones

##### 4.5.3.1 (E)-1,3-Bis(3,5-dimethylphenyl)prop-2-en-1-one

Using a modified synthesis method described by Volz and Hassler,^27^ 3,5-dimethylbenzaldehyde (1.10 g, 8.22 mmol) and 1-(3,5-dimethyl-phenyl)ethan-1-one (1.27 g, 8.59 mmol) were dissolved in laboratory grade (wet) methanol in air with stirring. At room temperature, KOH (263 mg, 3.99 mmol; in 4 mL methanol) was added dropwise over 3 min, followed by stirring the solution for 4.5 h at 80 °C in an oil bath, during which a precipitate formed. After cooling down to room temperature, the precipitate was separated via decanting and the white solid was washed with water (20 mL), followed by filtration. The solid was washed another two times with water (20 mL each) and dried in vacuo, resulting in a white solid (1.34 g, 5.09 mmol, 62% yield). Melting point 96 °C. ^1^H NMR (500 MHz, CDCl_3_): δ = 7.74 (d,^3^*J*_HH_ = 15.8 Hz, 1H), 7.63 (s, 2H), 7.48 (d,^3^*J*_HH_ = 15.8 Hz, 1H), 7.27 (s, 2H), 7.22 (s, 1H), 7.06 (s, 1H), 2.41 (s, 6H,), 2.36 (s, 6H). ^13^C{^1^H} NMR (125 MHz, CDCl_3_): δ = 191.1, 145.0, 138.60, 138.58, 138.4, 135.0, 134.5, 132.4, 126.5, 126.4, 122.1, 21.4, 21.4. HRMS (ESI) *m/z*: [M + H]^+^ Calculated for C_19_H_21_O 265.1587; Found 265.1591.

##### 4.5.3.2 (E)-3-(3,5-Dimethylphenyl)-1-(4-(trifluoromethyl)phenyl)prop-2-en-1-one

Using a modified synthesis method described by Tok and Kocyigit-Kaymakcioglu,^46^ 4-(trifluoromethyl)acetophenone (486 mg, 2.58 mmol) and 3,5-dimethylbenzaldehyde (427 mg, 3.19 mmol) were dissolved in laboratory grade (wet) methanol (10 mL) in air with stirring at 20 °C, and then NaOH (10% aqueous solution, 2.50 mmol) was added to the reaction mixture dropwise over 1 min. After 5 min, another portion of ketone was added (90 mg, 0.48 mmol). The reaction flask was sealed, and the mixture was stirred at room temperature for 18 h. The resulting precipitate was filtered and washed with ice-cold water (40 mL), followed by recrystallization from boiling methanol (10 mL) to give yellow needles (513 mg, 1.69 mmol, 57% yield). Melting point 102–103 °C. ^1^H NMR (500 MHz, CDCl_3_): δ = 8.10 (d,^3^*J*_HH_ = 8.1 Hz, 2H), 7.78 (d,^3^*J*_HH_ = 15.7 Hz, 1H), 7.77 (d,^3^*J*_HH_ = 8.1 Hz, 2H), 7.45 (d,^3^*J*_HH_ = 15.7 Hz, 1H), 7.27 (s, 2H), 7.09 (s, 1H), 2.37 (s, 6H). ^13^C{^1^H} NMR (125 MHz, CDCl_3_): δ = 189.9, 146.7, 141.3, 138.8, 134.6, 134.1 (q,^2^*J*_CF_ = 32.2 Hz), 133.0, 128.9, 126.6, 125.8 (q,^3^*J*_CF_ = 3.6 Hz), 125.6 (via ^1^H-^13^C-HMBC), 121.4, 21.4. ^19^F{^1^H} NMR (470 MHz, CDCl_3_): δ = −63.0 ppm. HRMS (ESI) *m/z*: [M + H]^+^ Calculated for C_18_H_16_F_3_O 305.1148; Found 305.1147.

#### 4.5.4 Typical Procedure for the Synthesis of Dihydropentalenes and Pentafulvenes

Using a modified synthesis method described in our previous work,^16^ the arylated cyclopentadiene (0.4 mmol, 1 equiv) and alpha-beta-unsaturated ketone (0.6 mmol, 1.5 equiv) were dissolved in 10 mL of dry methanol plus 10 mL of dry toluene under stirring at room temperature in a Schlenk flask. Pyrrolidine (2 mmol, 5 equiv) was added dropwise over 10 min, the reaction vessel was sealed, and the resulting solution was stirred for 20–64 h at 75 °C in an oil bath. After cooling to room temperature, to the dark red solution was added acetic acid (0.2 mL) in air and the solution was stirred for 5 min. The solvent was removed under reduced pressure, and the crude material was dissolved in diethyl ether (20 mL) as well as aqueous Na_2_CO_3_ (20 mL). The organic phase was washed with water (2 × 20 mL) and brine (20 mL). The solvent of the ether fraction was removed under reduced pressure, and the crude dissolved in a minimum of 1:1 diethyl ether/*n*-hexane, followed by drying and filtering through neutral silica using 2:1 *n*-hexane/diethyl ether as the eluent, collecting the first dark red-violet band only. This fraction was further purified, depending on scale either via preparative thin layer chromatography (cyclohexane/toluene systems as eluent) or via preparative liquid flash chromatography (cyclohexane/toluene systems as eluent). The dark orange first band gave the corresponding pentafulvenes **8b–23b,** and the dark purple second band gave the corresponding dihydropentalenes **8aa–23a** and **8ab–23a**. Instead of preparative thin layer chromatography, dihydropentalenes **19** and **20** were recrystallized from boiling ethanol after filtration over silica. Crystalline samples may be obtained by a final recrystallization from boiling methanol.

##### 4.5.4.1 1,3,4,6-Tetra-p-tolyl-1,2-dihydropentalene (**8a**)

Cherry-red solid (66 mg, 0.14 mmol, 36% based on 0.4 mmol **5**). Melting point 96–97 °C. Reaction time 46 h, *R*_f_ = 0.42 (5:1 cyclohexane/toluene). ^1^H NMR (400 MHz, CDCl_3_): δ = 7.36–7.31 (m, 3H), 7.24–7.16 (m, 6H), 7.10–6.94 (m, 8H), 4.56 (d,^3^*J*_HH_ = 6.7 Hz, 1H), 4.10 (dd,^3^*J*_HH_ = 6.7 Hz,^2^*J*_HH_ = 18.7 Hz, 1H), 3.42 (d,^2^*J*_HH_ = 18.7 Hz, 1H), 2.36 (s, 3H), 2.33 (s, 3H), 2.32 (s, 3H), 2.29 (s, 3H). ^13^C{^1^H} NMR (125 MHz, CDCl_3_): δ = 154.0, 147.7, 146.0, 141.7, 139.7, 138.9, 135.8, 135.7, 134.1, 132.6, 132.5, 130.3, 130.2, 129.7, 129.5, 129.2, 129.1, 128.7, 128.6, 128.3, 127.3, 126.4, 55.4, 42.3, 21.5, 21.3, 21.3, 21.2. HRMS (ESI) *m/z*: [M + H]^+^ Calculated for C_36_H_33_ 465.2577; Found 465.2573.

##### 4.5.4.2 1,3,6-Tri-p-tolylpentafulvene (**8b**)

Dark orange-red solid (38 mg, 0.11 mmol, 28% yield based on 0.4 mmol **5**). Melting point 105 °C. Reaction time 46 h, *R*_f_ = 0.63 (5:1 cyclohexane/toluene). ^1^H NMR (500 MHz, CDCl_3_): δ = 7.62 (d,^3^*J*_HH_ = 7.9 Hz, 2H), 7.56 (d,^3^*J*_HH_ = 7.9 Hz, 2H), 7.40–7.37 (m, 2H), 7.29–7.20 (m, 7H), 7.03 (s, 1H), 6.96–6.95 (m, 1H), 2.44, 2.43, 2.39 (all s, 9H). ^13^C{^1^H} NMR (125 MHz, CDCl_3_): δ = 146.6, 143.8, 141.8, 139.5, 138.2, 137.9, 136.8, 134.6, 133.4, 132.7, 130.8, 129.6, 129.5, 129.4, 129.2, 127.5, 126.2, 113.7, 21.6, 24.5, 21.4. HRMS (ESI) *m/z*: [M + H]^+^ Calculated for C_27_H_25_ 349.1951; Found 349.1948.

##### 4.5.4.3 1,3,4,6-Tetrakis(3,5-dimethylphenyl)-1,2-dihydropentalene (**9a**)

Cherry-red solid (13 mg, 2.5 × 10^–5^ mol, 35% based on 7.1 × 10^–5^ mol **6**). Melting point 95–96 °C. Reaction time 46.5 h, *R*_f_ = 0.25 (10:1 cyclohexane/toluene). ^1^H NMR (500 MHz, CDCl_3_): δ = 7.30 (s, 1H), 7.02 (s, 2H), 6.94–6.91 (m, 5H), 6.86 (s, 2H), 6.81 (s, 2H), 6.74 (s, 1H), 4.41 (d,^3^*J*_HH_ = 6.5 Hz, 1H), 4.04 (dd,^3^*J*_HH_ = 6.5 Hz,^2^*J*_HH_ = 19 Hz, 1H), 3.48 (d,^2^*J*_HH_ = 19 Hz, 1H), 2.26, 2.19, 2.15, 2.09 (all s, 24H). ^13^C{^1^H} NMR (125 MHz, CDCl_3_): δ = 154.8, 149.1, 146.8, 145.3, 138.8, 138.1, 137.5, 137.2, 136.6, 135.3, 135.1, 131.0, 130.4, 129.7, 128.4, 128.2, 128.0, 127.7, 127.6, 126.6, 125.5, 124.6, 55.0, 42.9, 21.5, 21.4, 21.3, 21.1. HRMS (ESI) *m/z*: [M + Na]^+^ Calculated for C_40_H_40_Na 543.3022; Found 543.3011.

##### 4.5.4.4 1,3,6-Tris(3,5-dimethylphenyl)pentafulvene (**9b**)

Dark orange red solid (7 mg, 1.8 × 10^–5^ mol, 25% based on 7.1 × 10^–5^ mol **6**). Melting point 85–86 °C. Reaction time 46.5 h, *R*_f_ = 0.41 (10:1 cyclohexane/toluene). ^1^H NMR (400 MHz, CDCl_3_): δ = 7.32 (s, 2H, Ar-*H*), 7.26 (s, 2H), 7.22 (s, 1H), 7.09 (s, 2H), 7.03 (s, 1H), 7.00 (s, 1H), 6.98–6.93 (m, 3H), 2.39 (s, 12H), 2.36 (s, 6H). ^13^C{^1^H} NMR (100 MHz, CDCl_3_): δ = 146.8, 144.4, 141.8, 138.7, 138.4, 138.2, 138.0, 137.4, 136.2, 135.5, 130.9, 129.8, 128.8, 128.6, 128.1, 127.4, 124.2, 114.6, 21.54, 21.49. HRMS (ESI) *m/z*: [M + H]^+^ Calculated for C_30_H_31_ 391.2420; Found 391.2422.

##### 4.5.4.5 4,6-Diphenyl-1,3-di-p-tolyl-1,2-dihydropentalene (**10aa**) and 1,3-Diphenyl-4,6-di-p-tolyl-1,2-dihydro-pentalene (**10ab**)

Cherry-red solid (117 mg, 0.27 mmol, 66% based on 0.41 mmol **4**), reaction time 42.5 h, *R*_f_ = 0.28 (5:1 cyclohexane/toluene). ^1^H NMR (500 MHz, CDCl_3_): δ = 7.42–6.88 (m, 18H, both), 7.33 (s, 1H), 4.57–4.51 (overlapping d’s, 1H), 4.13–4.03 (overlapping dd’s, 1H), 3.43–3.36 (overlapping d’s, 1H), 2.29, 2.27, 2.26, 2.24 (all s, 6H). ^13^C{^1^H} NMR (125 MHz, CDCl_3_): δ = 154.8, 153.8, 148.9, 147.2, 146.8, 145.9, 144.6, 141.4, 139.9, 139.1, 139.0, 137.0, 135.8, 135.7, 135.4, 135.3, 133.8, 132.4, 130.5, 130.4, 130.3, 130.1, 129.7, 129.5, 129.4, 129.1, 128.8, 128.72, 128.65, 128.4, 128.34, 128.27, 128.2, 128.1, 127.9, 127.5, 127.3, 126.44, 126.39, 126.2, 126.1, 55.3, 42.7, 42.4, 21.5, 21.3, 21.2. ^1^H DOSY NMR (500 MHz, CDCl_3_): *D* = (6.02 ± 0.04) × 10^–10^ m^2^ s^–1^. HRMS (ESI) *m/z*: [M + H]^+^ Calculated for C_34_H_29_ 437.2264; Found 437.2252.

##### 4.5.4.6 1,3-Diphenyl-6-p-tolylpentafulvene (**10b**)

Red solid (39 mg, 0.12 mmol, 29% yield based on 0.41 mmol **4**), reaction time 42.5 h, *R*_f_ = 0.52 (5:1 cyclohexane/toluene). ^1^H-NMR (500 MHz, CDCl_3_): δ = 7.73–7.69 (m, 2H), 7.57–7.52 (m, 2H), 7.50–7.23 (overlapping m’s, 11H), 7.07 (s, 1H), 6.99 (s, 1H), 2.41 (s, 3H)—as previously reported.^25^

##### 4.5.4.7 1,3-Bis(3,5-dimethylphenyl)-4,6-diphenyl-1,2-dihydropentalene and 4,6-Bis(3,5-dimethylphenyl)-1,3-diphenyl-1,2-dihydropentalene (**11aa** + **11ab**)

Cherry-red solid (244 mg, 5.26 × 10^–4^ mol, 48% yield based on 1.10 mmol **4**), reaction time 49 h. *R*_f_ = 0.19 (20:1 cyclohexane/toluene). ^1^H NMR (400 MHz, CDCl_3_): δ = 7.44–7.40 (m, 2H), 7.35–7.24 (m, 11H), 7.23–7.15 (m, 9H), 7.14–7.08 (m, 2H), 7.00 (s, 1H), 6.90 (s, 4H), 6.88 (s, 1H), 6.83–6.73 (m, 4H), 4.55 (d,^3^*J*_HH_ = 6.7 Hz, 1H), 4.50 (d,^3^*J*_HH_ = 6.7 Hz, 1H), 4.15–4.04 (m, 2H), 3.5–3.42 (m, 2H), 2.25, 2.18, 2.11, 2.05 (all s, 24H). ^13^C{^1^H} NMR (100 MHz, CDCl_3_): δ = 155.0, 154.2, 148.8, 148.4, 146.5, 145.0, 144.5, 139.1, 139.0, 138.2, 137.6, 137.4, 137.3, 136.1, 135.7, 135.5, 135.1, 134.8, 131.3, 130.6, 130.3, 130.0, 129.8, 129.3, 128.8, 128.7, 128.33, 128.31, 128.1, 128.0, 127.8, 127.7, 127.6, 127.4, 126.6, 126.4, 126.3, 126.2, 126.0, 125.3, 124.6, 55.4, 54.9, 43.1, 42.7, 21.5, 21.4, 21.3, 21.1. ^1^H DOSY NMR (500 MHz, CDCl_3_): *D* = (7.19 ± 0.05) × 10^–10^ m^2^ s^–1^. HRMS (ESI) *m/z*: [M + H]^+^ Calculated for C_36_H_33_ 465.2577; Found 465.2564.

##### 4.5.4.8 1,3-Diphenyl-6-(3,5-dimethylphenyl)pentafulvene (**11b**)

Dark red solid (38 mg, 1.14 × 10^–4^ mol, 10% yield based on 1.10 mmol **4**). Melting point 94–95 °C. reaction time 49 h, *R*_f_ = 0.32 (20:1 cyclohexane/toluene). ^1^H NMR (400 MHz, CDCl_3_): δ = 7.77–7.73 (m, 2H), 7.54–7.38 (m, 7H), 7.37–7.32 (m, 1H), 7.30 (s, 2H), 7.28 (s, 1H), 7.11–7.09 (m, 1H), 7.08–7.06 (s, 1H), 7.04–7.02 (m, 1H), 2.42 (s, 6H). ^13^C{^1^H} NMR (100 MHz, CDCl_3_): δ = 146.6, 144.1, 141.8, 139.2, 138.4, 137.1, 136.3, 135.5, 131.1, 129.6, 128.8, 128.7, 128.5, 128.0, 127.1, 126.3, 114.9, 21.5. HRMS (ESI) *m/z*: [M + H]^+^ Calculated for C_26_H_23_ 335.1794; Found 335.1794.

##### 4.5.4.9 1,3-Bis(3,5-dimethylphenyl)-4,6-di-p-tolyl-1,2-dihydropentalene (**12aa**) and 4,6-Bis(3,5-dimethylphenyl)-1,3-di-p-tolyl-1,2-dihydropentalene (**12ab**)

Cherry-red solid (204 mg, 4.14 × 10^–4^ mol, 51% yield based on 8.15 × 10^–4^ mol **4**), reaction time 65.5 h. *R*_f_ = 0.22 (20:1 cyclohexane/toluene). ^1^H NMR (400 MHz, CDCl_3_): δ = 7.36–7.33 (m, 2H), 7.33–7.30 (m, 3H), 7.23–7.20 (m, 3H), 7.19–7.16 (m, 3H), 7.10–7.08 (m, 1H), 7.05–7.02 (m, 3H), 7.00 (s, 1H), 7.00–6.97 (m, 1H), 6.93–6.90 (m, 5H), 6.89 (s, 1H), 6.85 (s, 1H), 6.82 (s, 1H), 6.80–6.78 (m, 1H), 6.76 (s, 1H), 4.52 (d,^3^*J*_HH_ = 6.7 Hz, 1H), 4.48 (d,^3^*J*_HH_ = 6.7 Hz, 1H), 4.12–4.04 (overlapping dd’s as m, 2H), 3.44 (d,^2^*J*_HH_ = 18.8 Hz, 2H), 2.35, 2.34, 2.32, 2.30, 2.27, 2.27, 2.21, 2.14, 2.07 (all s, 36H in total). ^13^C{^1^H} NMR (100 MHz, CDCl_3_): δ = 154.6, 154.1, 148.8, 147.5, 146.8, 146.5, 144.8, 142.0, 139.6, 138.8, 138.7, 138.1, 137.5, 137.3, 137.2, 136.3, 135.8, 135.7, 135.6, 134.8, 134.5, 132.9, 132.8, 131.1, 130.5, 130.3, 130.1, 129.8, 129.7, 129.4, 129.2, 129.0, 128.6, 128.5, 128.4, 128.3, 128.1, 127.8, 127.6, 127.5, 126.5, 126.4, 125.3, 124.6, 55.4, 54.9, 42.7, 42.6, 21.5, 21.4, 21.3, 21.2, 21.1. ^1^H DOSY NMR (500 MHz, CDCl_3_): δ = (5.99 ± 0.03) × 10^–10^ m^2^ s^–1^. HRMS (ESI) *m/z*: [M + H]^+^ Calculated for C_38_H_37_ 493.2890; Found 493.2873.

##### 4.5.4.10 1,3-Di-p-tolyl-6-(3,5-dimethylphenyl)pentafulvene (**12b**)

Red solid (73 mg, 2.01 × 10^–4^ mol, 25% yield based on 8.15 × 10^–4^ mol **4**). Melting point 113–114 °C. reaction time 65.5 h, *R*_f_ = 0.34 (20:1 cyclohexane/toluene). ^1^H NMR (400 MHz, CDCl_3_): δ = 7.64–7.61 (m, 2H), 7.40–7.37 (m, 2H), 7.29–7.21 (overlapping s’s and m’s, 7H), 7.04 (s, 2H), 6.97–6.95 (m, 1H), 2.44 (s, 3H), 2.41–2.39 (overlapping singlets, 9H). ^13^C{^1^H} NMR (100 MHz, CDCl_3_): δ = 146.6, 144.3, 141.8, 138.5, 138.3, 137.9, 137.3, 136.8, 133.4, 132.8, 130.9, 129.5, 129.4, 129.2, 128.7, 127.6, 126.2, 113.8, 21.5, 21.3. HRMS (ESI) *m/z*: [M + H]^+^ Calculated for C_28_H_27_ 363.2107; Found 363.2106.

##### 4.5.4.11 1,3-Diphenyl-6-o-tolylpentafulvene (**14b**)

Dark red solid (108 mg, 0.34 mmol, 68% based on 0.5 mmol **4**). Melting point 89–90 °C. Reaction time 64 h, *R*_f_ = 0.62 (6:1 cyclohexane/toluene). ^1^H NMR (500 MHz, CDCl_3_): δ = 7.71–7.67 (m, 2H), 7.64–7.61 (m, 1H), 7.53–7.23 (several m, 12H), 7.05–7.03 (m, 1H), 6.89 (s, 1H), 2.35 (s, 3H). ^13^C{^1^H} NMR (125 MHz, CDCl_3_): δ = 146.4, 144.7, 141.1, 138.1, 137.6, 136.3, 136.2, 135.4, 131.5, 130.3, 129.4, 129.1, 128.8, 128.6, 128.5, 128.0, 127.1, 126.2, 126.1, 115.4, 20.3. HRMS (ESI) *m/z*: [M + H]^+^ Calculated for C_25_H_21_ 321.1638; Found 321.1639.

##### 4.5.4.12 1,3-Bis(4-methoxyphenyl)-4,6-diphenyl-1,2-dihydropentalene (**15a**)

Cherry-red solid (266 mg, 0.56 mmol, 51% based on 1.12 mmol **4**). Melting point 149–150 °C. Reaction time 61 h, *R*_f_ = 0.11 (2:1 cyclohexane/toluene). ^1^H NMR (500 MHz, CDCl_3_): δ = 7.41 (d,^3^*J*_HH_ = 7.8 Hz, 2H), 7.33 (s, 1H), 7.30–7.13 (m, 12H), 7.08 (m, 1H), 6.79 (m, 2H), 6.79 (m, 2H), 6.64 (m, 2H), 4.55 (d,^3^*J*_HH_ = 6.7 Hz, 1H), 4.09 (dd,^3^*J*_HH_ = 6.7 Hz,^2^*J*_HH_ = 18.5 Hz, 1H), 3.76, 3.74 (all s, 6H), 3.39 (d,^2^*J*_HH_ = 18.5 Hz, 1H). ^13^C{^1^H} NMR (125 MHz, CDCl_3_): δ = 160.9, 158.2, 154.5, 149.0, 138.9, 137.2, 136.6, 135.4, 132.2, 130.2, 130.0, 129.8 (via HMBC), 129.5, 129.4, 128.8, 128.4, 128.4, 128.4, 128.2, 128.0, 127.8, 127.7, 126.4, 126.2, 126.0, 114.1, 113.4, 55.4, 55.3, 55.1, 41.9. HRMS (ESI) *m/z*: [M + Na]^+^ Calculated for C_34_H_28_O_2_Na 491.1982; Found 491.1977.

##### 4.5.4.13 1,3-Diphenyl-6-(4-methoxyphenyl)pentafulvene (**15b**)

Orange-red solid (116 mg, 3.45 × 10^–4^ mol, 31% based on 1.12 mmol **4**). Reaction time 61 h, *R*_f_ = 0.42 (2:1 cyclohexane/toluene). ^1^H NMR (400 MHz, CDCl_3_): δ = 7.74–7.70 (m, 2H), 7.65–7.61 (m, 2H), 7.49–7.28 (m, 8H), 7.22 (s, 1H), 7.10–7.06 (m, 1H), 7.00–6.96 (m, 3H), 3.88 (s, 3H)—as previously reported.^25^

##### 4.5.4.14 1,3-Bis(4-fluorophenyl)-4,6-di-p-tolyl-1,2-dihydropentalene (**16aa**) and 4,6-Bis(4-fluoro-phenyl)-1,3-di-p-tolyl-1,2-dihydropentalene (**16ab**)

Cherry-red solid (260 mg, 5.50 × 10^–4^ mol, 55% based on 1.01 mmol **5**). Reaction time 45.5 h, *R*_f_ = 0.22 (20:1 cyclohexane/toluene).^1^H NMR (500 MHz, CDCl_3_): δ = 7.36–7.32 (m, 2H), 7.29–7.13 (overlapping m’s, 15H), 7.12–7.09 (m, 2H), 7.07–7.04 (m, 2H), 7.02–6.91 (overlapping m’s, 8H), 6.89–6.84 (m, 3H), 6.84–6.79 (m, 2H), 4.56 (d,^3^*J*_HH_ = 6.9 Hz, 1H), 4.51 (d,^3^*J*_HH_ = 6.7 Hz, 1H), 4.11–4.05 (overlapping dd’s,^3^*J*_HH_ = 6.9 Hz,^3^*J*_HH_ = 6.7 Hz,^2^*J*_HH_ = 18.6 Hz,^2^*J*_HH_ = 18.9 Hz, 2H), 3.40 (d,^2^*J*_HH_ = 18.9 Hz, 1H), 3.33 (d,^2^*J*_HH_ = 18.6 Hz, 1H), 2.32, 2.30, 2.29, 2.27 (all s, 12H). ^1^H DOSY NMR (500 MHz, CDCl_3_): D = (6.77 ± 0.04) × 10^–10^ m^2^ s^–1^. ^13^C{^1^H} NMR (125 MHz, CDCl_3_): δ = 163.5 (d,^1^*J*_CF_ = 250.4 Hz), 161.8 (d,^1^*J*_CF_ = 244.8 Hz), 161.5 (d,^1^*J*_CF_ = 244.4 Hz), 161.4 (d,^1^*J*_CF_ = 245.6 Hz), 155.2, 151.9, 148.2, 146.7, 145.7, 141.1, 140.2, 140.0, 139.2, 138.8, 136.15, 136.08, 133.7, 133.0, 132.2, 132.15, 132.08, 131.5, 131.4, 130.5, 130.3, 130.2, 129.9, 129.8, 129.6, 129.4, 129.2, 128.9, 128.83, 128.80, 128.7, 128.2, 128.0, 127.9, 127.3, 126.4, 115.6 (d,^2^*J*_CF_ = 21.2 Hz), 115.3 (d,^2^*J*_CF_ = 21.4 Hz), 115.1 (d,^2^*J*_CF_ = 21.8 Hz), 114.9 (d,^2^*J*_CF_ = 21.1 Hz), 55.3, 55.2, 42.3, 41.9, 21.5, 21.3, 21.2. ^19^F{^1^H} NMR (470 MHz, CDCl_3_): δ = −110.7, −116.2, −116.8, −116.8. HRMS (ESI) *m/z*: [M + H]^+^ Calculated for C_34_H_27_F_2_ 473.2075; Found 473.2058.

##### 4.5.4.15 1,3-Ditolyl-6-(4-fluorophenyl)pentafulvene (**16b**)

Orange-red solid (32 mg, 9.08 × 10^–5^ mol, 9% based on 1.01 mmol **5**). Reaction time 45.5 h, *R*_f_ = 0.33 (20:1 cyclohexane/toluene). ^1^H NMR (500 MHz, CDCl_3_): δ = 7.63–7.58 (m, 4H), 7.37–7.34 (m, 2H), 7.27–7.24 (m, 2H), 7.27–7.24 (m, 2H), 7.22–7.19 (m, 2H), 7.17 (s, 1H), 7.15–7.10 (m, 2H), 6.95 (s, 1H), 6.93 (s, 1H), 2.42 (s, 3H), 2.38 (s, 3H). ^13^C{^1^H} NMR (125 MHz, CDCl_3_): δ = 163.3 (d,^1^*J*_CF_ = 250.6 Hz), 147.2, 144.4, 141.7, 138.1, 137.0, 136.5, 133.2, 132.6, 132.5, 129.5, 129.4, 129.3, 127.8, 126.2, 116.1, 115.9, 113.2, 21.5, 21.4. ^19^F{^1^H} NMR (470 MHz, CDCl_3_): δ = −111.5. HRMS (ESI) *m/z*: [M + H]^+^ Calculated for C_26_H_22_F 353.1700; Found 353.1700.

##### 4.5.4.16 1,3-Di-*p*-tolyl-4,6-bis(4-(trifluoromethyl)phenyl)-1,2-dihydropentalene (**17**)

Dark red powder (23 mg, 4.02 × 10^–5^ mol, 5% based on 8.08 × 10^–5^ mol **5**). Melting point 165–166 °C. Reaction time 40.5 h. ^1^H NMR (400 MHz, CDCl_3_): δ = 7.49–7.38 (m, 7H), 7.35–7.31 (m, 2H), 7.18–7.13 (m, 4H), 7.09–7.05 (m, 2H), 6.98–6.94 (m, 2H), 4.57 (d,^3^*J*_HH_ = 6.6 Hz, 1H), 4.13 (dd,^3^*J*_HH_ = 6.6 Hz,^2^*J*_HH_ = 19.3 Hz, 1H), 3.46 (d,^2^*J*_HH_ = 19.3 Hz, 1H), 2.32, 2.30 (s, 3H). ^13^C{^1^H} NMR (125 MHz, CDCl_3_): δ = 157.9, 152.2, 145.3, 141.0, 140.6, 140.2, 139.8, 138.5, 136.4, 131.9, 130.3, 129.7, 129.3, 129.1, 128.9, 128.6, 128.5, 127.6 (*C*F_3_ via HMBC), 127.2, 126.4, 125.7, 125.4 (m, *C*H-C-CF_3_), 125.3 (*C*F_3_ via HMBC), 125.1 (m, *C*H-C-CF_3_), 55.4, 42.7, 21.6, 21.2. ^19^F{^1^H} NMR (470 MHz, CDCl_3_): δ = −62.2, −62.3. HRMS (ESI) *m/z*: [M – H]^−^ Calculated for C_36_H_25_F_6_ 571.1865; Found 571.1856.

##### 4.5.4.17 3-Methyl-1,4,6-triphenyldihydropentalene (**19**)

Red solid (1.25 g, 3.62 mmol, 78% based on 4.62 mmol **4**). Melting point 151–152 °C. Reaction time 42 h, 1 equiv pyrrolidine. ^1^H NMR (500 MHz, C_6_D_6_): δ = 7.45–7.42 (m, 2H), 7.39–7.36 (m, 2H), 7.28 (s, 1H), 7.19–7.15 (m, 1H), 7.07–7.03 (m, 4H), 7.00–6.92 (m, 4H), 6.87–6.81 (m, 2H), 4.13 (d, 1H,^3^*J*_HH_ = 6.7 Hz), 3.02 (dd, 1H,^2^*J*_HH_ = 19.6 Hz, ^3^*J*_HH_ = 6.7 Hz), 2.53 (d, 1H,^2^*J*_HH_ = 19.6 Hz), 1.64 (s, 3H). ^13^C{^1^H} NMR (125 MHz, C_6_D_6_): δ = 156.0, 148.3, 147.9, 144.7, 137.52, 137.48, 136.0, 130.8, 129.7, 129.0, 128.8, 128.6, 128.5, 128.3, 128.0, 127.6, 126.8, 126.7, 126.6, 126.3, 57.6, 43.4, 16.8. HRMS (ESI) *m/z*: [M + H]^+^ Calculated for C_27_H_23_ 347.1800; Found 347.1799.

##### 4.5.4.18 1,3-Dimethyl-4,6-diphenyldihydropentalene (**20**)

Red solid (335 mg, 1.18 mmol, 75% based on 1.58 mmol **4**). Melting point 122–123 °C. Reaction time 20 h, 1 equiv pyrrolidine. ^1^H NMR (500 MHz, CDCl_3_): δ = 7.58 (d, 2H,^3^*J*_HH_ = 7.5 Hz), 7.46 (d, 2H,^3^*J*_HH_ = 7.4 Hz), 7.36 (m, 4H), 7.25 (t, 1H,^3^*J*_HH_ = 7.3 Hz), 7.19 (t, 1H,^3^*J*_HH_ = 7.3 Hz), 7.15 (s, 1H), 3.53–3.40 (m, 2H), 2.69 (d, 1H,^2^*J*_HH_ = 19.2 Hz), 2.20 (s, 3H), 1.27 (d, 3H,^3^*J*_HH_ = 7.0 Hz). ^13^C{^1^H} NMR (125 MHz, CDCl_3_): δ = 157.6, 151.6, 146.5, 137.2, 137.0, 136.2, 129.6, 128.6, 128.31, 128.30, 127.8, 126.34, 126.28, 125.9, 55.5, 31.6, 19.5, 17.5. HRMS (ESI) *m/z*: [M + H]^+^ Calculated for C_22_H_21_ 285.1643; Found 285.1640.

##### 4.5.4.19 1-(3,5-Dimethylphenyl)-6-phenyl-4-(p-tolyl)-3-(4-(trifluoromethyl)phenyl)-1,2-dihydropentalene-Based Isomer Mixture (**23a**)

Cherry-red solid (133 mg, 2.56 × 10^–4^ mol, 48% based on 5.35 × 10^–4^ mol **7**). Reaction time 68 h, *R*_f_ = 0.39 (10:1 cyclohexane/toluene). ^1^H NMR (500 MHz, CDCl_3_): δ = 7.53–6.74 (overlapping multiplets and singlets, 17H), 4.69–4.48 (overlapping doublets, 1H), 4.20–4.04 (overlapping dd’s, 1H,), 3.51–3.36 (overlapping doublets, 1H), 2.38–2.04 (overlapping singlets, 9H). ^13^C{^1^H} NMR (125 MHz, CDCl_3_): δ = 156.9, 156.6, 155.6, 155.5, 154.3, 154.0, 150.9, 150.7, 149.9, 149.8, 148.8, 148.7, 148.4, 147.9, 146.74, 146.68, 146.5, 146.2, 146.1, 145.5, 144.7, 144.1, 144.0, 141.5, 141.2, 141.0, 140.8, 140.5, 140.4, 140.2, 139.6, 139.2, 138.9, 138.8, 138.43, 138.38, 138.3, 137.8, 137.7, 137.6, 137.5, 137.4, 137.2, 137.1, 136.3, 136.2, 136.03, 135.99, 135.9, 135.7, 135.3, 135.2, 135.1, 134.8, 134.7, 134.43, 134.37, 134.1, 132.4, 132.3, 132.2, 131.5, 131.03, 130.97, 130.4, 130.3, 130.2, 130.2, 130.0, 129.9, 129.7, 129.5, 129.4, 129.3, 129.2, 129.0, 128.9, 128.7, 128.65, 128.60, 128.57, 128.5, 128.42, 128.39, 128.30, 128.27, 128.2, 128.1, 128.01, 127.97, 127.9, 127.8, 127.6, 127.4, 127.2, 127.0, 126.7, 126.6, 126.5, 126.4, 126.3, 126.2, 125.9, 125.8, 125.4, 125.3, 125.2, 124.9, 124.6, 124.5, 55.6, 55.4, 55.3, 55.1, 54.5, 43.1, 43.0, 42.8, 42.7, 42.6, 42.4, 21.5, 21.5, 21.4, 21.3, 21.23, 21.18, 21.11, 21.06. ^19^F{^1^H} NMR (470 MHz, CDCl_3_): δ = −62.2, −62.2, −62.3, −62.3, −62.3, −62.8, −62.8. ^1^H DOSY NMR (500 MHz, CDCl_3_): *D* = (7.03 ± 0.04) × 10^–10^ m^2^ s^–1^. HRMS (ESI) *m/z*: [M + H]^+^ Calculated for C_36_H_30_F_3_ 519.2294; Found 519.2289.

##### 4.5.4.20 1-(Phenyl)-3-(p-tolyl)-6-(3,5-dimethylphenyl)pentafulvene and 1-(p-Tolyl)-3-(phenyl)-6-(3,5-dimethyl-phenyl)pentafulvene (**23b**)

Orange-red solid (35 mg, 1.00 × 10^–4^ mol, 19% based on 5.35 × 10^–4^ mol **7**). Reaction time 68 h, *R*_f_ = 0.49 (10:1 cyclohexane/toluene). ^1^H NMR (500 MHz, CDCl_3_): δ = 7.74–7.70 (m, 1H), 7.64–7.60 (m, 1H), 7.50–7.36 (m, 5H), 7.33–7.20 (m, 5H), 7.06–6.96 (m, 3H), 2.45–2.37 (s and two overlapping s, 9H), 2.08 (s, <3H). ^13^C{^1^H} NMR (125 MHz, CDCl_3_): δ = 146.65, 146.56, 144.20, 144.16, 141.8, 139.0, 138.7, 138.4, 137.9, 137.21, 137.18, 136.9, 136.3, 135.6, 133.3, 132.7, 131.04, 131.00, 129.8, 129.6, 129.5, 129.4, 129.3, 128.8, 128.7, 128.5, 128.1, 128.0, 127.8, 127.5, 127.05, 126.96, 126.3, 126.2, 114.7, 114.0, 21.5, 21.4, 21.2. HRMS (ESI) *m/z*: [M + H]^+^ Calculated for C_27_H_25_ 349.1951; Found 349.1952.

## Notes

The data underlying this study are available in the published article and its online supplementary material.

## Supporting Information Available

Alternative synthesis protocols for 1,3,4,6-tetra-*p*-tolyl-1,2-dihydropentalene and synthesis protocol for 1,3-di-*p*-tolyl-4,6-bis(4-(trifluoromethyl)phenyl)-1,2-dihydropentalene and additional analytical data (NMR spectra and UV–vis comparison) (PDF)
