# Supplementary material for: Connect Four: Tetraarylated Dihydropentalenes and Triarylated Monocyclic Pentafulvenes from Cyclopentadienes and Enones
Source: J Org Chem. 2022 Oct 5;87(21):13790–802. doi: 10.1021/acs.joc.2c01507 (PMC9639016; doi:10.1021/acs.joc.2c01507)
Supplement: Supplementary file 1 — jo2c01507_si_001.pdf [file jo2c01507_si_001.pdf]

Connect Four: Tetraarylated Dihydropentalenes and Triarylated Monocyclic Pentafulvenes from Cyclopentadienes and Enones

Niko A. Jenek,[a] Marek Balschun,[a] Stuart M. Boyt[a] and Ulrich Hintermair*[a,b]

a) *Department of Chemistry, University of Bath, Claverton Down, Bath BA2 7AY, UK.*

b) *Centre for Sustainable Chemical Technologies, University of Bath, Claverton Down, Bath, BA2 7AY, UK.*

* u.hintermair@bath.ac.uk

SUPPORTING INFORMATION

Table of contents

1. Alternative synthesis route for 1,2-dihydropentalenes S2
2. NMR spectra of starting materials S4
3. NMR spectra of products S10
4. UV-Vis spectroscopic comparison between selected dihydropentalenes and pentafulvenes ... S34

1. Alternative synthesis route for 1,2-dihydropentalenes

1,3,4,6-tetra-p-tolyl-1,2-dihydropentalene

<u>Route via fulvene</u>*:* To a solution of 1,3,6-tritoluylfulvene (50 mg, 0.14 mmol) and 1-(p-tolyl)ethan-1-one (44 mg, 0.33 mmol) in 1.75 mL methanol and 1.75 mL toluene under argon was added pyrrolidine dropwise over range of 4 minutes (53 mg, 0.75 mmol). The resulting mixture was stirred at 75 °C for 44 hours. After cooling down to room temperature, the solvent was removed under reduced pressure. The crude material was dissolved in a minimum of 3:1 diethyl ether/*n*-hexane, followed by drying-filtering through neutral silica using 3:1 diethyl ether/*n*-hexane as the eluent, collecting the first dark red-violet band only. This fraction was further purified, *via* preparative thin layer chromatography (5:1 cyclohexane/toluene systems as eluent). The orange first band consisted of traces (<1 mg) of the starting material, while the purple second band gave the corresponding 1,2-dihydropentalene (3 mg, $6.85 \cdot 10^{-6}$ mol, 5%).

<u>Route via NaO^{t}Bu activation</u>*:* To a pale-yellow stirred solution of 1,4-*p-tolyl*-1,3-cyclopentadiene (108 mg, 0.44 mmol) in 7.5 mL dry THF inside a glovebox, a solution of sodium *tert*-butoxide (43 mg, 0.45 mmol) in 2.5 mL dry THF was added dropwise over a range of six minutes. After 65 minutes of stirring, to this now golden-yellow solution was added a solution of 1,3-di-*p*-tolylprop-2-en-1-one (194 mg, 0.82 mmol, 1.87 eq.) in 8 mL dry THF was added dropwise over a range of 25 minutes. The resulting solution was stirred for 1.5 hours at room temperature, after which it was transferred into a Cajon Schlenk flask, the flask sealed, taken out of the glove box, and stirred for further 18 hours at room temperature, followed by stirring at 81 °C for 23 hours. After cooling down to room temperature, the mixture was quenched with 0.1 mL $NH_4Cl_{aq.sat.}$ and stirred fur further 30 minutes, followed by a dilution with 30 mL diethyl ether and 30 mL water. The organic phase was washed with 2 x 15 mL water and 15 mL brine. The solvent was removed under reduced pressure and the fraction was then redissolved in a minimum of 2:1 hexane/diethyl ether and filtered through silica using 2:1 hexane/diethyl ether as the eluent, collecting the first broad dark red-violet band only. This fraction

was further purified *via* preparative thin layer chromatography (5:1 cyclohexane/toluene as eluent). The orange first band consisted of 1,3,6-tritoluylfulvene (9 mg, $2.60 \cdot 10^{-5}$ mol, 7%), while the purple second band gave the corresponding 1,2-dihydropentalene (44 mg). An additional third black band consisted of a mixture of an unknown compound and the desired dihydropentalene. This band was further purified *via* a micro-column (silica, 20:1 → 5:1 cyclohexane/toluene gradients), resulting in a second crop of the desired 1,2-dihydropentalene (23 mg; 67 mg in total, 0.15 mmol, 33%).

4,6-di-p-tolyl-1,3-bis(4-(trifluoromethyl)phenyl)-1,2-dihydropentalene

To a pale-yellow stirred solution of 1,4-*p-tolyl*-1,3-cyclopentadiene (199 mg, $8.08 \cdot 10^{-4}$ mol) in 14 mL dry THF inside a glovebox, a solution of sodium *tert*-butoxide (79 mg, $8.11 \cdot 10^{-4}$ mol) in 5 mL dry THF was added dropwise over a range of four minutes. After 45 minutes of stirring, the now golden-yellow solution was transferred onto a Schlenk line and stirred for further 15 minutes. To this solution was added a solution of 1,3-bis(4-(trifluoromethyl)phenyl)prop-2-en-1-one (552 mg, 1.60 mmol, 1.99 eq.) in 16 mL dry THF was added dropwise over a range of 44 minutes. The resulting solution was stirred for 19.5 hours at room temperature, then 21 hours at 75 °C. After cooling down to room temperature, the mixture was quenched with 0.17 mL $NH_4Cl_{aq.sat.}$ and stirred fur further 30 minutes, followed by a dilution with 60 mL diethyl ether and 60 mL water. The organic phase was washed with 2 x 30 mL water and 30 mL brine. The solvent was removed under reduced pressure and the fraction was then redissolved in a minimum of 3:1 hexane/diethyl ether and filtered through silica using 3:1 hexane/diethyl ether as the eluent, collecting the first broad dark red-violet band only. This fraction was further purified first *via* liquid flash chromatography (10:1 cyclohexane/toluene as eluent), collecting the first red-violet band only. The resulting dark-red solid was recrystallised in boiling methanol, let cool down to room temperature overnight and filtered, collecting the filtrate containing the corresponding 1,2-dihydropentalenes as cherry red powder after drying (23 mg, $4.02 \cdot 10^{-5}$ mol, 5%).

2. NMR spectra of starting materials

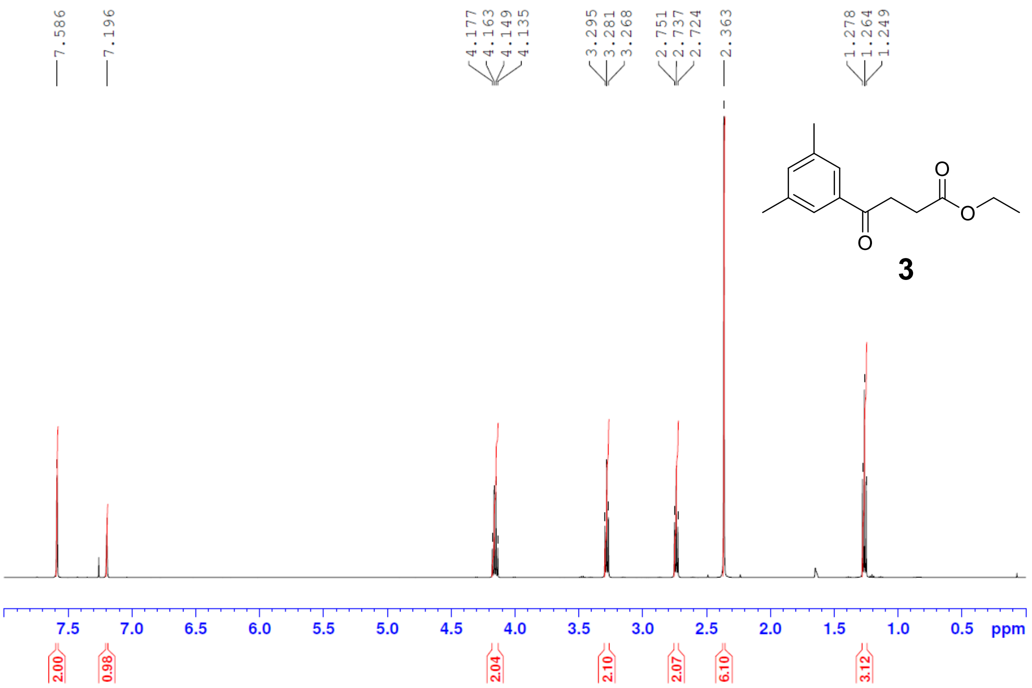

Figure S1: ^{1}H NMR of ethyl-4-(3,5-dimethylphenyl)-4-oxobutanoate **3** (500 MHz, $CDCl_3$, 298 K)

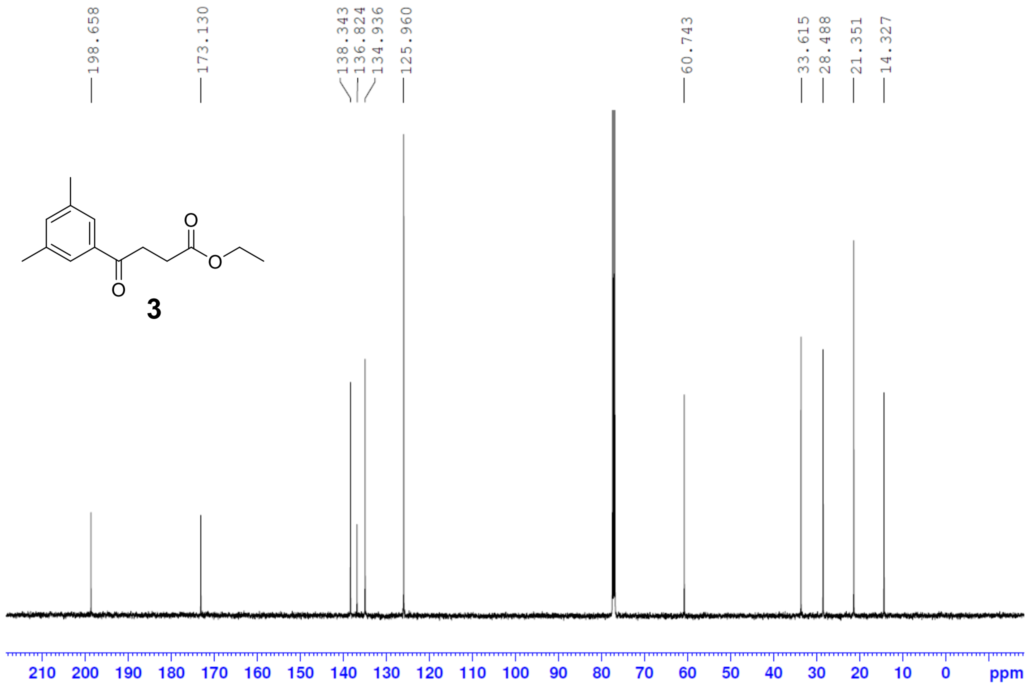

Figure S2: $^{13}C\{^{1}H\}$ NMR of ethyl-4-(3,5-dimethylphenyl)-4-oxobutanoate **3** (125 MHz, $CDCl_3$, 298 K)

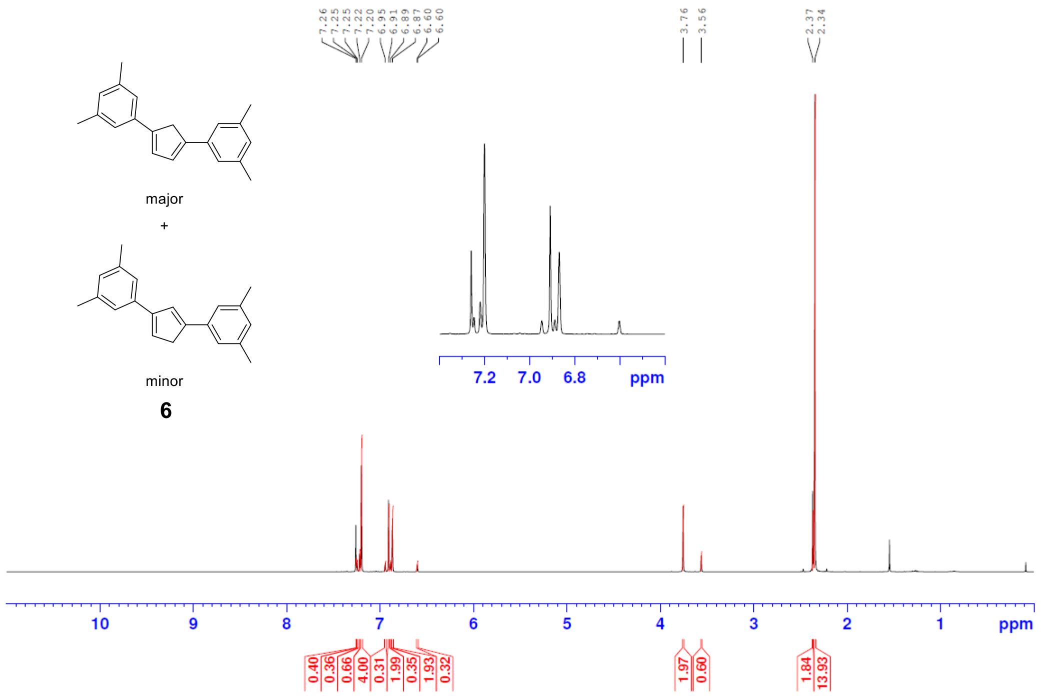

Figure S3: ^{1}H NMR of bis(3,5-dimethylphenyl)cyclopentadiene isomers **6** (500 MHz, $CDCl_3$, 298 K)

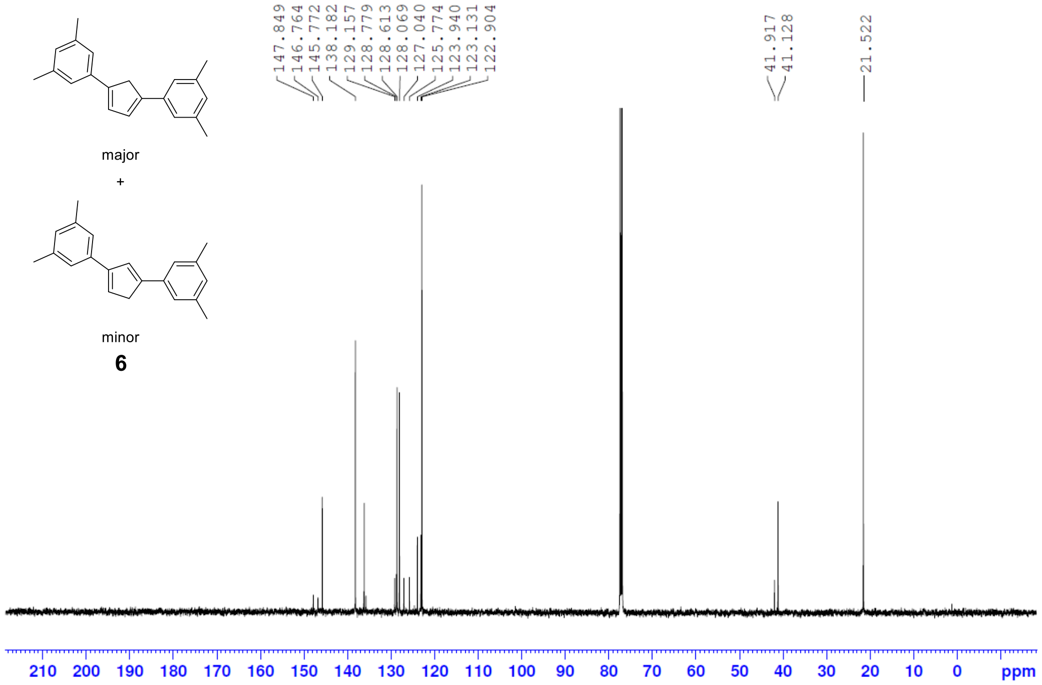

Figure S4: $^{13}C\{^{1}H\}$ NMR of bis(3,5-dimethylphenyl)cyclopentadiene isomers **6** (125 MHz, $CDCl_3$, 298 K)

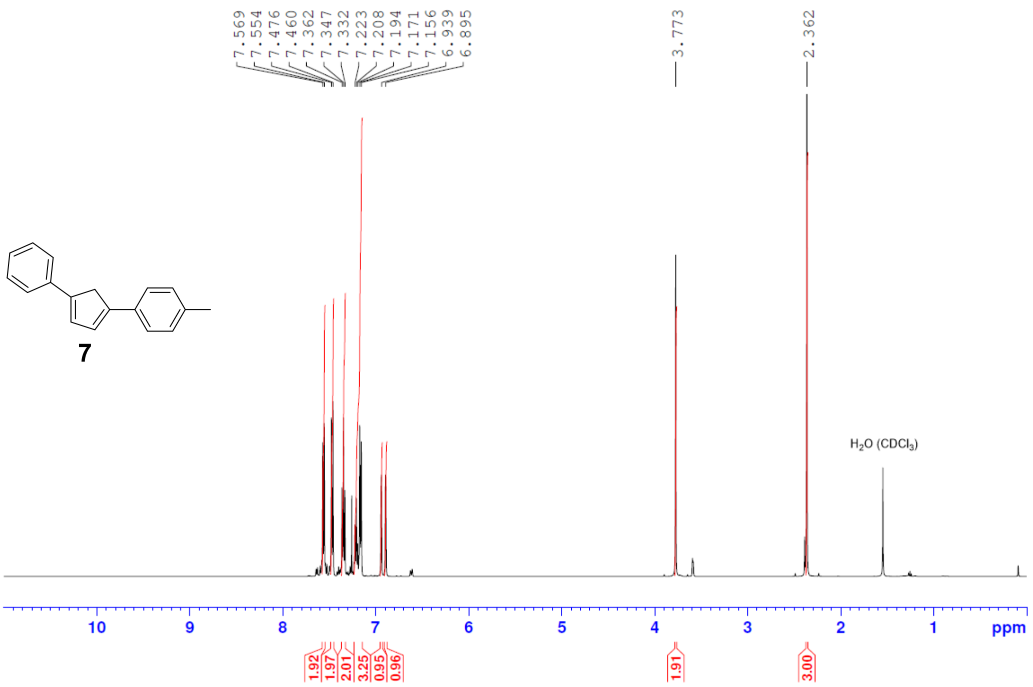

Figure S5: ^{1}H NMR of 1-Phenyl-4-*p*-tolyl-cyclopenta-1,3-diene **7** (500 MHz, $CDCl_3$, 298 K)

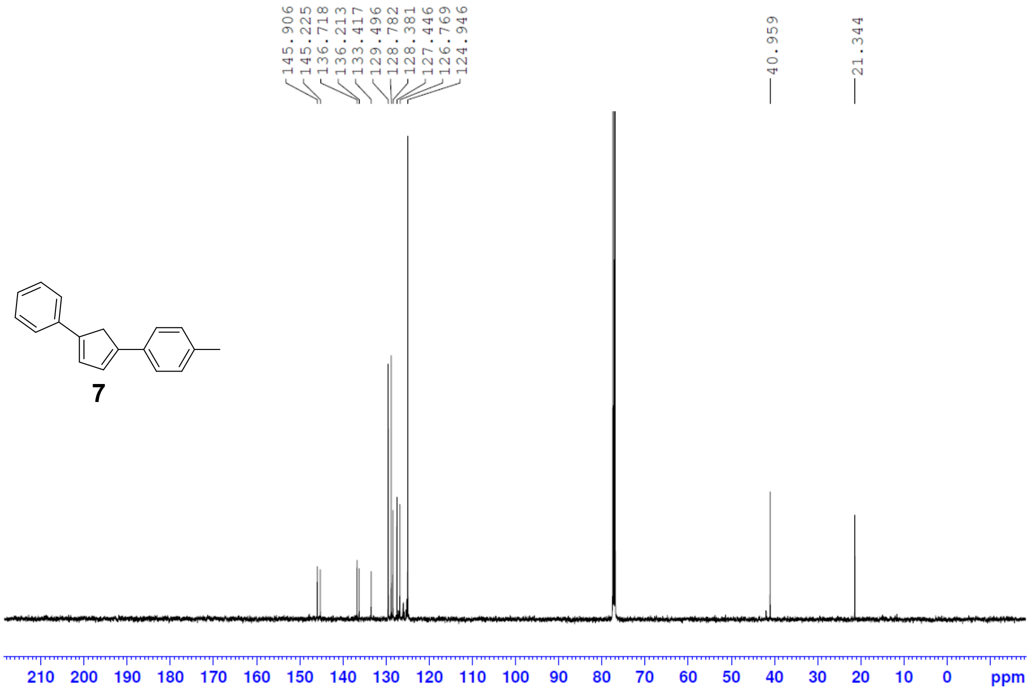

Figure S6: $^{13}C\{^{1}H\}$ NMR of 1-Phenyl-4-*p*-tolyl-cyclopenta-1,3-diene **7** (125 MHz, $CDCl_3$, 298 K)

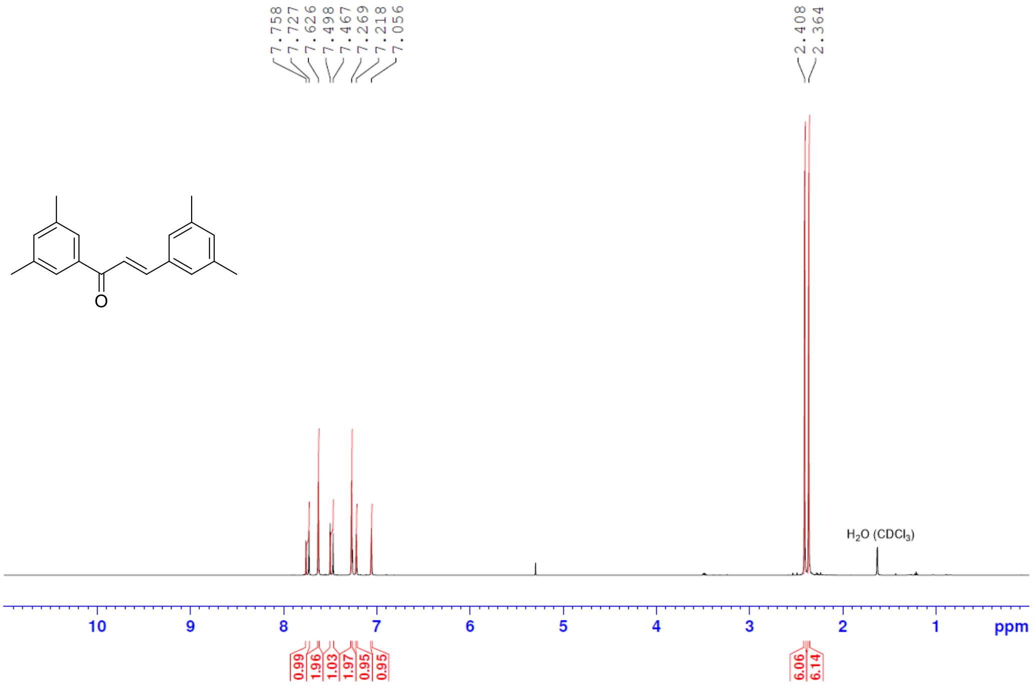

Figure S7: ^{1}H NMR of (*E*)-1,3-bis(3,5-dimethylphenyl)prop-2-en-1-one (500 MHz, $CDCl_3$, 298 K)

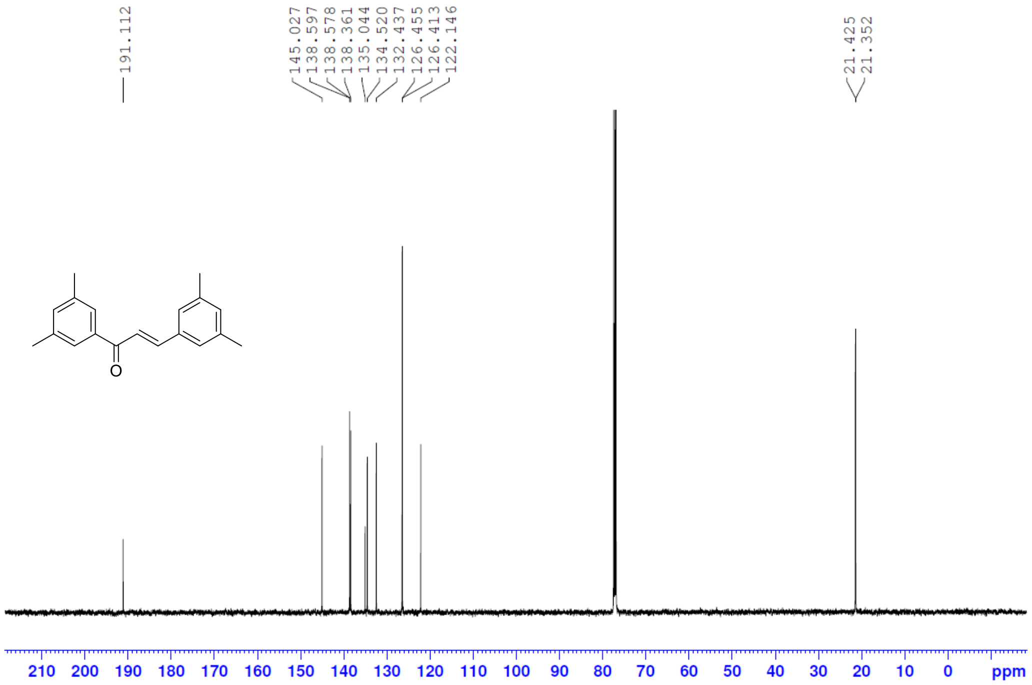

Figure S8: ^{13}C{^{1}H} NMR of (*E*)-1,3-bis(3,5-dimethylphenyl)prop-2-en-1-one (125 MHz, $CDCl_3$, 298 K)

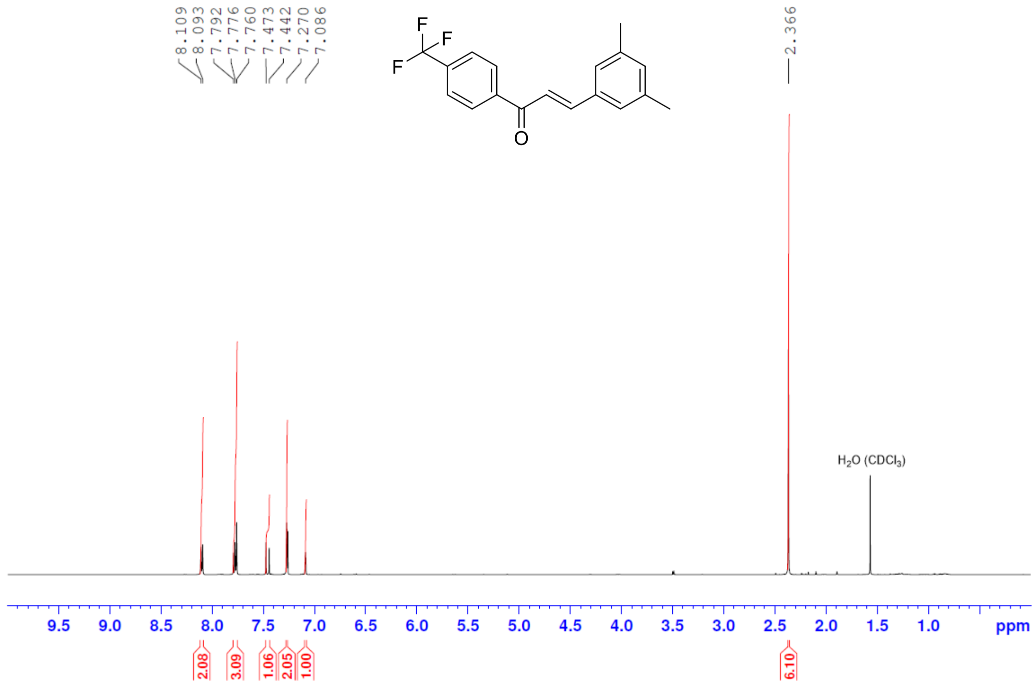

Figure S9: ^{1}H NMR of (*E*)-3-(3,5-dimethylphenyl)-1-(4-(trifluoromethyl)phenyl)prop-2-en-1-one

(500 MHz, $CDCl_3$, 298 K)

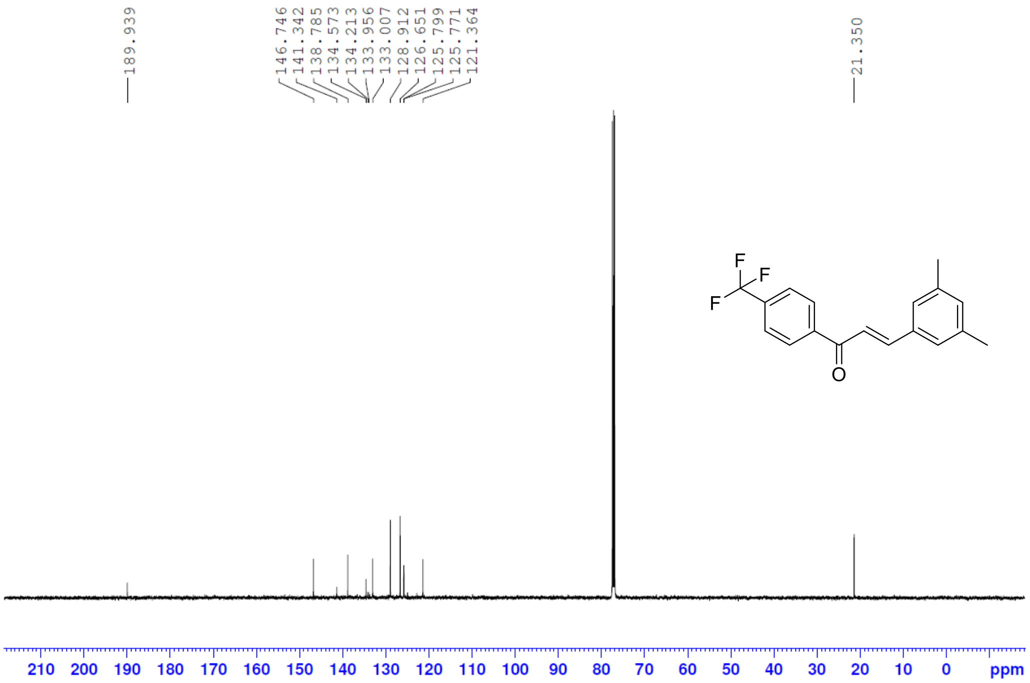

Figure S10: $^{13}C\{^{1}H\}$ NMR of (*E*)-3-(3,5-dimethylphenyl)-1-(4-(trifluoromethyl)phenyl)prop-2-en-1-one

(125 MHz, $CDCl_3$, 298 K)

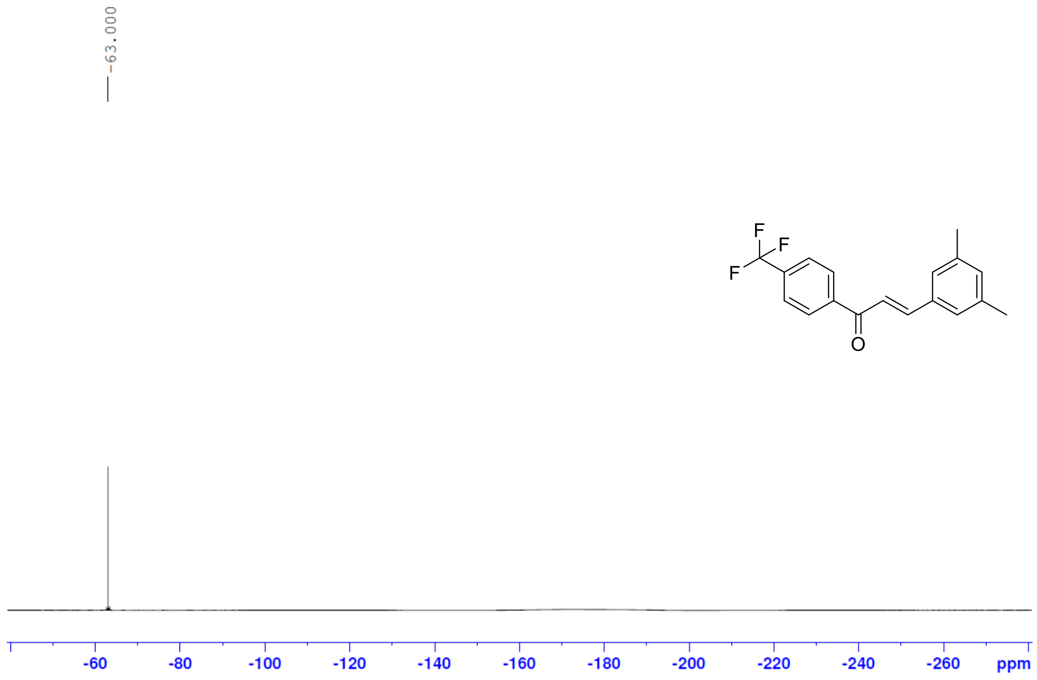

Figure S11: $^{19}F\{^{1}H\}$ NMR of (*E*)-3-(3,5-dimethylphenyl)-1-(4-(trifluoromethyl)phenyl)prop-2-en-1-one

(470 MHz, $CDCl_3$, 298 K)

3. NMR spectra of products

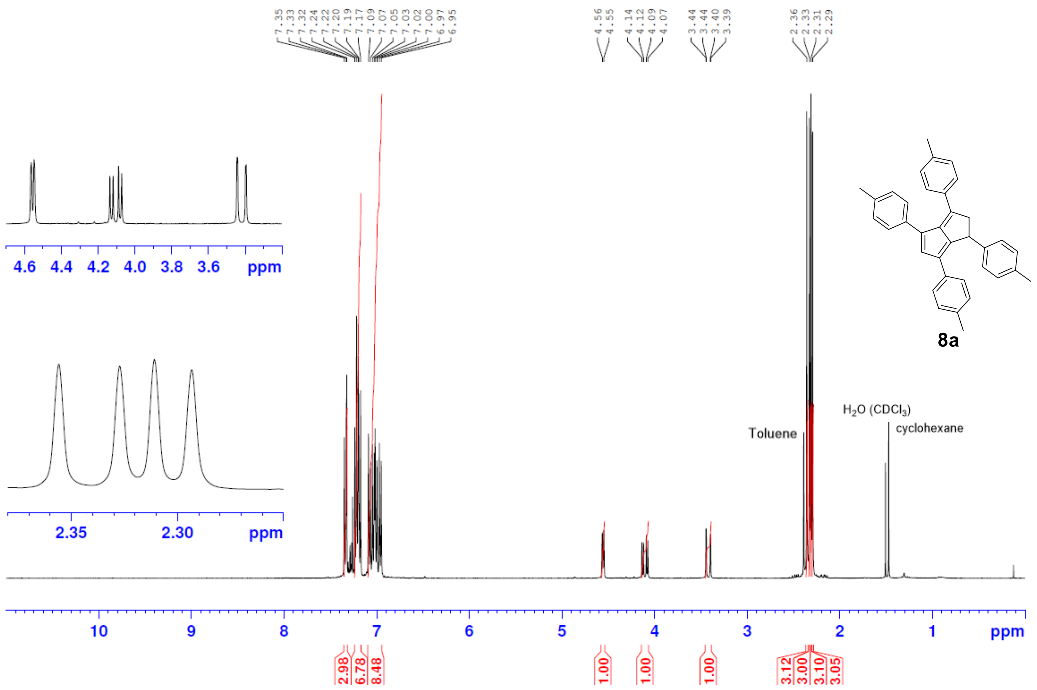

Figure S12: ^{1}H NMR of 1,3,4,6-tetra-*p*-tolyl-1,2-dihydropentalene **8a** (400 MHz, $CDCl_3$, 298 K)

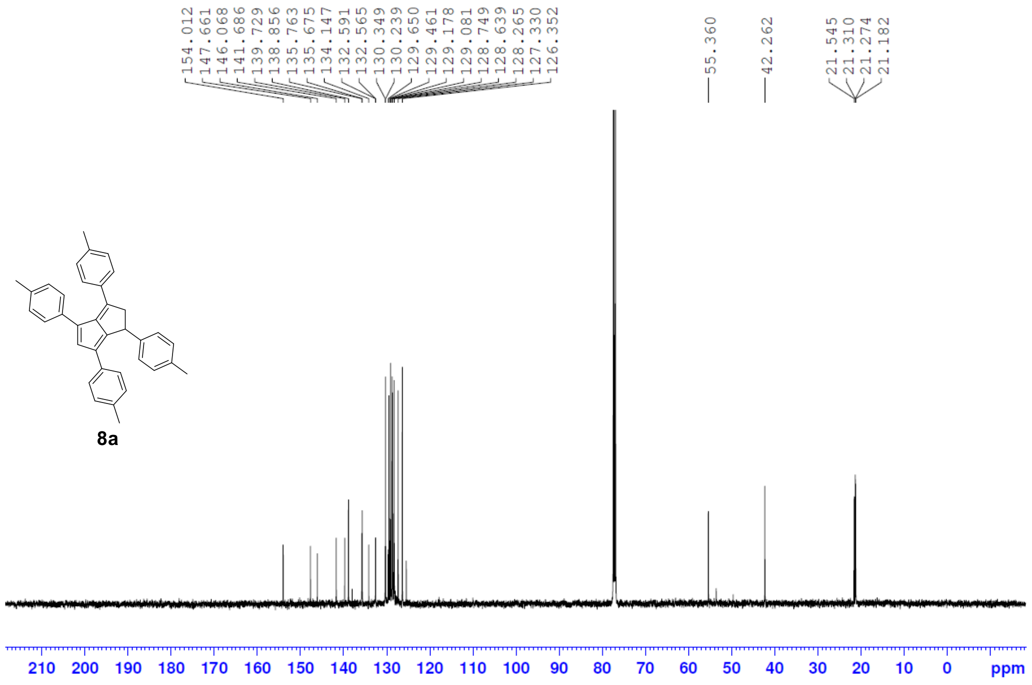

Figure S13: ^{13}C{^{1}H} NMR of 1,3,4,6-tetra-*p*-tolyl-1,2-dihydropentalene **8a** (100 MHz, $CDCl_3$, 298 K)

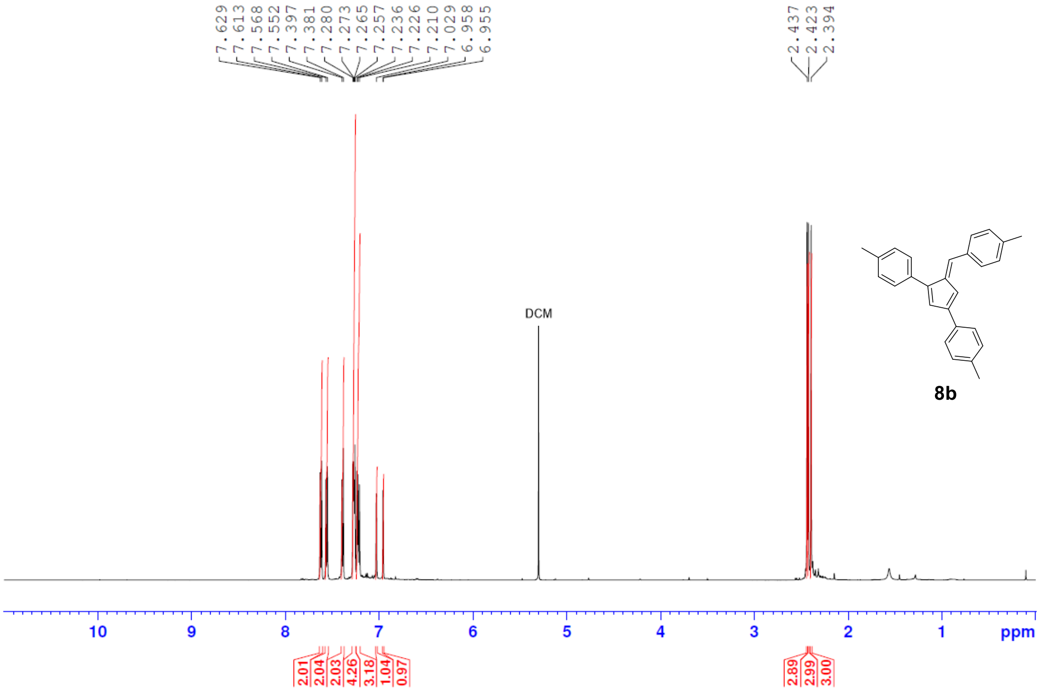

Figure S14: ^{1}H NMR of 1,3,6-tri-*p*-toluylfulvene **8b** (500 MHz, $CDCl_3$, 298 K)

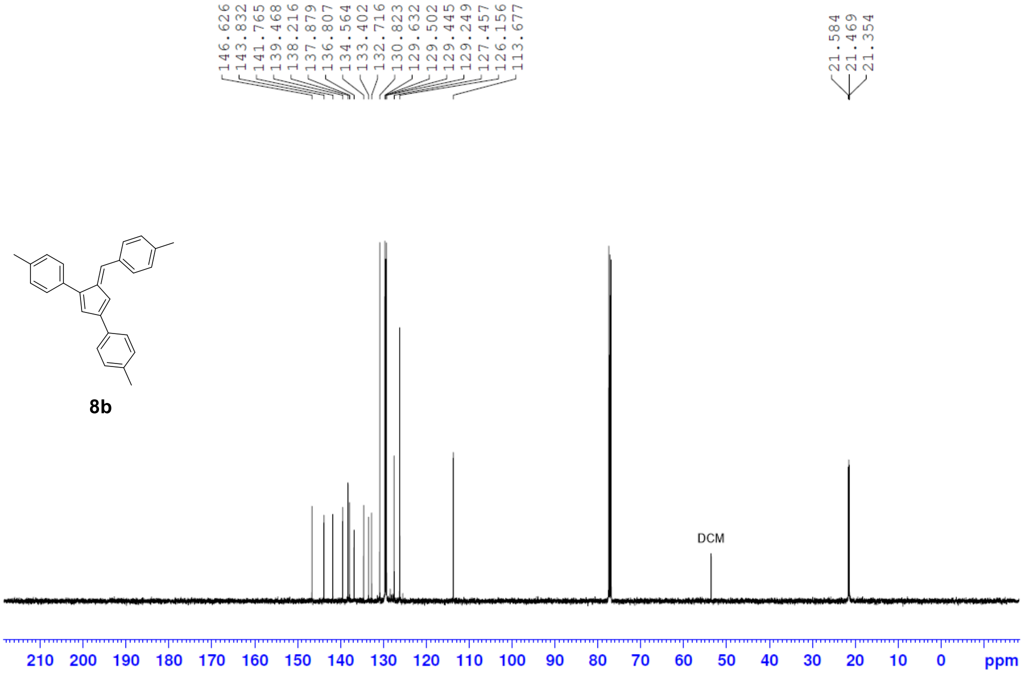

Figure S15: $^{13}C\{^{1}H\}$ NMR of 1,3,6-tri-*p*-toluylfulvene **8b** (125 MHz, $CDCl_3$, 298 K)

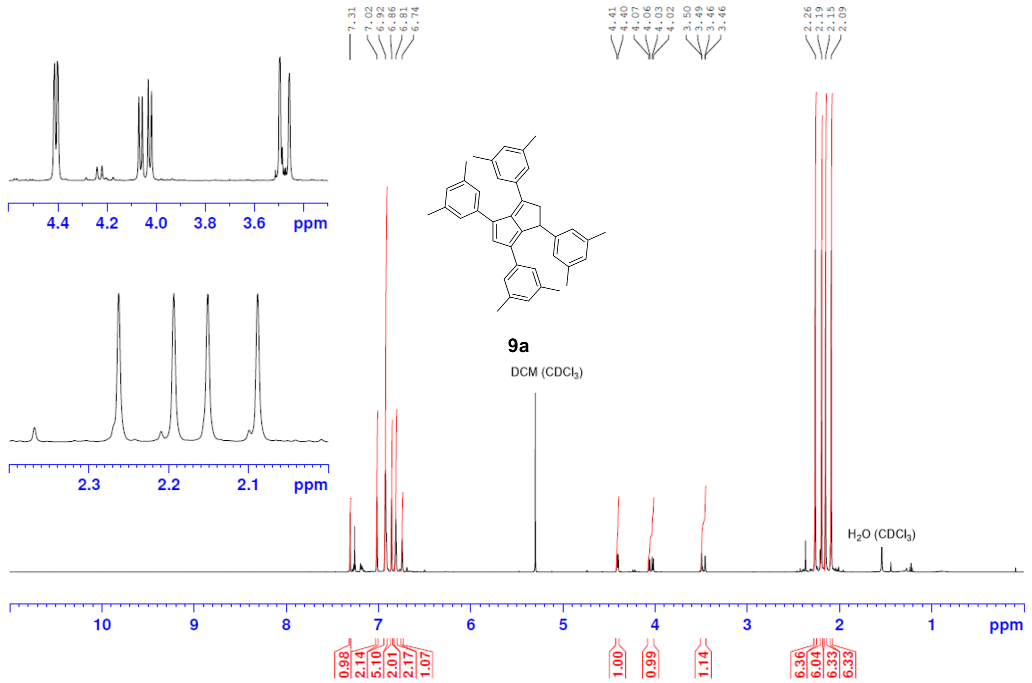

Figure S16: ^{1}H NMR of 1,3,4,6-tetrakis(3,5-dimethylphenyl)-1,2-dihydropentalene **9a**

(500 MHz, $CDCl_3$, 298 K)

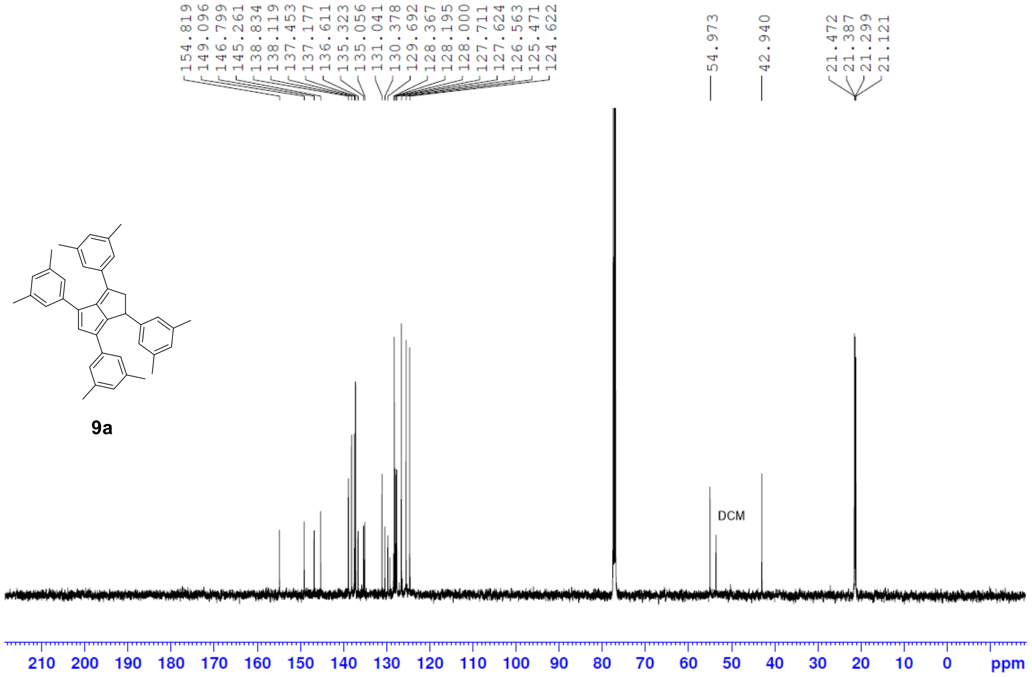

Figure S17: $^{13}C\{^1H\}$ NMR of 1,3,4,6-tetrakis(3,5-dimethylphenyl)-1,2-dihydropentalene **9a**

(125 MHz, $CDCl_3$, 298 K)

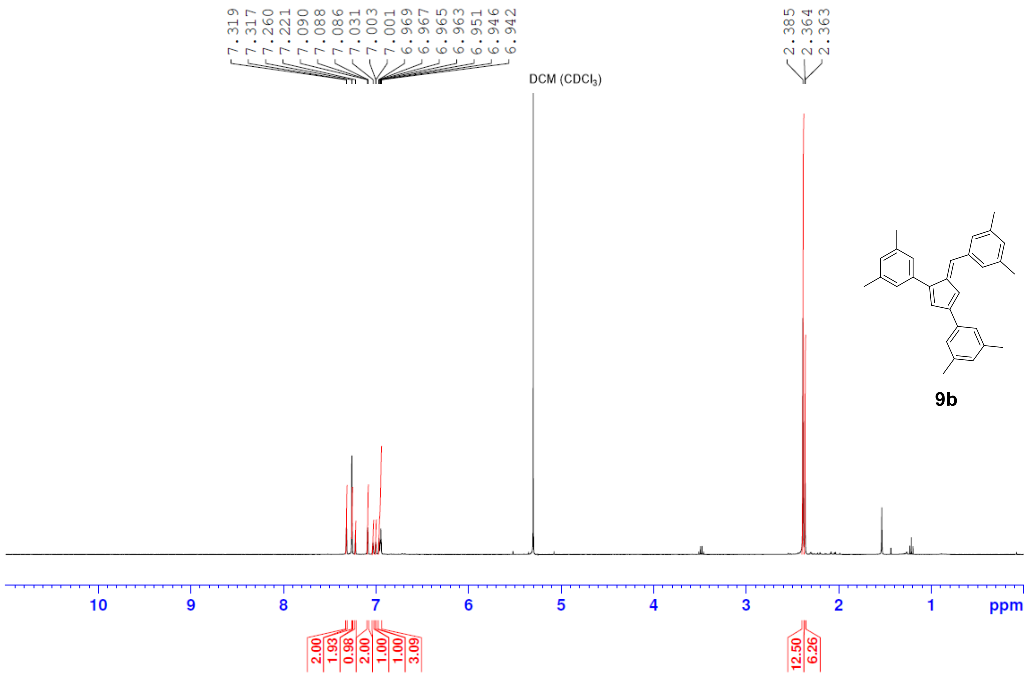

Figure S18: ^{1}H NMR of 1,3,6-tris(3,5-dimethylphenyl)-fulvene **9b** (400 MHz, $CDCl_3$, 298 K)

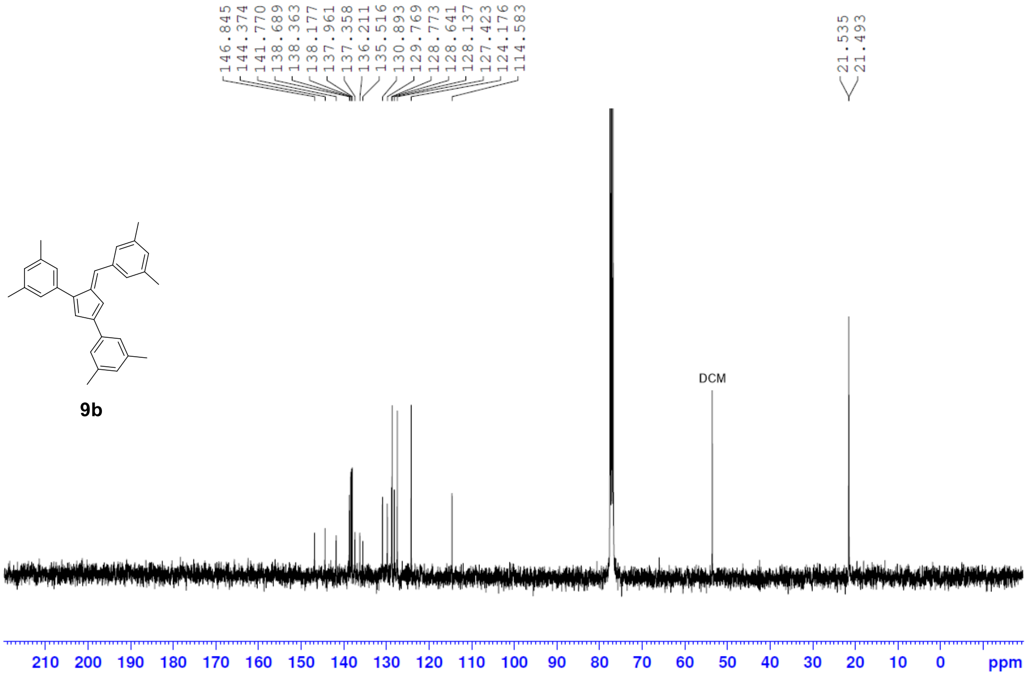

Figure S19: ^{13}C{^{1}H} NMR of 1,3,6-tris(3,5-dimethylphenyl)-fulvene **9b** (100 MHz, $CDCl_3$, 298 K)

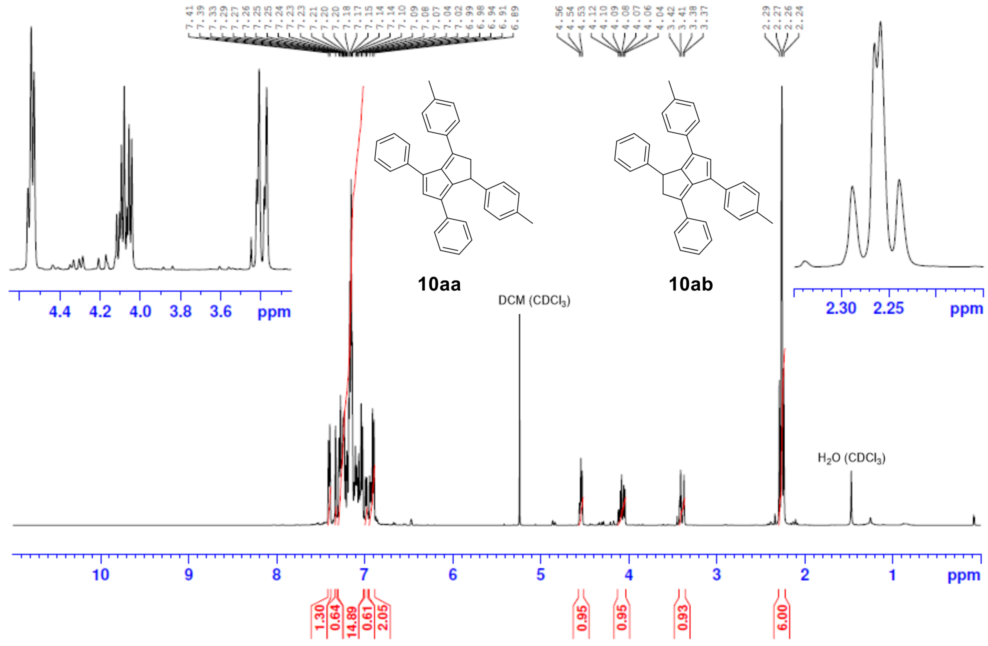

Figure S20: ^{1}H NMR of 4,6-Diphenyl-1,3-di-*p*-tolyl-1,2-dihydropentalene (**10aa**) and 1,3-diphenyl-4,6-di-*p*-tolyl-1,2-dihydropentalene (**10ab**) (500 MHz, $CDCl_3$, 298 K)

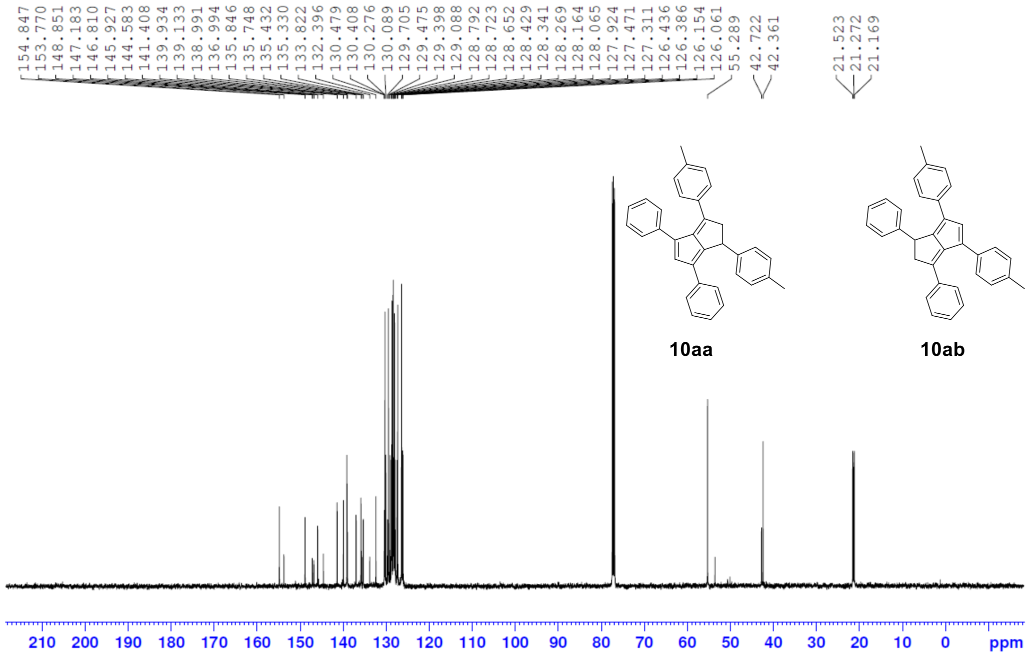

Figure S21: $^{13}C\{^{1}H\}$ NMR of 4,6-Diphenyl-1,3-di-*p*-tolyl-1,2-dihydropentalene (**10aa**) and 1,3-diphenyl-4,6-di-*p*-tolyl-1,2-dihydropentalene (**10ab**) (125 MHz, $CDCl_3$, 298 K)

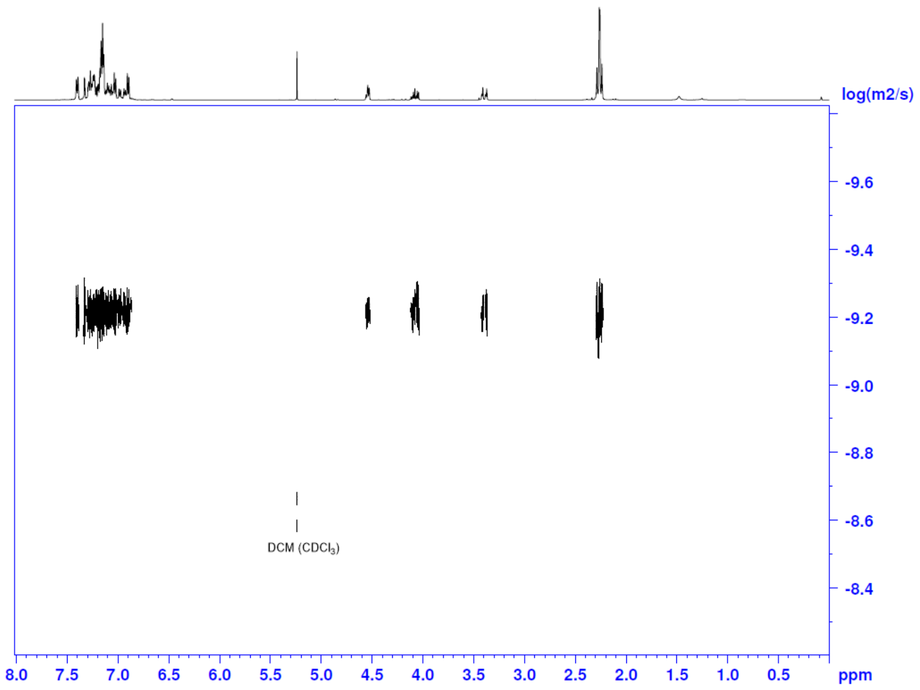

Figure S22: ^{1}H DOSY of 4,6-Diphenyl-1,3-di-p-tolyl-1,2-dihydropentalene (**10aa**) and 1,3-diphenyl-4,6-di-p-tolyl-1,2-dihydropentalene (**10ab**) (500 MHz, $CDCl_3$, 298 K)

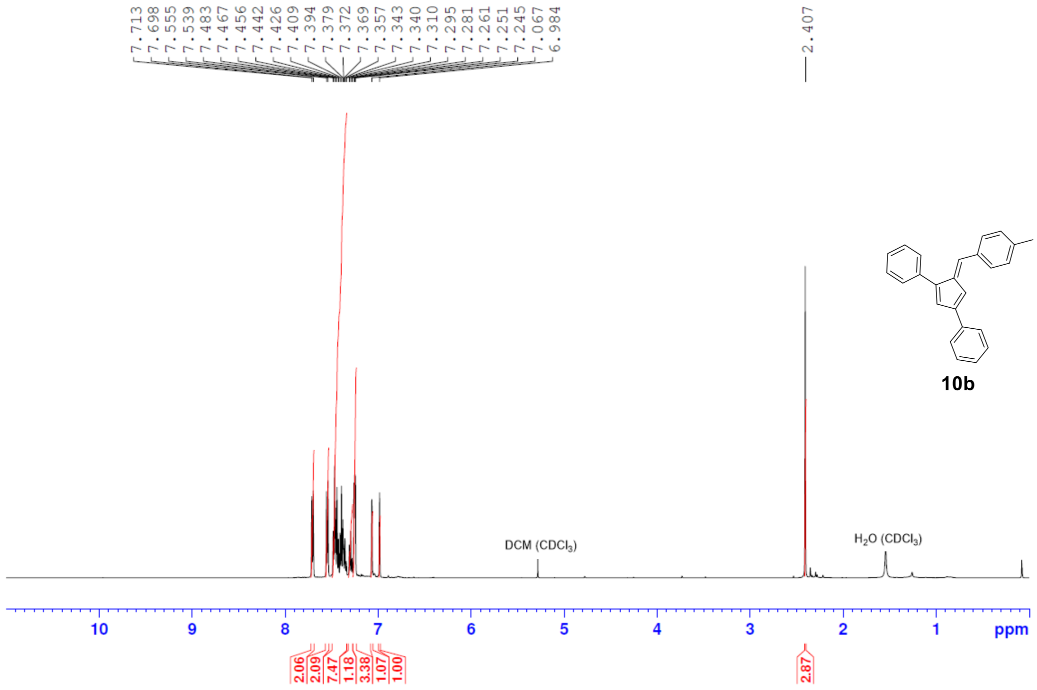

Figure S23: ^{1}H NMR of 1,3-diphenyl-6-*p*-toluylfulvene **10b** (500 MHz, $CDCl_3$, 298 K)

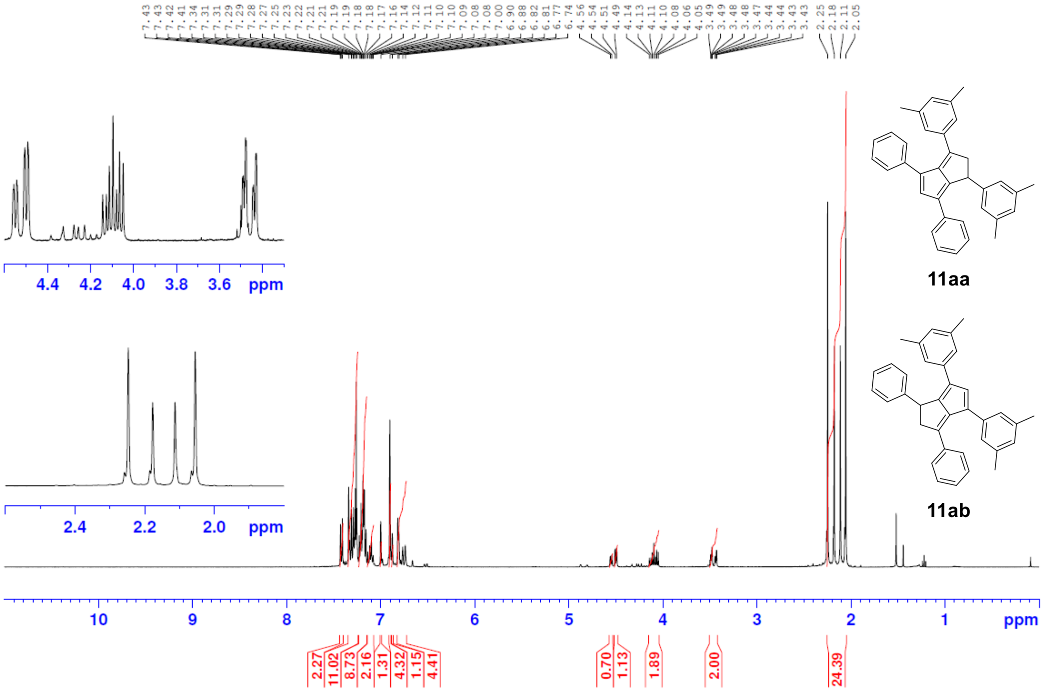

Figure S24: ^{1}H NMR of 1,3-bis(3,5-dimethylphenyl)-4,6-diphenyl-1,2-dihydropentalene (**11aa**) and 4,6-bis(3,5-dimethylphenyl)-1,3-diphenyl-1,2-dihydropentalene (**11ab**) (400 MHz, $CDCl_3$, 298 K)

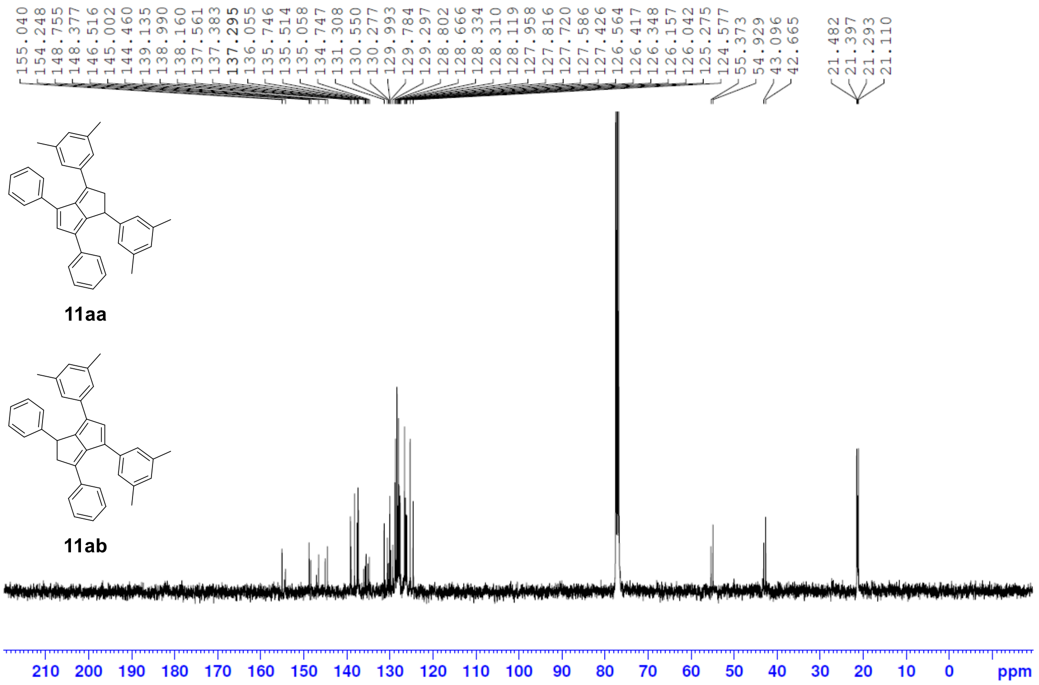

Figure S25: $^{13}C\{^{1}H\}$ NMR of 1,3-bis(3,5-dimethylphenyl)-4,6-diphenyl-1,2-dihydropentalene (**11aa**) and 4,6-bis(3,5-dimethylphenyl)-1,3-diphenyl-1,2-dihydropentalene (**11ab**) (100 MHz, $CDCl_3$, 298 K)

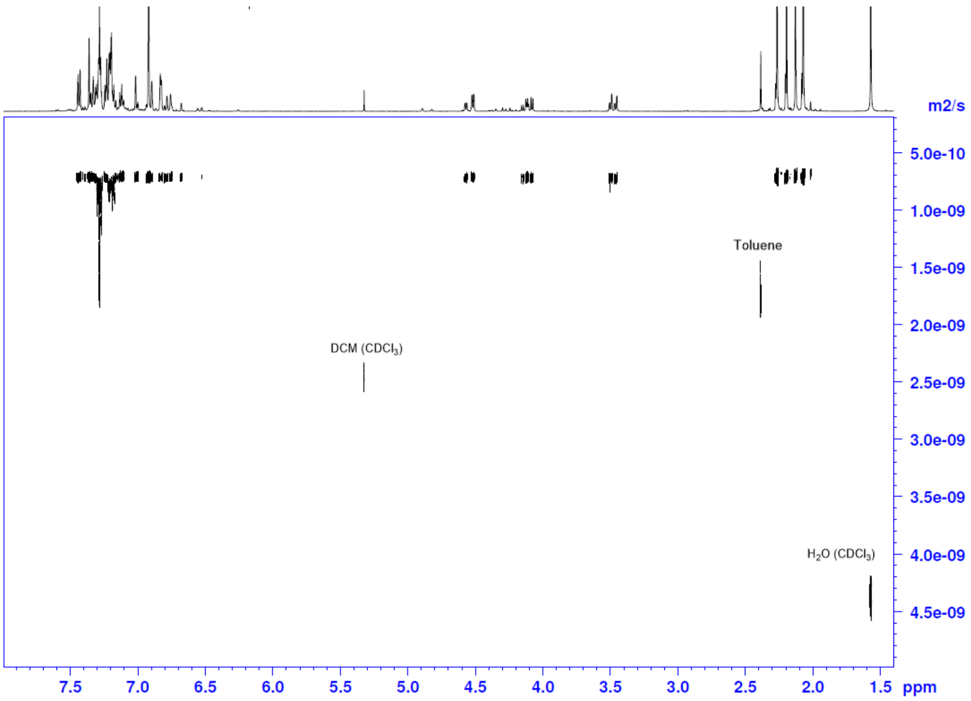

Figure S26: ^{1}H NMR of 1,3-bis(3,5-dimethylphenyl)-4,6-diphenyl-1,2-dihydropentalene (**11aa**) and 4,6-bis(3,5-dimethylphenyl)-1,3-diphenyl-1,2-dihydropentalene (**11ab**) (500 MHz, $CDCl_3$, 298 K)

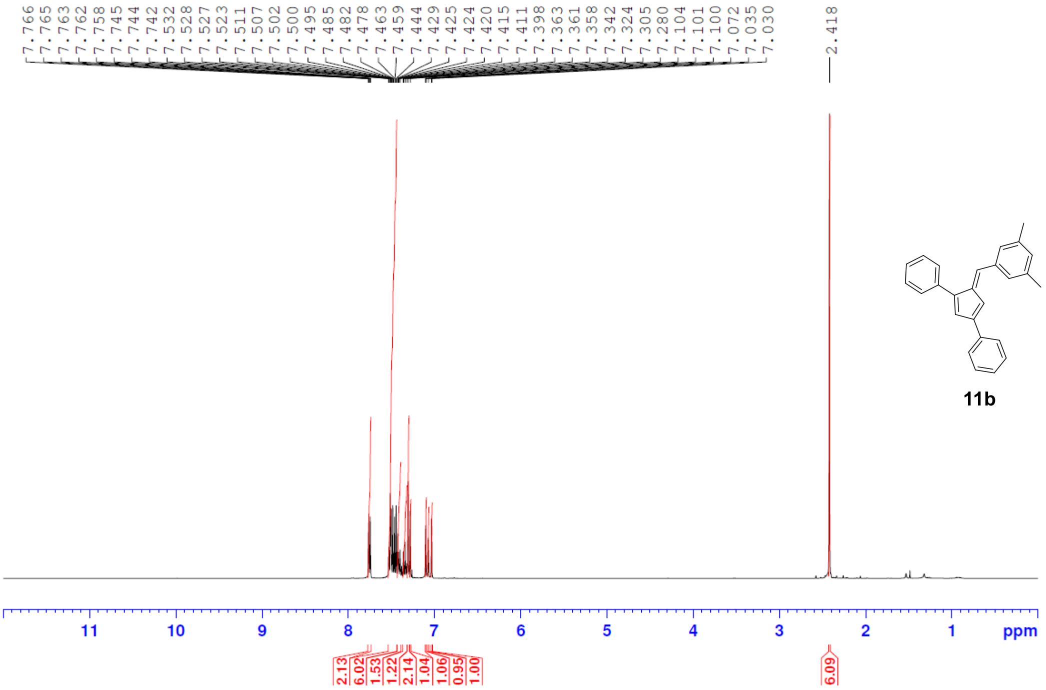

Figure S27: ^{1}H NMR of 1,3-diphenyl-6-(3,5-dimethylphenyl)fulvene (**11b**) (400 MHz, $CDCl_3$, 298 K)

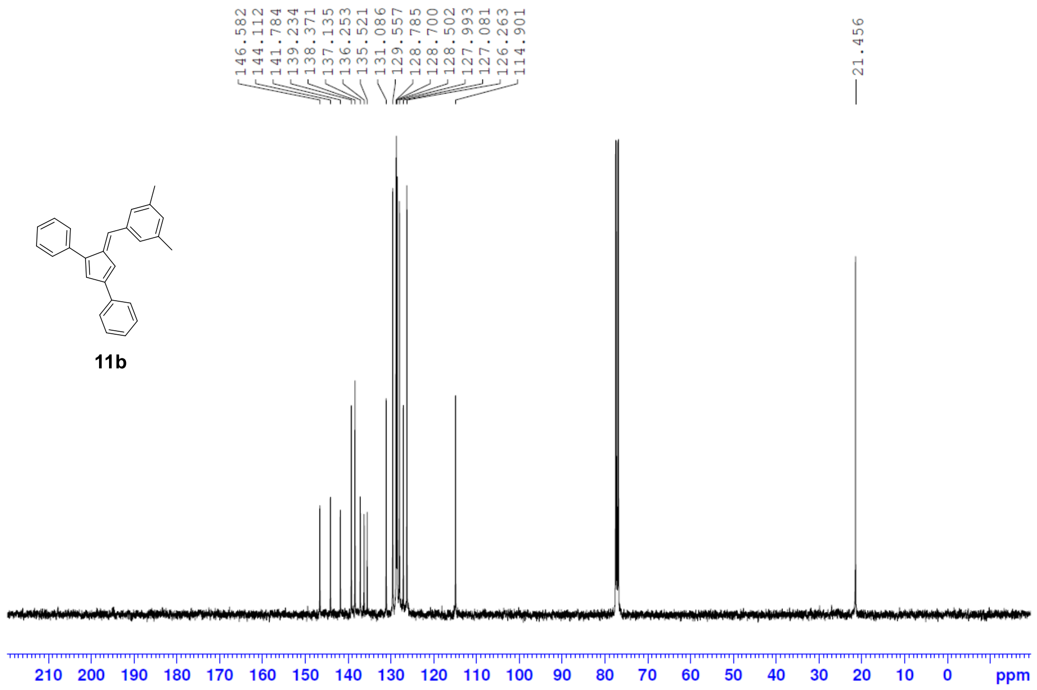

Figure S28: $^{13}C\{^{1}H\}$ NMR of 1,3-diphenyl-6-(3,5-dimethylphenyl)fulvene (**11b**) (100 MHz, $CDCl_3$, 298K)

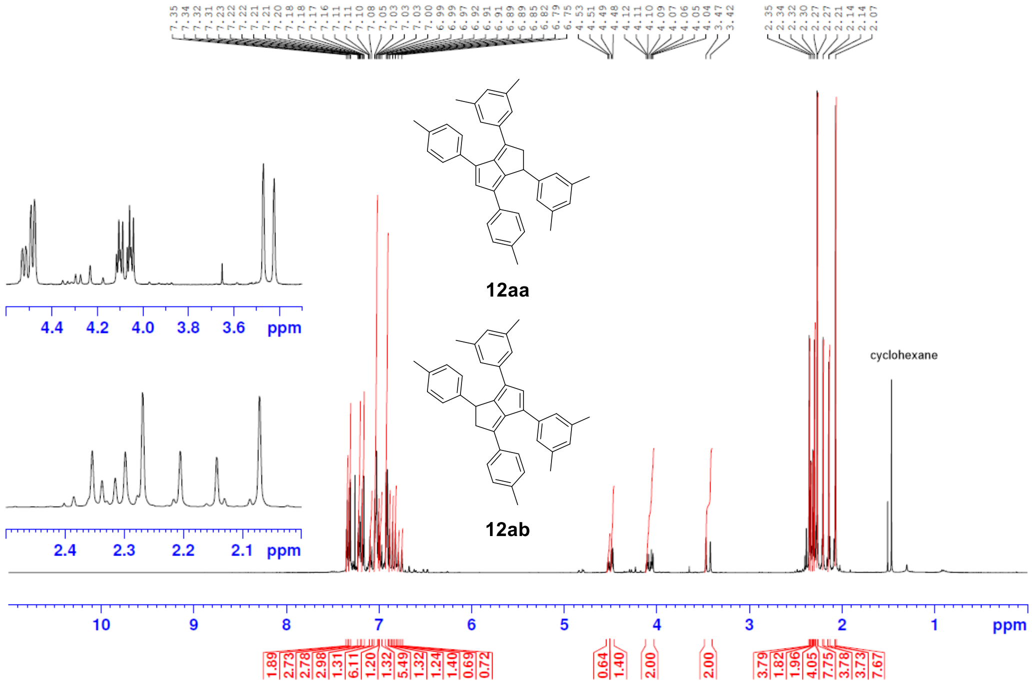

Figure S29: ^{1}H NMR of 1,3-bis(3,5-dimethylphenyl)-4,6-di-*p*-tolyl-1,2-dihydropentalene (**12aa**) and 4,6-bis(3,5-dimethylphenyl)-1,3-di-*p*-tolyl-1,2-dihydropentalene (**12ab**) (400 MHz, $CDCl_3$, 298 K)

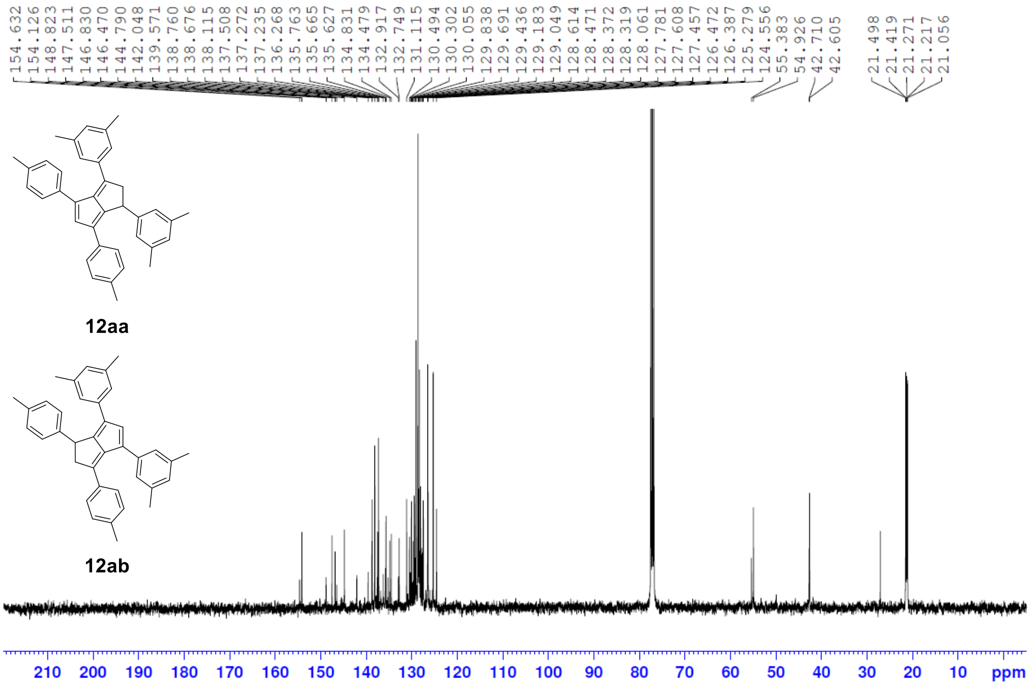

Figure S30: $^{13}C\{^{1}H\}$ NMR of 1,3-bis(3,5-dimethylphenyl)-4,6-di-*p*-tolyl-1,2-dihydropentalene (**12aa**) and 4,6-bis(3,5-dimethylphenyl)-1,3-di-*p*-tolyl-1,2-dihydropentalene (**12ab**) (100 MHz, $CDCl_3$, 298 K)

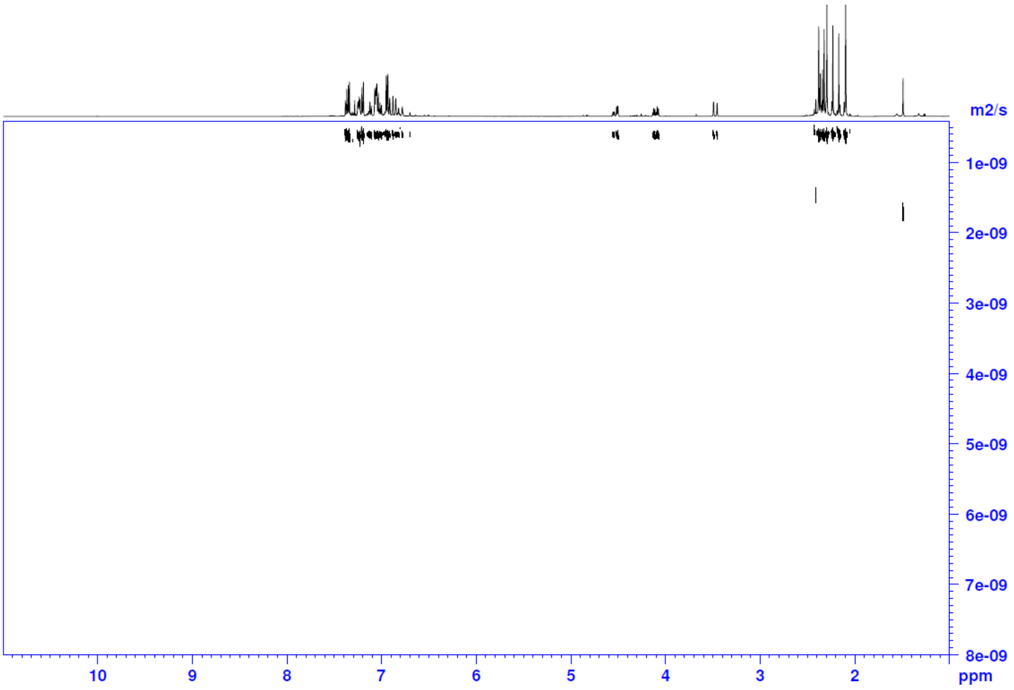

Figure S31: ^{1}H DOSY of 1,3-bis(3,5-dimethylphenyl)-4,6-di-*p*-tolyl-1,2-dihydropentalene (**12aa**) and 4,6-bis(3,5-dimethylphenyl)-1,3-di-*p*-tolyl-1,2-dihydropentalene (**12ab**) (500 MHz, $CDCl_3$, 298 K)

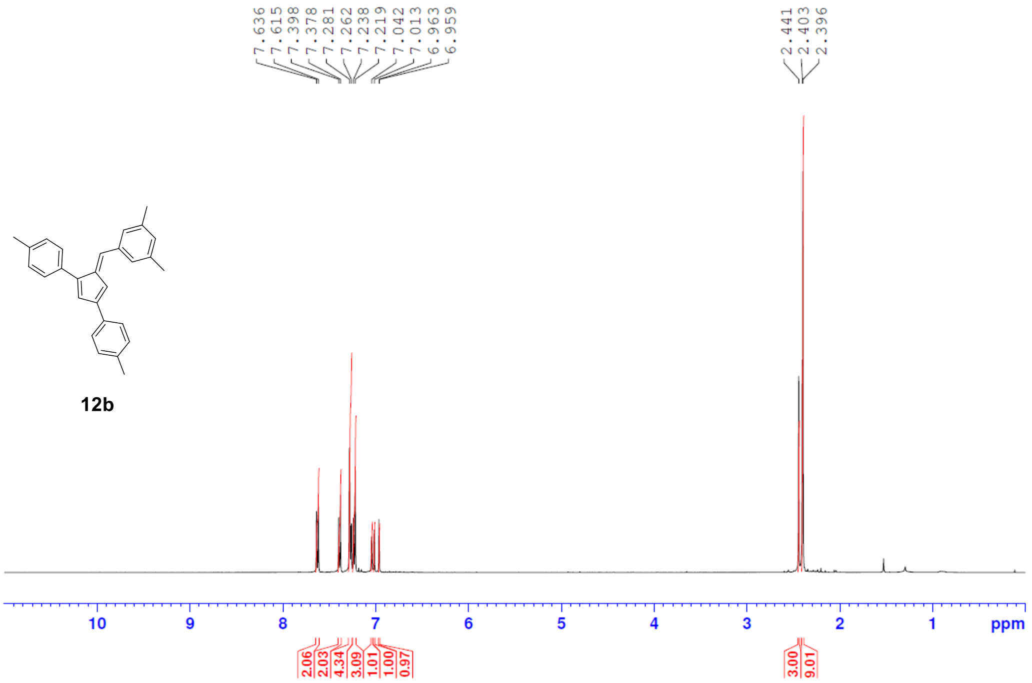

Figure S32: ^{1}H NMR of 1,3-di-*p*-toluyl-6-(3,5-dimethylphenyl)fulvene **12b** (400 MHz, $CDCl_3$, 298 K)

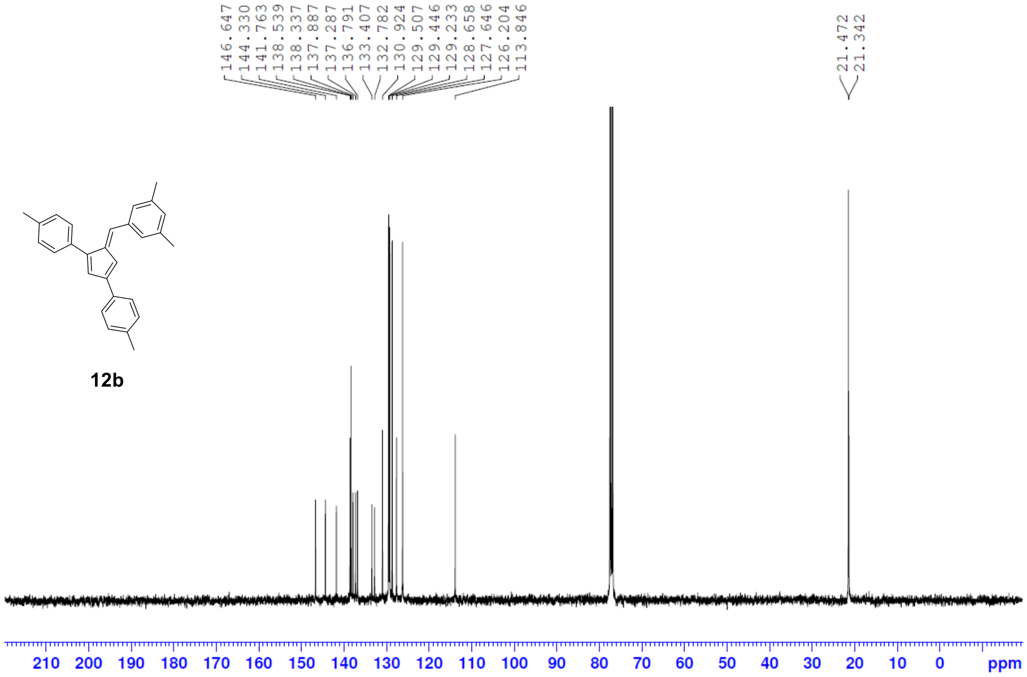

Figure S33: $^{13}C\{^{1}H\}$ NMR of 1,3-di-*p*-toluyl-6-(3,5-dimethylphenyl)fulvene **12b**

(100 MHz, $CDCl_3$, 298 K)

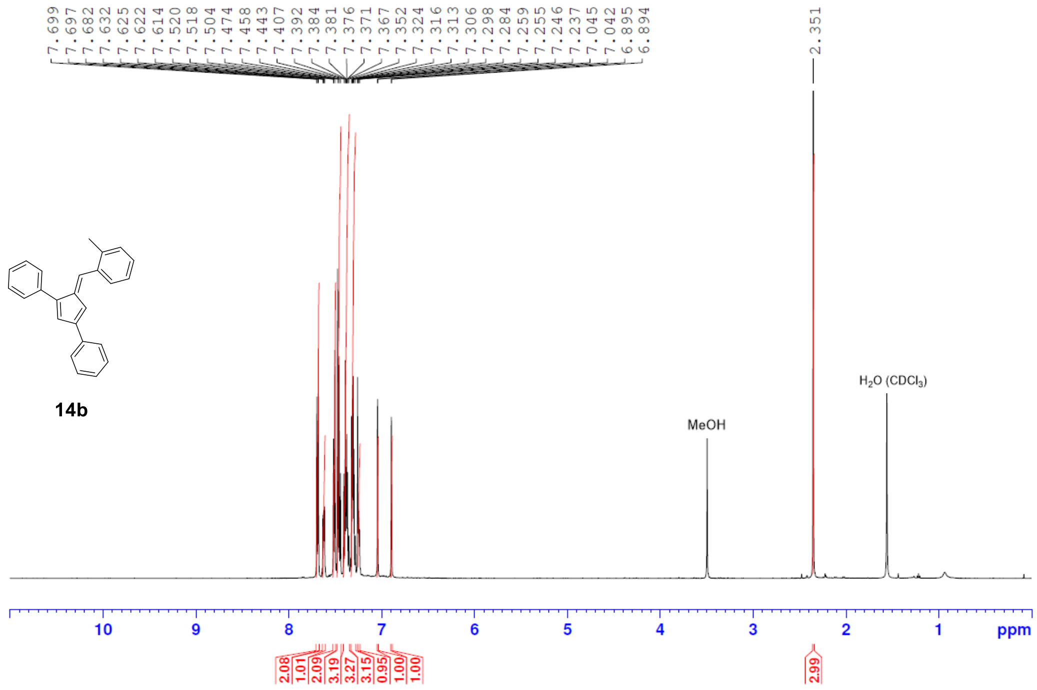

Figure S34: ^{1}H NMR of 1,3-diphenyl-6-*o*-toluylfulvene **14b** (500 MHz, $CDCl_3$, 298 K)

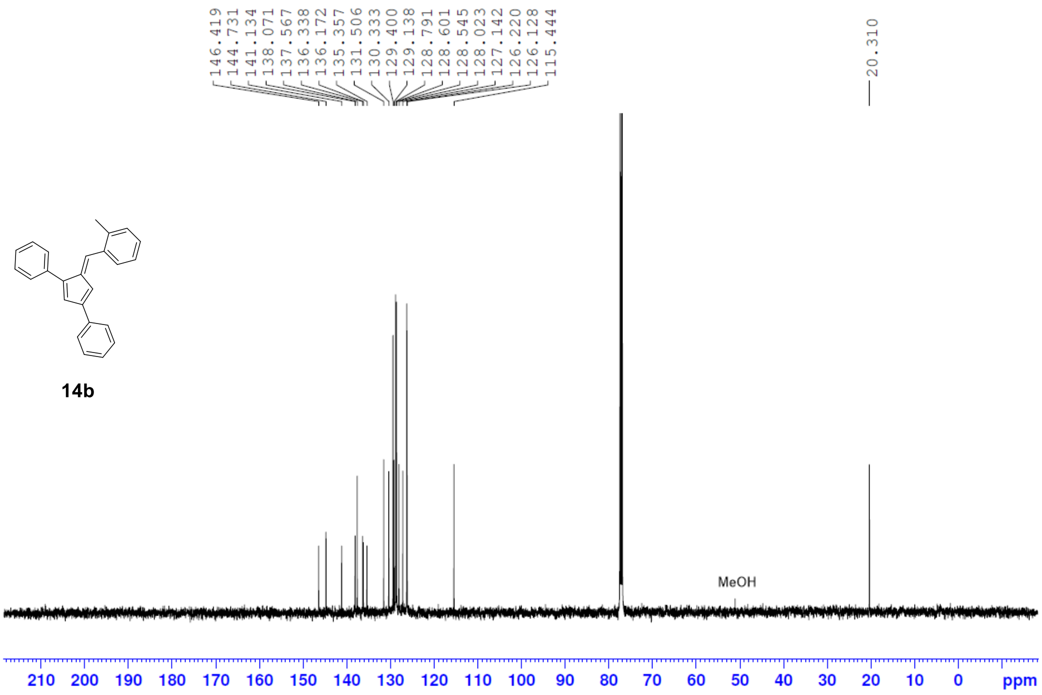

Figure S35: ^{13}C{^{1}H} NMR of 1,3-diphenyl-6-*o*-toluylfulvene **14b**

(125 MHz, $CDCl_3$, 298 K)

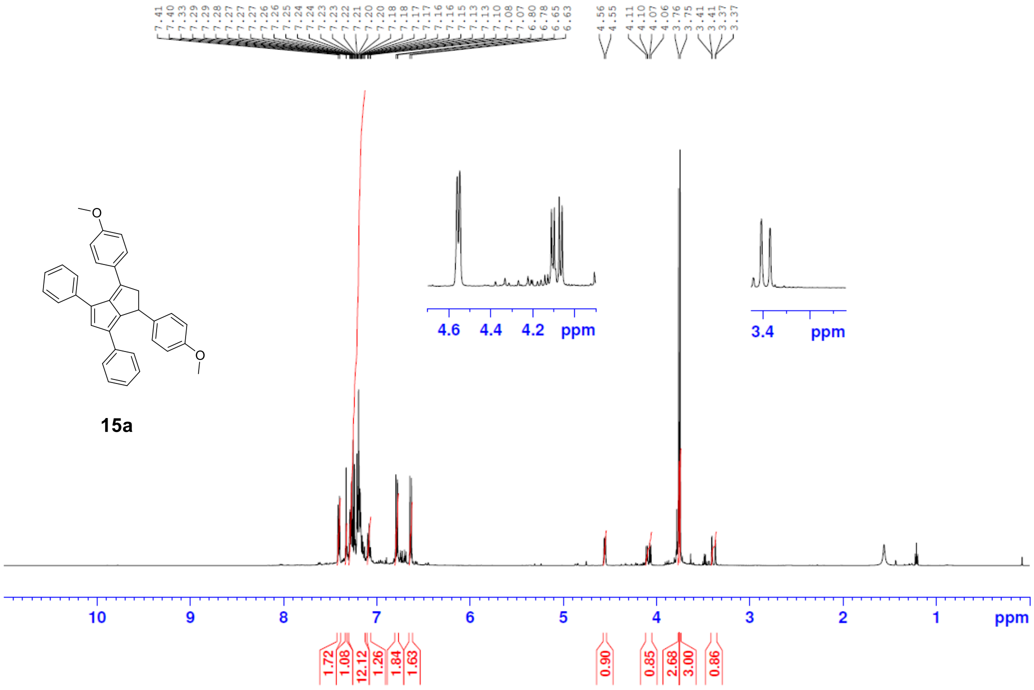

Figure S36: ^{1}H NMR of 1,3-bis(4-methoxyphenyl)-4,6-diphenyl-1,2-dihydropentalene **15a**

(500 MHz, $CDCl_3$, 298 K)

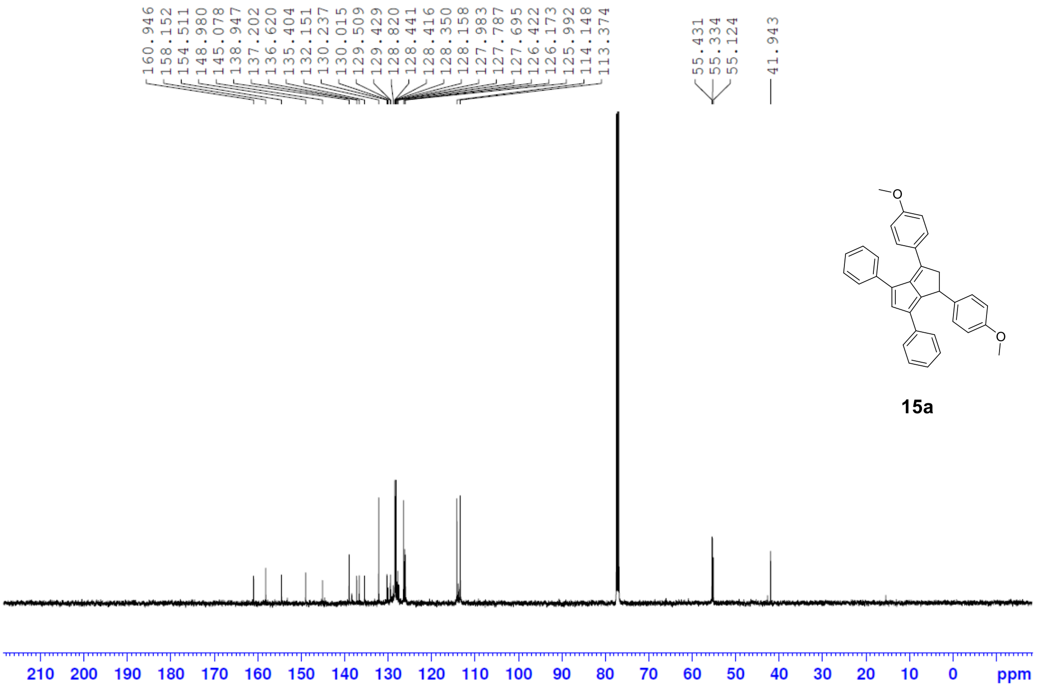

Figure S37: $^{13}C\{^{1}H\}$ NMR of 1,3-bis(4-methoxyphenyl)-4,6-diphenyl-1,2-dihydropentalene **15a**

(125 MHz, $CDCl_3$, 298 K)

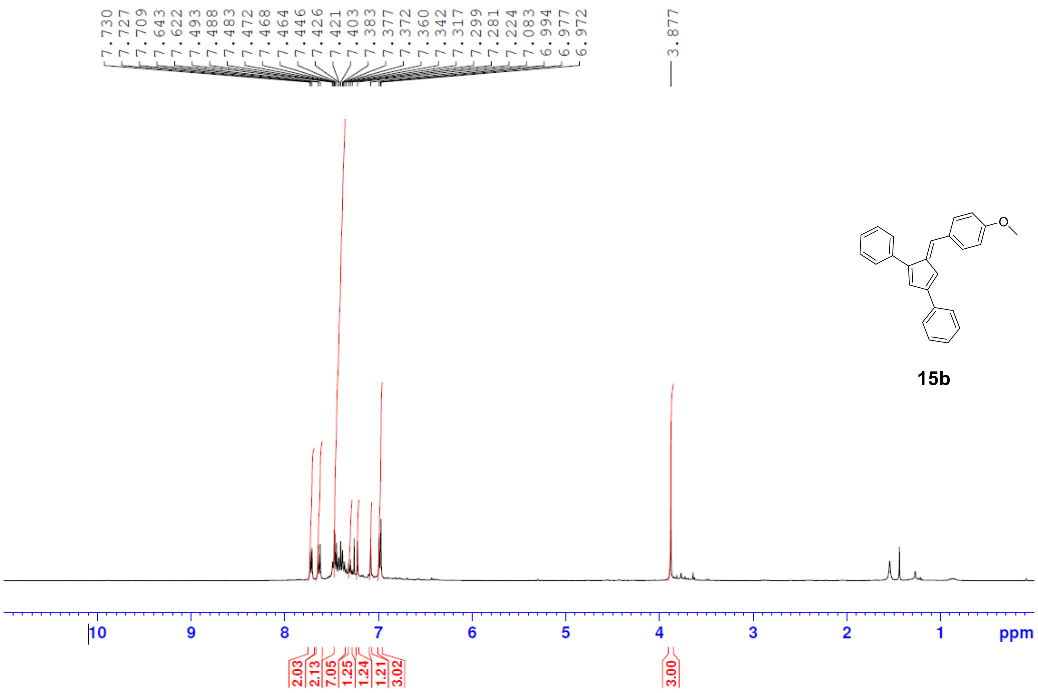

Figure S38: ^{1}H NMR of 1,3-diphenyl-6-(4-methoxyphenyl)fulvene **15b** (500 MHz, $CDCl_3$, 298 K)

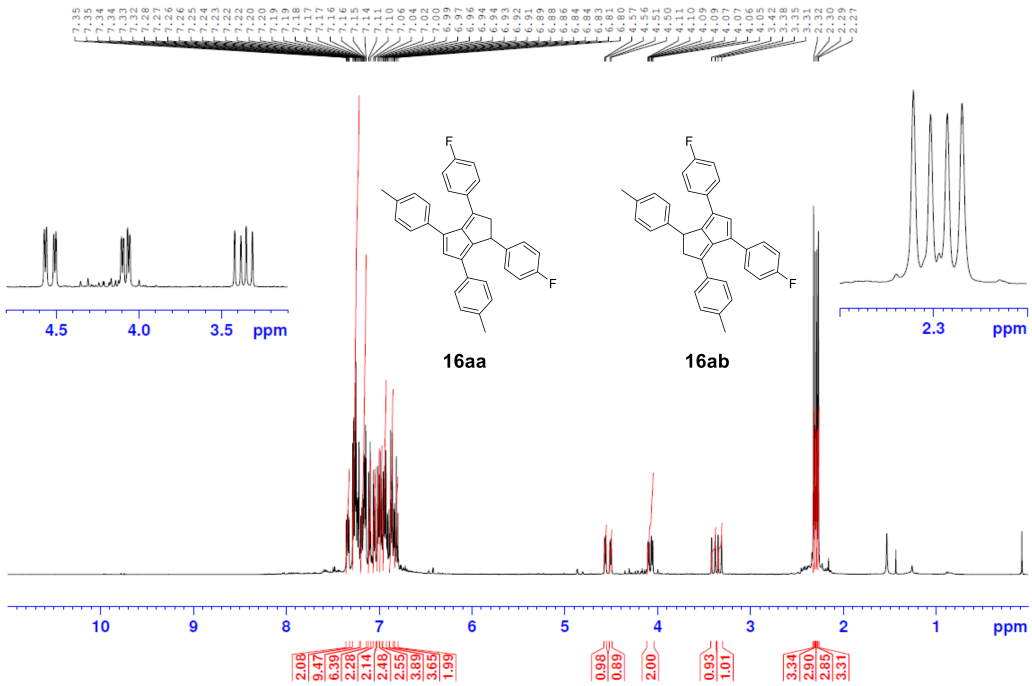

Figure S39: ^{1}H NMR of 1,3-bis(4-fluorophenyl)-4,6-di-*p*-tolyl-1,2-dihydropentalene (**16aa**) and 4,6-bis(4-fluorophenyl)-1,3-di-*p*-tolyl-1,2-dihydropentalene (**16ab**) (500 MHz, $CDCl_3$, 298 K)

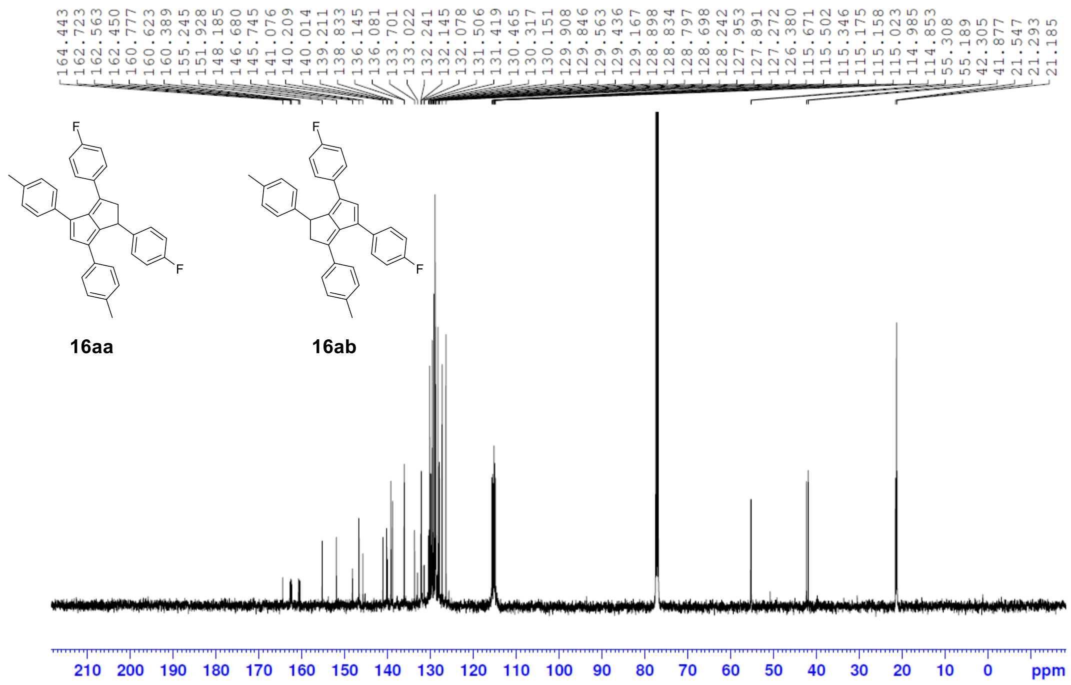

Figure S40: $^{13}C\{^{1}H\}$ NMR of 1,3-bis(4-fluorophenyl)-4,6-di-*p*-tolyl-1,2-dihydropentalene (**16aa**) and 4,6-bis(4-fluorophenyl)-1,3-di-*p*-tolyl-1,2-dihydropentalene (**16ab**) (125 MHz, $CDCl_3$, 298 K)

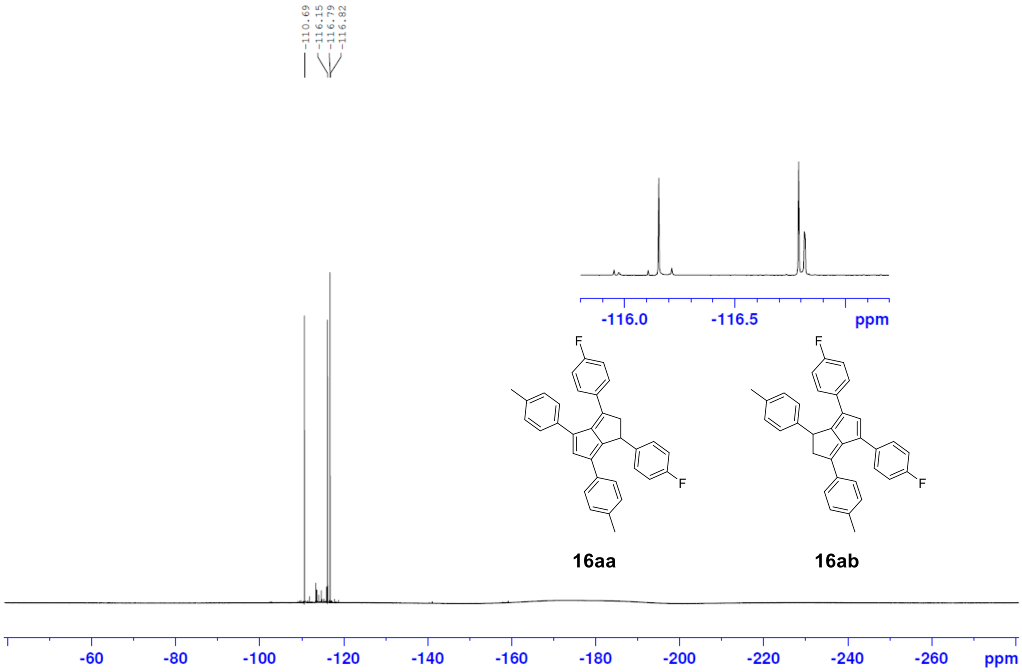

Figure S41: $^{19}F\{^{1}H\}$ NMR of 1,3-bis(4-fluorophenyl)-4,6-di-*p*-tolyl-1,2-dihydropentalene (**16aa**) and 4,6-bis(4-fluorophenyl)-1,3-di-*p*-tolyl-1,2-dihydropentalene (**16ab**) (470 MHz, $CDCl_3$, 298 K)

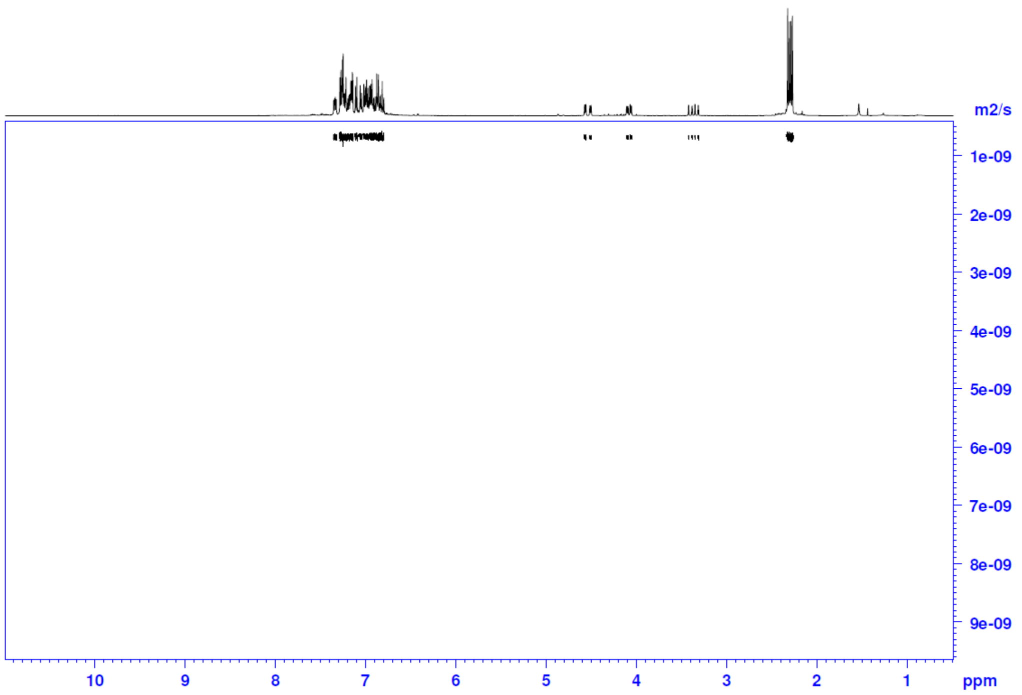

Figure S42: ^{1}H DOSY of 1,3-bis(4-fluorophenyl)-4,6-di-*p*-tolyl-1,2-dihydropentalene (**16aa**) and 4,6-bis(4-fluorophenyl)-1,3-di-*p*-tolyl-1,2-dihydropentalene (**16ab**) (500 MHz, $CDCl_3$, 298 K)

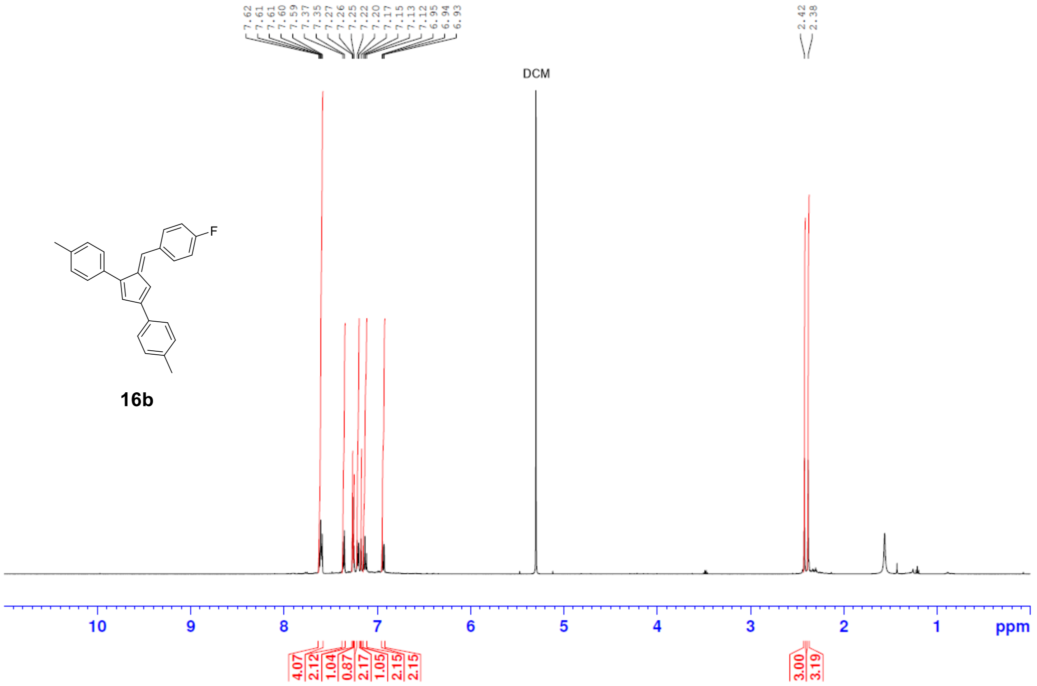

Figure S43: ^{1}H NMR of 1,3-ditoluyl-6-(4-fluorophenyl)fulvene **16b** (500 MHz, $CDCl_3$, 298 K)

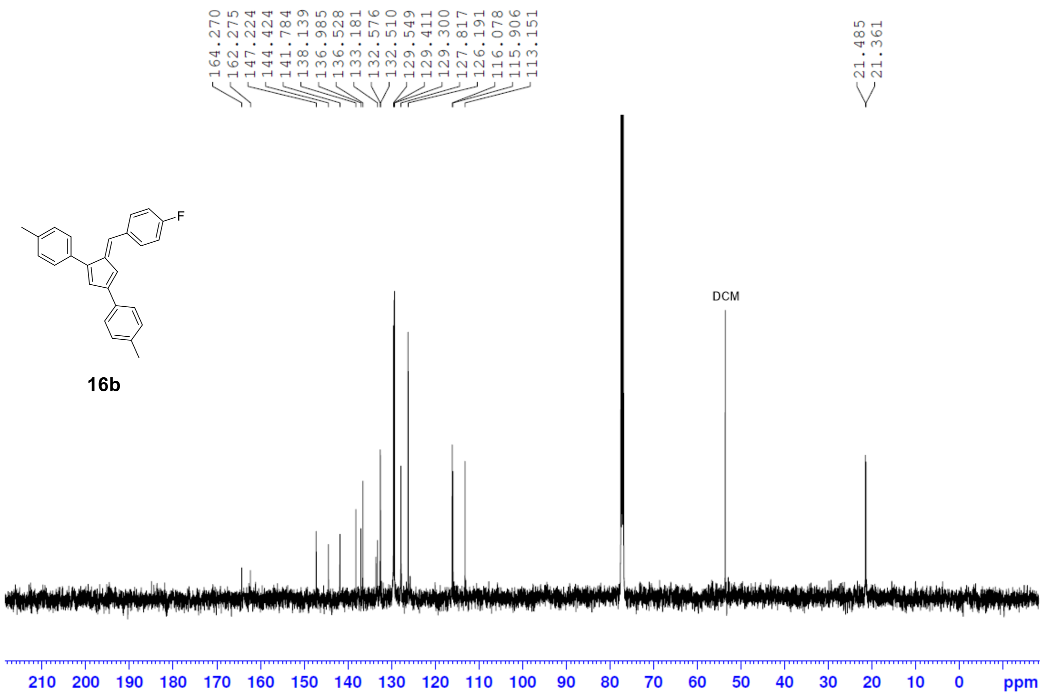

Figure S44: $^{13}C\{^{1}H\}$ NMR of 1,3-ditoluyl-6-(4-fluorophenyl)fulvene **16b** (125 MHz, $CDCl_3$, 298 K)

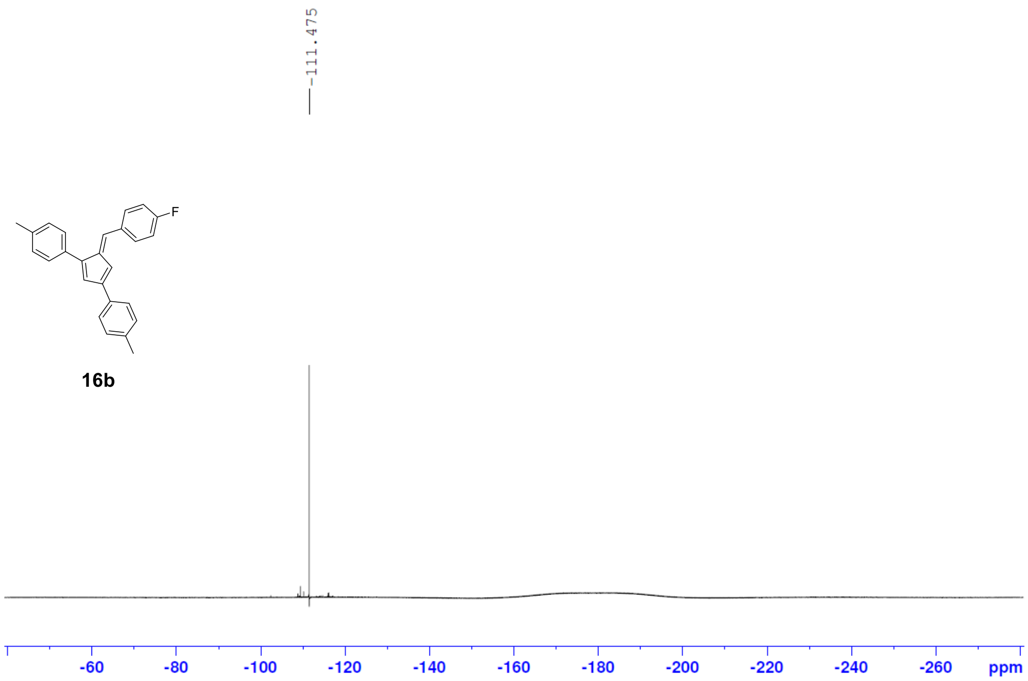

Figure S45: $^{19}F\{^{1}H\}$ NMR of 1,3-ditoluyl-6-(4-fluorophenyl)fulvene **16b** (470 MHz, $CDCl_3$, 298 K)

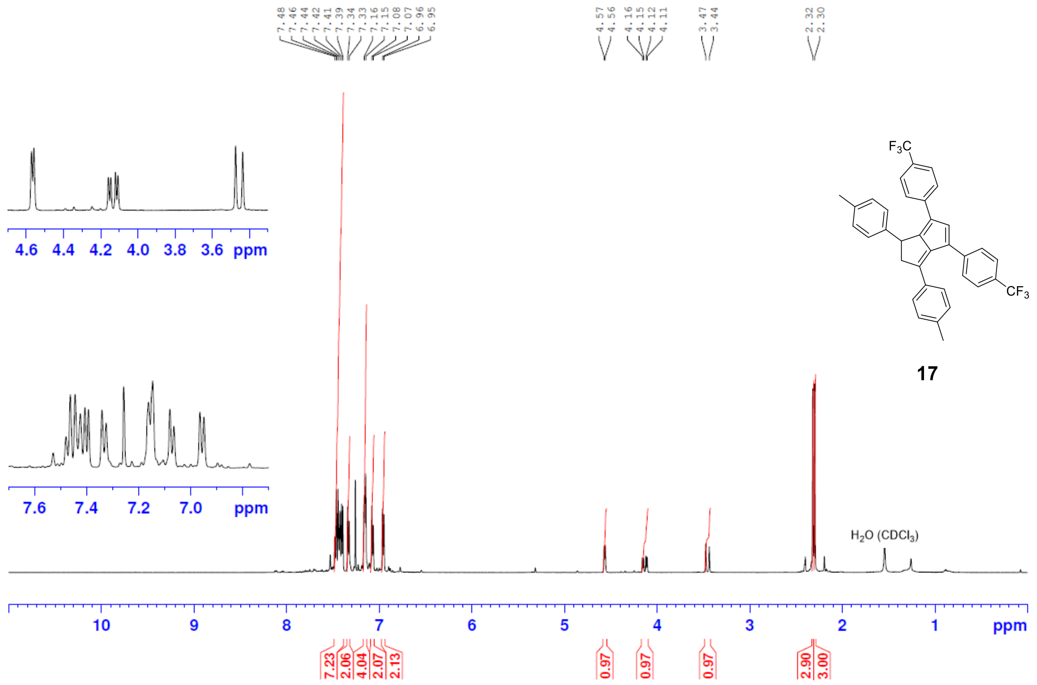

Figure S46: ^{1}H NMR of 1,3-di-*p*-tolyl-4,6-bis(4-(trifluoromethyl)phenyl)-1,2-dihydropentalene **17**

(500 MHz, $CDCl_3$, 298 K)

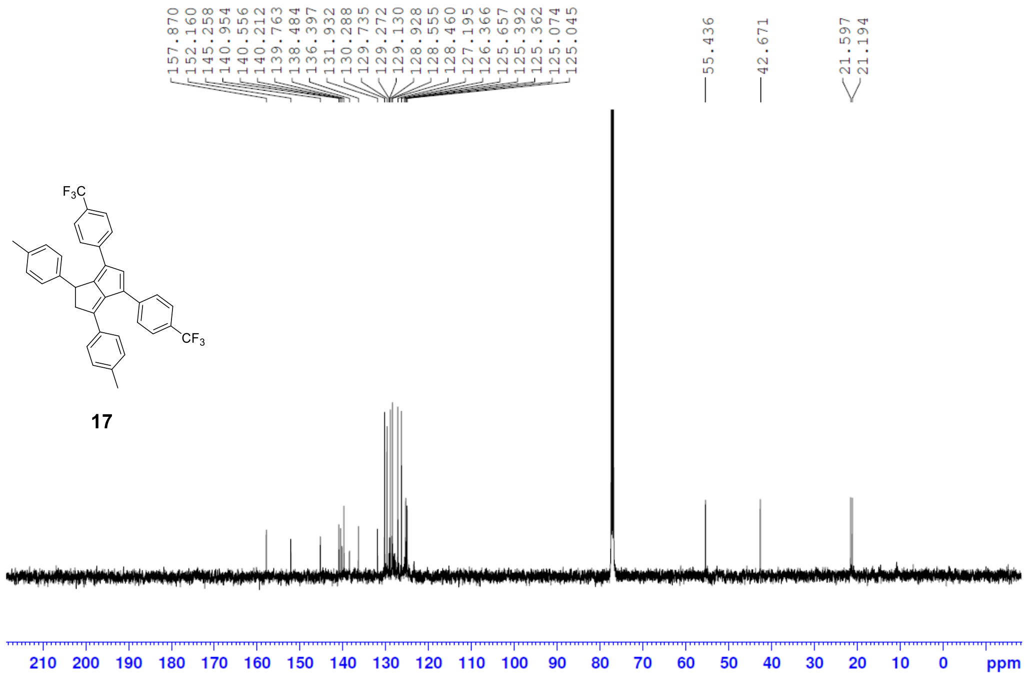

Figure S47: $^{13}C\{^{1}H\}$ NMR of 1,3-di-*p*-tolyl-4,6-bis(4-(trifluoromethyl)phenyl)-1,2-dihydropentalene **17**

(125 MHz, $CDCl_3$, 298 K)

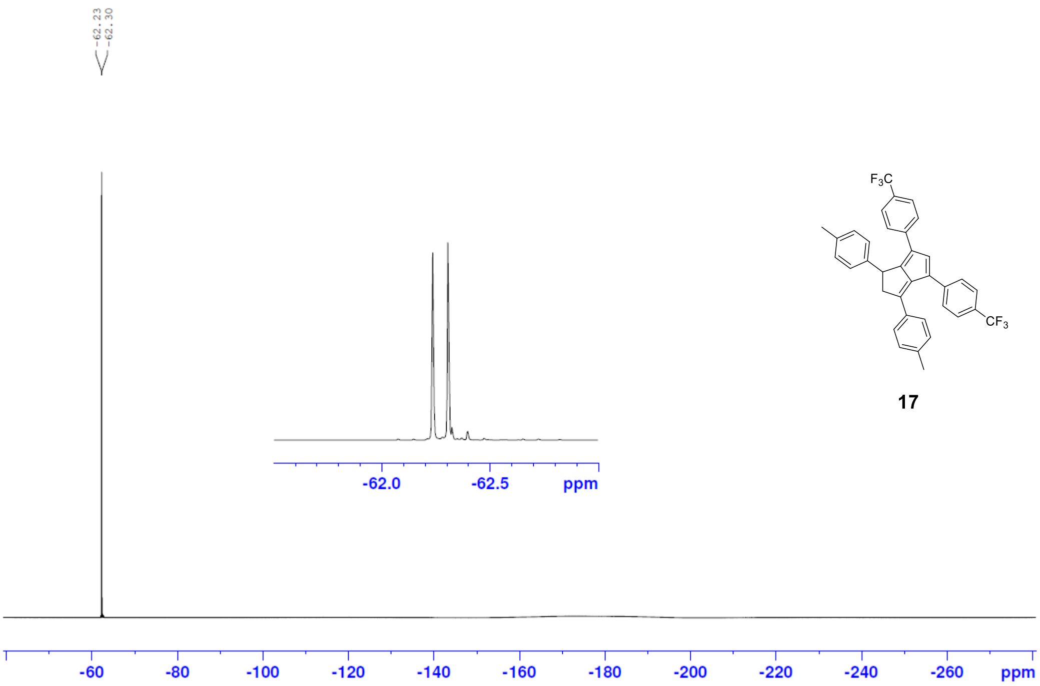

Figure S48: $^{19}F\{^{1}H\}$ NMR of 1,3-di-p-tolyl-4,6-bis(4-(trifluoromethyl)phenyl)-1,2-dihydropentalene **17**

(470 MHz, $CDCl_3$, 298 K)

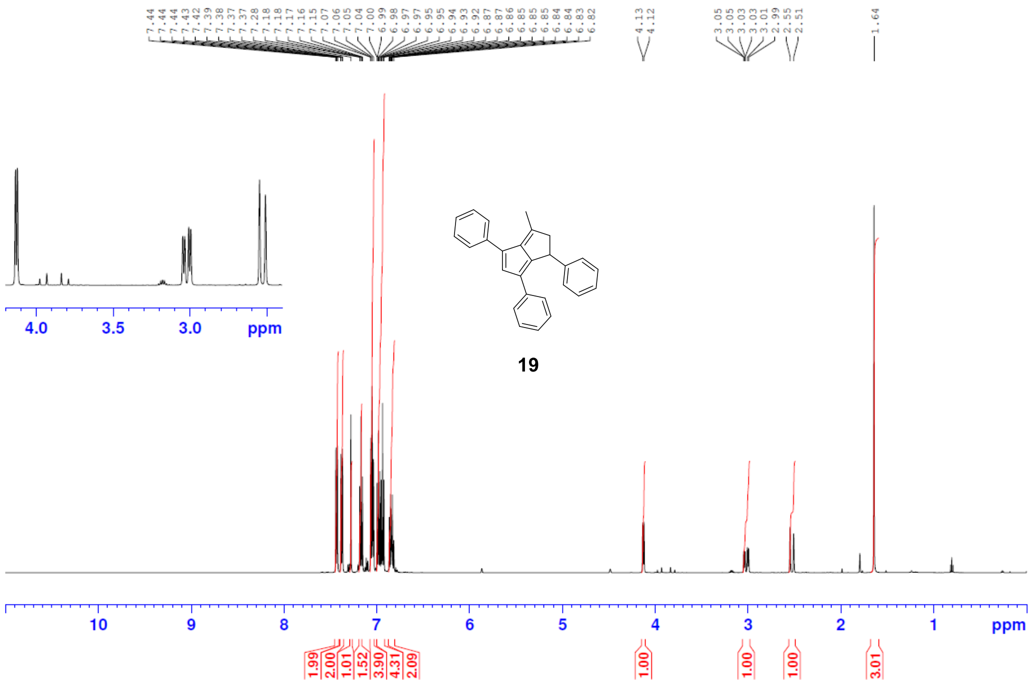

Figure S49: ^{1}H NMR of 3-methyl-1,4,6-triphenyl-1,2-dihydropentalene **19** (500 MHz, C_6D_6, 298 K)

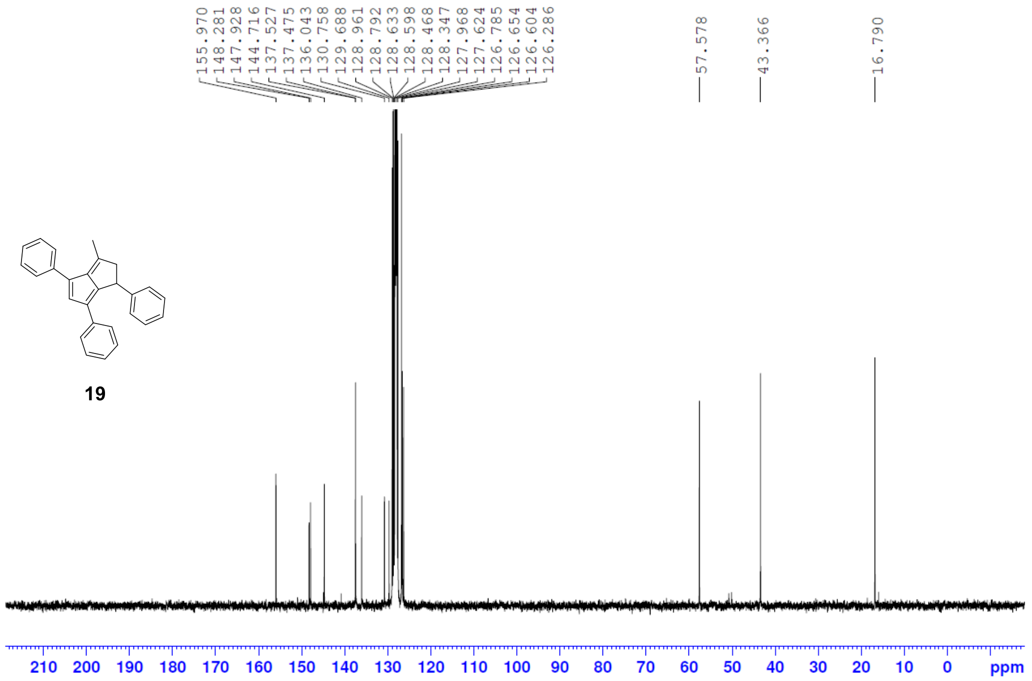

Figure S50: $^{13}C\{^{1}H\}$ NMR of 3-methyl-1,4,6-triphenyl-1,2-dihydropentalene **19** (125 MHz, C_6D_6, 298 K)

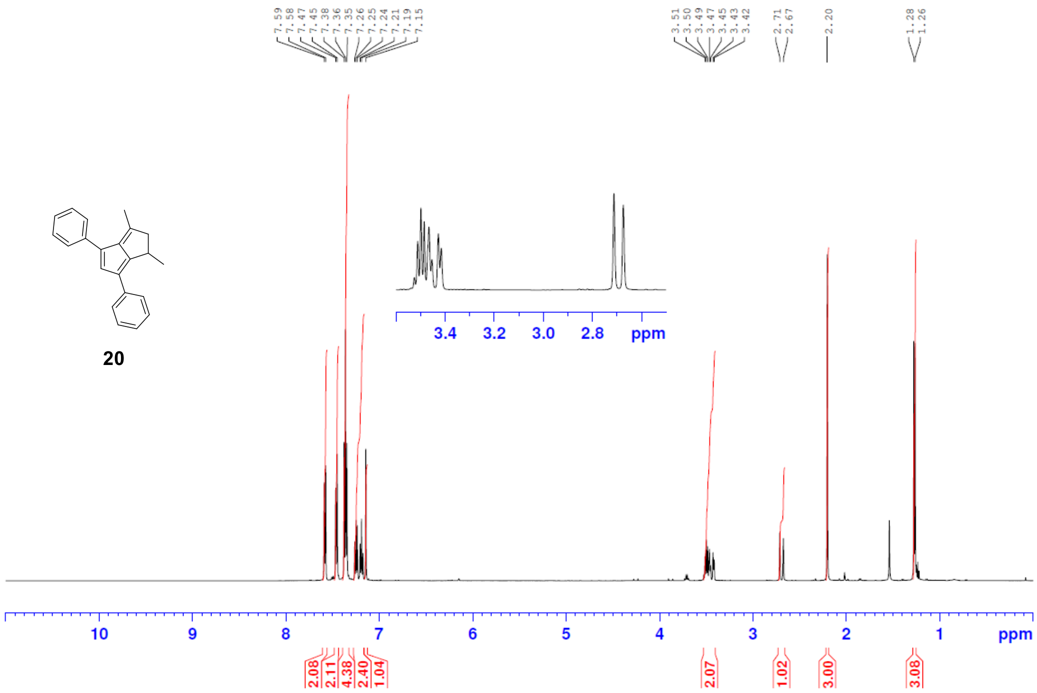

Figure S51: ^{1}H NMR of 1,3-dimethyl-4,6-diphenyl-1,2-dihydropentalene **20** (500 MHz, $CDCl_3$, 298 K)

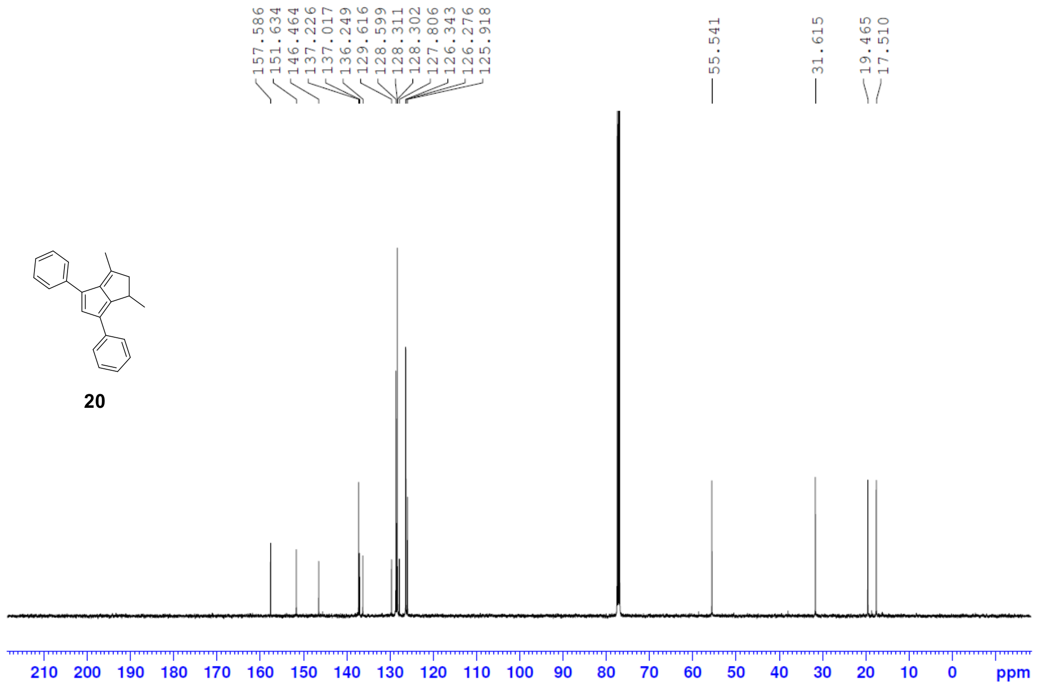

Figure S52: $^{13}C\{^{1}H\}$ NMR of 1,3-dimethyl-4,6-diphenyl-1,2-dihydropentalene **20** ($CDCl_3$, 298 K)

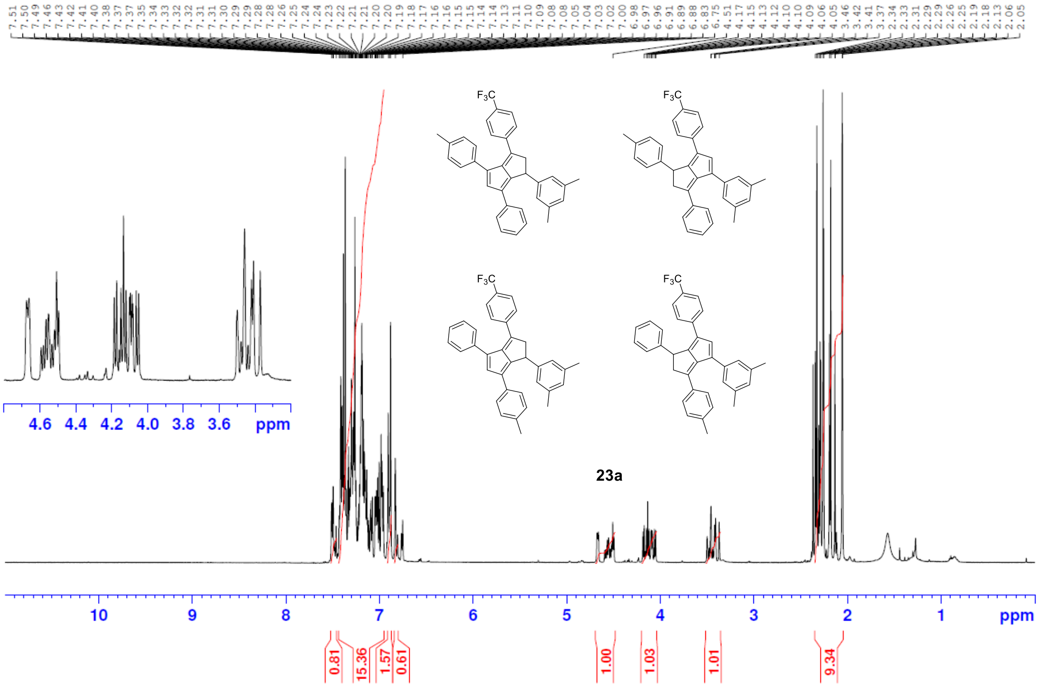

Figure S53: ^{1}H NMR of 1-(3,5-dimethylphenyl)-6-phenyl-4-(*p*-tolyl)-3-(4-(trifluoromethyl)phenyl)-1,2-dihydropentalene based isomer mixture **23a** (500 MHz, $CDCl_3$, 298 K)

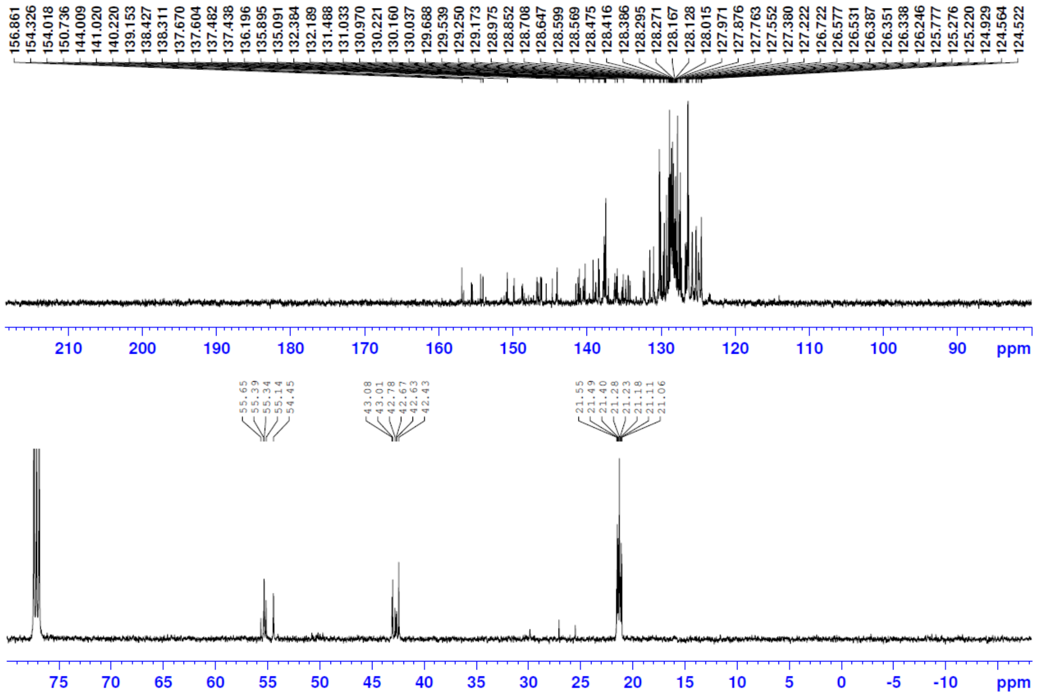

Figure S54: $^{13}C\{^1H\}$ NMR of 1-(3,5-dimethylphenyl)-6-phenyl-4-(*p*-tolyl)-3-(4-(trifluoromethyl)phenyl)-1,2-dihydropentalene based isomer mixture **23a** (125 MHz, $CDCl_3$, 298 K)

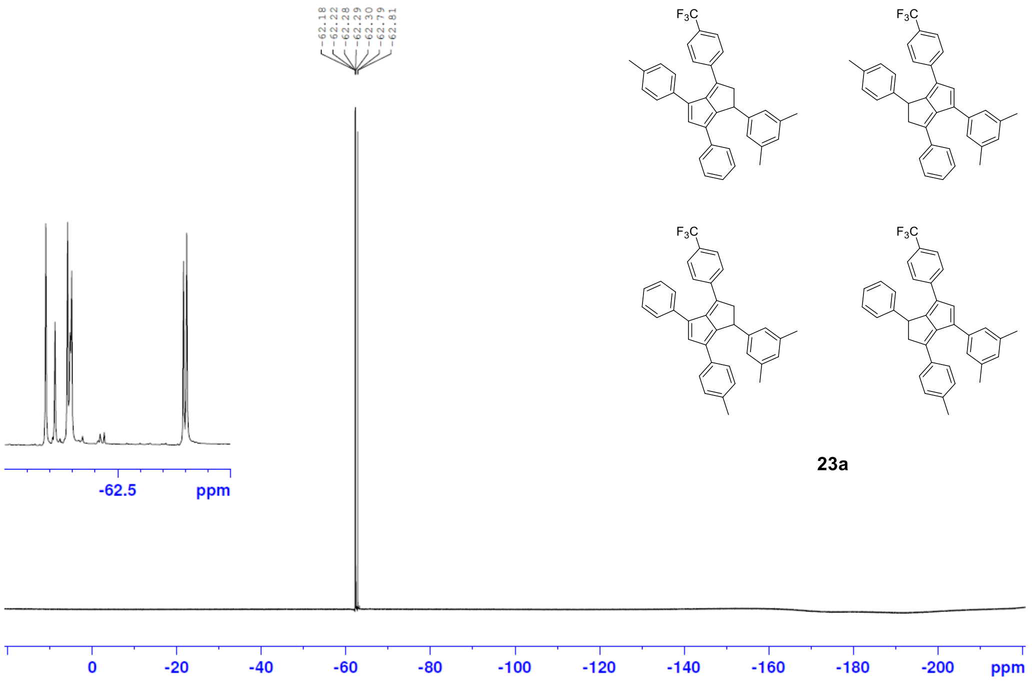

Figure S55: $^{19}F\{^1H\}$ NMR of 1-(3,5-dimethylphenyl)-6-phenyl-4-(*p*-tolyl)-3-(4-(trifluoromethyl)phenyl)-1,2-dihydropentalene based isomer mixture **23a** (470 MHz, $CDCl_3$, 298 K)

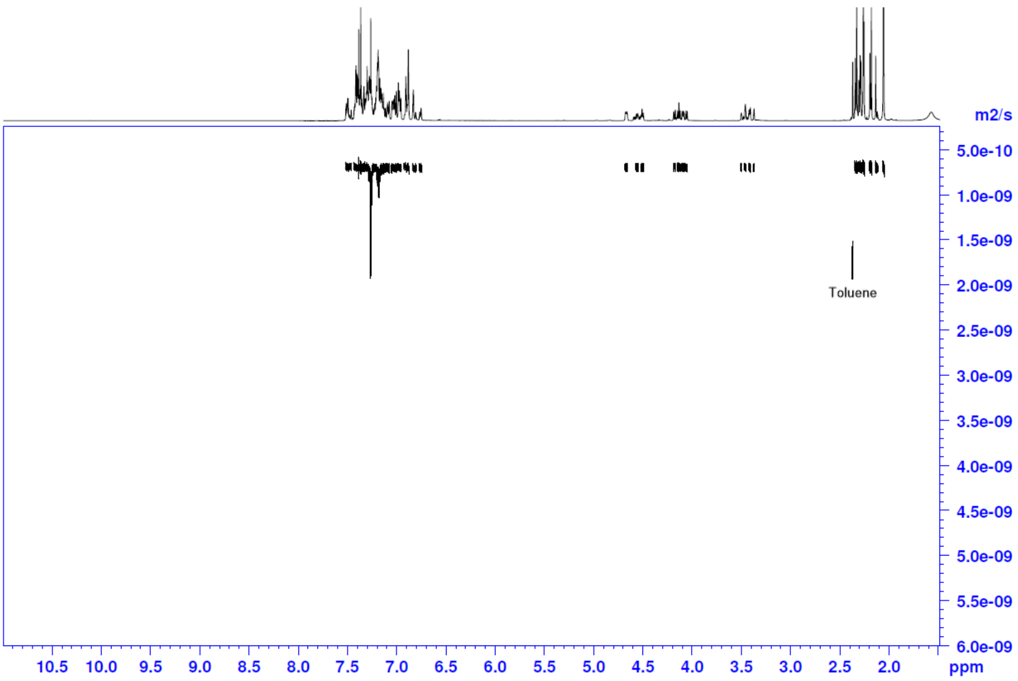

Figure S56: ^{1}H DOSY of 1-(3,5-dimethylphenyl)-6-phenyl-4-(*p*-tolyl)-3-(4-(trifluoromethyl)phenyl)-1,2-dihydropentalene based isomer mixture **23a** (500 MHz, $CDCl_3$, 298 K)

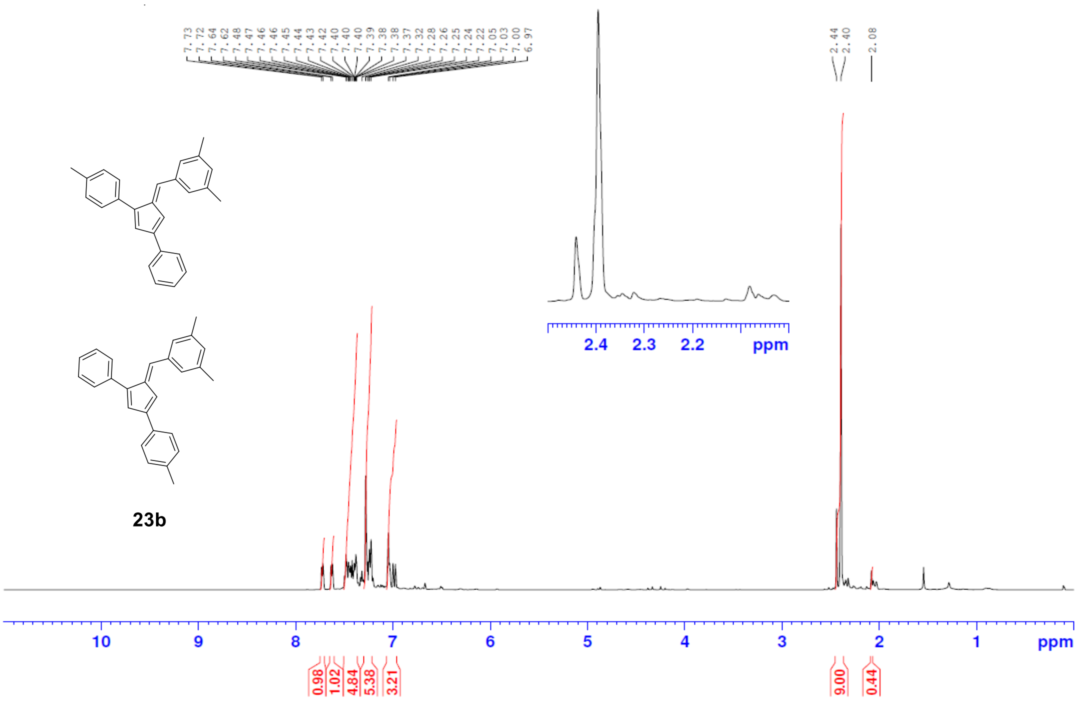

Figure S57: ^{1}H NMR of 1-(phenyl)-3-(*p*-tolyl)-6-(3,5-dimethylphenyl)fulvene and 1-(*p*-tolyl)-3-(phenyl)-6-(3,5-dimethylphenyl)fulvene **23b** (500 MHz, $CDCl_3$, 298 K)

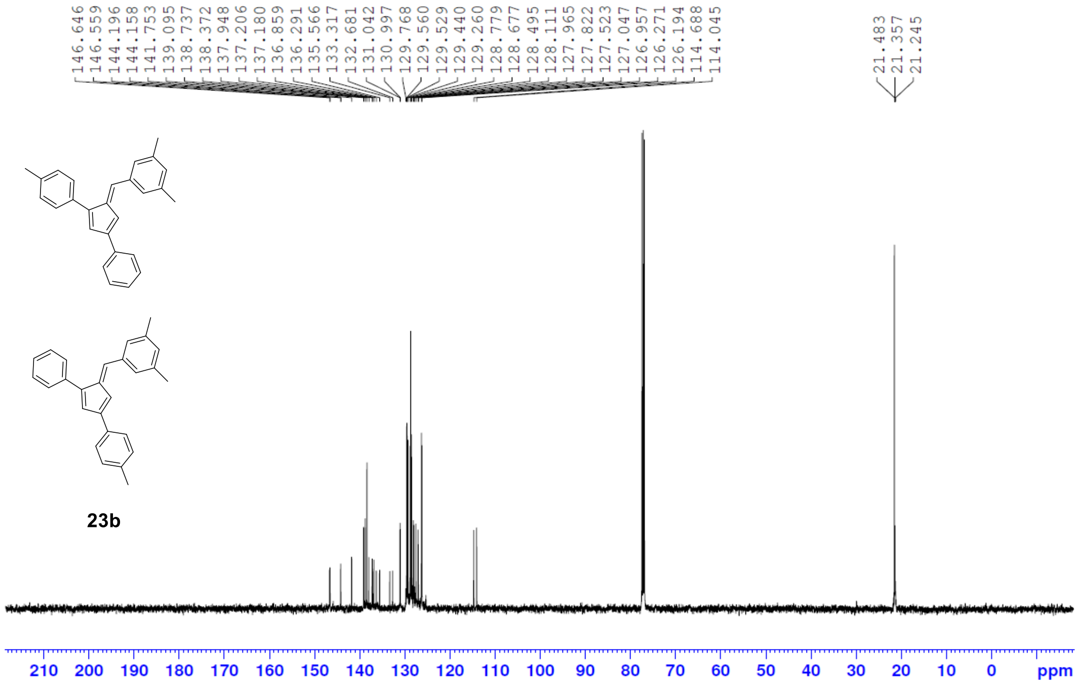

Figure S58: $^{13}C\{^{1}H\}$ NMR of 1-(phenyl)-3-(*p*-tolyl)-6-(3,5-dimethylphenyl)fulvene and 1-(*p*-tolyl)-3-(phenyl)-6-(3,5-dimethylphenyl)fulvene **23b** (125 MHz, $CDCl_3$, 298 K)

4. UV-vis spectroscopic comparison of selected dihydropentalenes and pentafulvenes

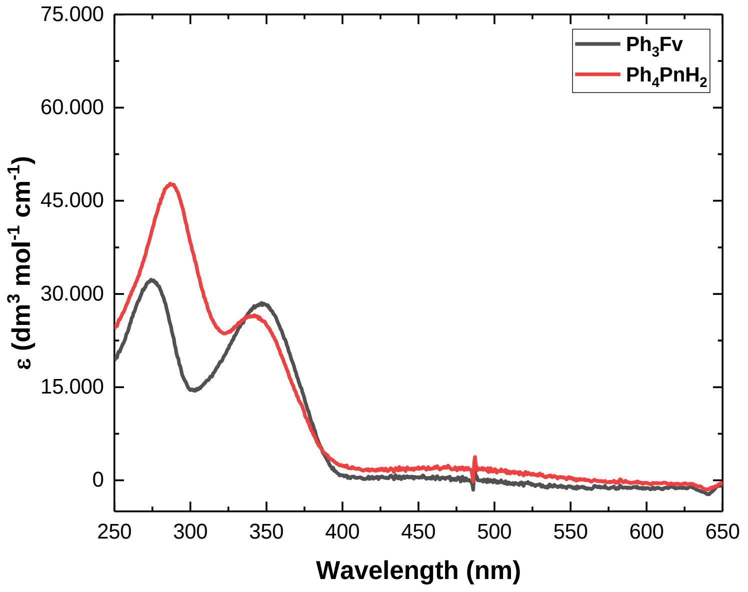

Figure S59: UV-vis spectra of **Ph_3Fv** and **Ph_4PnH_2** in CH_2Cl_2 at 293 K

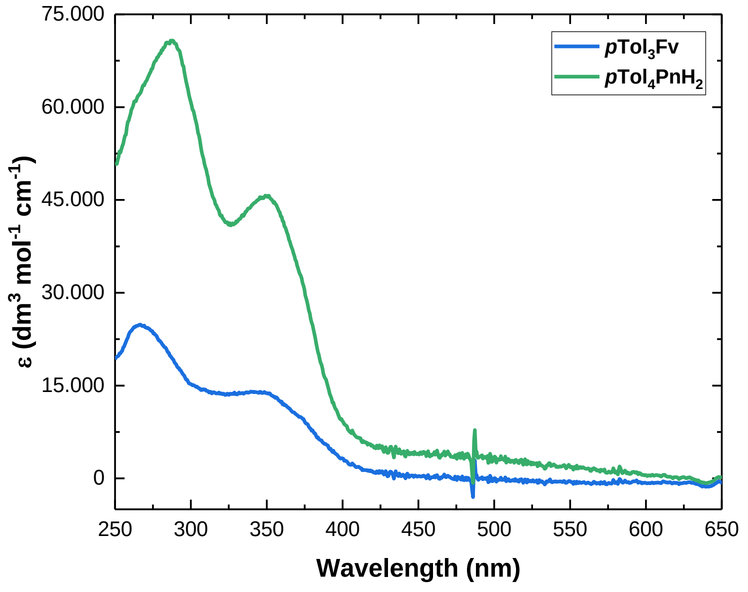

Figure S60: UV-vis spectra of ***p*Tol_3Fv** (**8b**) and ***p*Tol_4PnH_2** (**8a**) in CH_2Cl_2 at 293 K

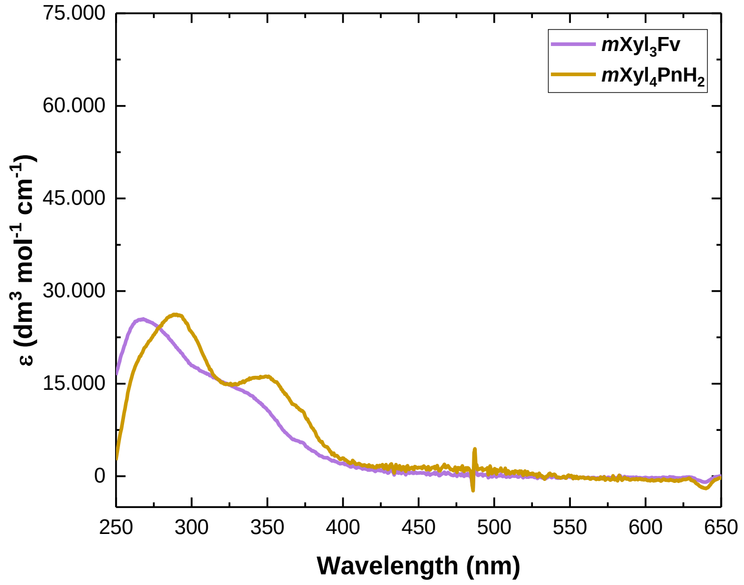

Figure S61: UV-vis spectra of ***m*Xyl$_3$Fv** (**9b**) and ***m*Xyl$_4$PnH$_2$** (**9a**) in CH_2Cl_2 at 293 K

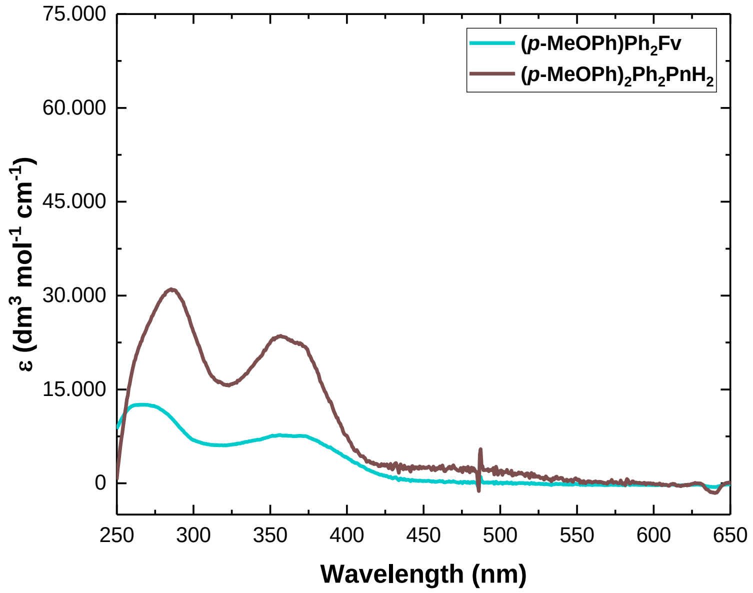

Figure S62: UV-vis spectra of **(*p*-MeOPh)Ph$_2$Fv** (**15b**) and **(*p*-MeOPh)$_2$Ph$_2$PnH$_2$** (**15a**)

in CH_2Cl_2 at 293 K
